# Polymerase Synthesis of Hypermodified DNA Displaying a Combination of Thiol, Hydroxyl, Carboxylate, and Imidazole Functional Groups in the Major Groove

**DOI:** 10.1002/chem.202501034

**Published:** 2025-05-15

**Authors:** Lukáš Kaiser, Marek Ondruš, Lenka Poštová Slavětínská, Veronika Raindlová, Michal Hocek

**Affiliations:** ^1^ Institute of Organic Chemistry and Biochemistry Czech Academy of Sciences Flemingovo nam. 2, Prague 6 Prague CZ‐16000 Czech Republic; ^2^ Department of Organic Chemistry University of Chemistry and Technology Technická 5, Prague 6 Prague 166 28 Czech Republic; ^3^ Department of Organic Chemistry Faculty of Science Charles University Hlavova 8, Prague 2 Prague CZ‐12843 Czech Republic

**Keywords:** DNA, enzymatic syntheses, nucleotides, polymerases

## Abstract

We designed and synthesized a set of six 2’‐deoxyribonucleoside 5’‐*O*‐triphosphates (dNTPs) bearing functional groups mimicking amino acid side chains in enzyme active sites (OH, SH, COOH, and imidazole) attached to position 5 of pyrimidines or position 7 of 7‐deazapurines through different linkers. These modified dNTPs were studied as substrates in enzymatic synthesis of modified and hypermodified DNA using several DNA polymerases. In primer extension (PEX), all modified dNTPs provided DNA containing one, two, three, or, (all) four modified nucleotides each bearing a different modification, although the thiol‐modified dNTPs were worse substrates compared to the others. In PCR, we observed exponential amplification for any combination of one, two, or three nonsulfur dNTPs but the thiol‐modified dNTP did not work well in any combinations. Sequencing of the hypermodified DNA confirmed the good fidelity of the incorporation of all the modified nucleotides. This set of modified dNTPs extends the portfolio of building blocks for prospective use in selections of functional nucleic acids.

## Graphical abstract

A series of 2’‐deoxyribonucleoside 5’‐O‐triphosphates bearing functional groups of amino acid side chains in enzyme active sites (OH, SH, COOH, and imidazole) were synthesized and used for enzymatic synthesis of hypermodified DNA displaying these substituents in the major groove mimicking peptides or proteins.

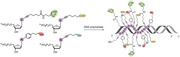

## 1 Introduction

Functional nucleic acids are often designed or selected to mimic functions of proteins. DNAzymes or RNAzymes catalyze reactions approximating or even surpassing activity of protein enzymes,^[^ ^1^ ^]^ aptamers specifically recognize and bind target molecules emulating the action of antibodies,^[^ ^2^ ^]^ whereas some DNA or RNA nanostructures can imitate structural or membrane protein complexes.^[^ ^3^ ^]^ Natural nucleic acids possess a large diversity of sequences and secondary structures, but compared to proteins, they have a rather low diversity of functional groups which limits the versatility of their applications. Therefore, chemically modified nucleic acids^[^ ^4^ ^]^ bearing additional substituents or molecules attached to nucleobase have great potential in the development of functional biomacromolecules and their assemblies. Nucleotides bearing amino‐acid‐like functional groups have been extensively studied as building blocks for selection of DNAzymes or aptamers. In base‐modified DNAzymes,^[^ ^5^ ^]^ diverse aminoalkyl‐,^[^ ^6^ ^]^ histamine‐,^[^ ^6^ ^]^ guanidine‐,^[^ ^7^ ^]^ or tyrosine‐linked^[^ ^8^ ^]^ nucleotides and their combinations were often in selections resulting in efficient RNA‐cleaving DNAzymes, while amino‐, carboxy‐, or hydroxy‐linked nucleotides were employed in amide‐cleaving DNAzymes.^[^ ^9^ ^]^ In base‐modified aptamers,^[^ ^10^ ^]^ nucleotides bearing one^[^ ^11^ ^]^ or two^[^ ^12^ ^]^ hydrophobic and/or aromatic functional groups were shown to significantly enhance the specific binding to target proteins. In all these selections, the enzymatic synthesis^[^ ^13^ ^]^ of modified DNA using DNA polymerases and base‐modified 2’‐deoxyriboncleoside triphosphates (dNTPs) is a crucial step.

Hypermodified nucleic acids are DNA or RNA oligo or polymers, where every nucleotide is bearing a modification. In the past there were only scattered examples^[^ ^14^ ^]^ of enzymatic synthesis of this class of compounds using combination of four base‐modified 2’‐deoxyribonucleoside triphosphates (dNTPs) and hence, we have systematically studied and developed robust polymerase syntheses of hypermodified DNA containing sets of four different redox labels,^[^ ^15^ ^]^ four hydrophobic groups,^[^ ^16^ ^]^ four anionic substituents,^[^ ^17^ ^]^ four cationic groups,^[^ ^18^ ^]^ or four glycosides,^[^ ^19^ ^]^ and their combinations. These hypermodified DNAs can be prepared by primer extension (up to 150 modified nucleotides) or asymmetric PCR from single‐stranded DNA templates or even by reverse transcription from RNA templates.^[^ ^20^ ^]^ Similarly, we have also prepared hypermodified RNA displaying four different substituents by primer extension of RNA or DNA primer using engineered TGK DNA polymerase and modified ribonucleoside triphosphates.^[^ ^21^ ^]^ To further complement the portfolio of modifications for display on hypermodified DNA, we have now focused on substituents mimicking amino acid side chains occurring in active sites of enzymes, that is, hydroxy‐ (analogue of Ser), carboxy‐ (analogue of Asp or Glu), imidazole‐ (analogue of His), and thiol‐linked (analogue of Cys). Previously, examples of hydroxy‐, carboxy,‐ and histamine‐linked dNTPs^[^ ^22^ ^]^ as well as proline, urea, and sulfonamide‐linked dNTPs^[^ ^23^ ^]^ were reported by M. Hollenstein, who even succeeded in enzymatic synthesis of DNA containing a combination of these three modifications.^[^ ^22^ ^]^ There were several reports on synthesis and enzymatic incorporation of sulfide‐^[^ ^24^ ^]^ or disulfide‐linked^[^ ^25^, ^26^ ^]^ dNTPs and an example of chemical synthesis of oligonucleotides containing 5‐sulfanylmethyluracil,^[^ ^27^ ^]^ but thiol‐linked dNTPs have not been known in literature until our recent work^[^ ^28^ ^]^ reporting synthesis of 5‐sulfanylmethyl‐dUTP and its use in enzymatic synthesis of modified DNA. Enzymatic incorporation of free thiol‐containing nucleotides could be challenging mainly due to the possible formation of diverse disulfides under oxidative conditions. Therefore, the enzymatic synthesis of hypermodified DNA containing free unprotected thiols and a combination of the thiol and other three active site‐like modifications is a worthwhile goal with promising potential use in selection of DNAzymes.

## 2 Results and Discussion

**Scheme 1:**
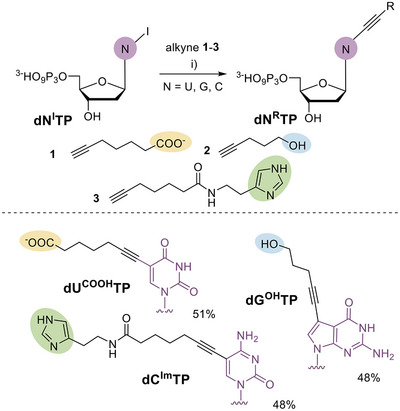
Design and synthesis of **dN^R^TP**s. Reagents and conditions: (i) **dN^I^TP**, alkyne **1**–**3** (2 equiv.), Pd(OAc)_2_ (10 mol%), TPPTS (50 mol%), CuI (20 mol%), Et_3_N (8 equiv.), MeCN/H_2_O (1:1), 1 hour, and 60 °C.

In the design of base‐modified dNTPs bearing substituents mimicking amino acid side chains commonly occurring in active sites of enzymes, we wanted to extend (rather than replicate) the portfolio of dNTPs previously reported by Hollenstein,^[^ ^22^ ^]^ and therefore we came up with new combinations of nucleobases and substituents containing carboxylic acid, alcohol and imidazole moieties. Modifications were attached to the position 5 of the pyrimidines and position 7 of the 7‐deazapurines via alkylethynyl linkers leading to the following target base‐modified dNTPs: 5‐(6‐carboxyhex‐1‐ynyl)‐dUTP (**dU^COOH^TP**), 5‐(5‐hydroxypent‐ynyl)‐7‐deaza‐dGTP (**dG^OH^TP**) and 5‐(6‐histaminocarbonylhex‐1‐ynyl)‐dCTP (**dC^Im^TP**). The synthesis was based on the aqueous‐phase Sonogashira cross‐coupling reactions^[^ ^29^ ^]^ of 5‐iodopyrimidine or 7‐deaza‐7‐iodo‐guanine 2’‐deoxyribonucleoside 5’‐*O*‐triphosphates (**dN^I^TP**s)^[^ ^30^ ^]^ with the corresponding terminal alkynes **1**–**3** (Scheme 1 and Scheme S1 in Supporting Information) affording the desired products in good yields (48%– 51%) after HPLC purifications.

**Scheme 2:**
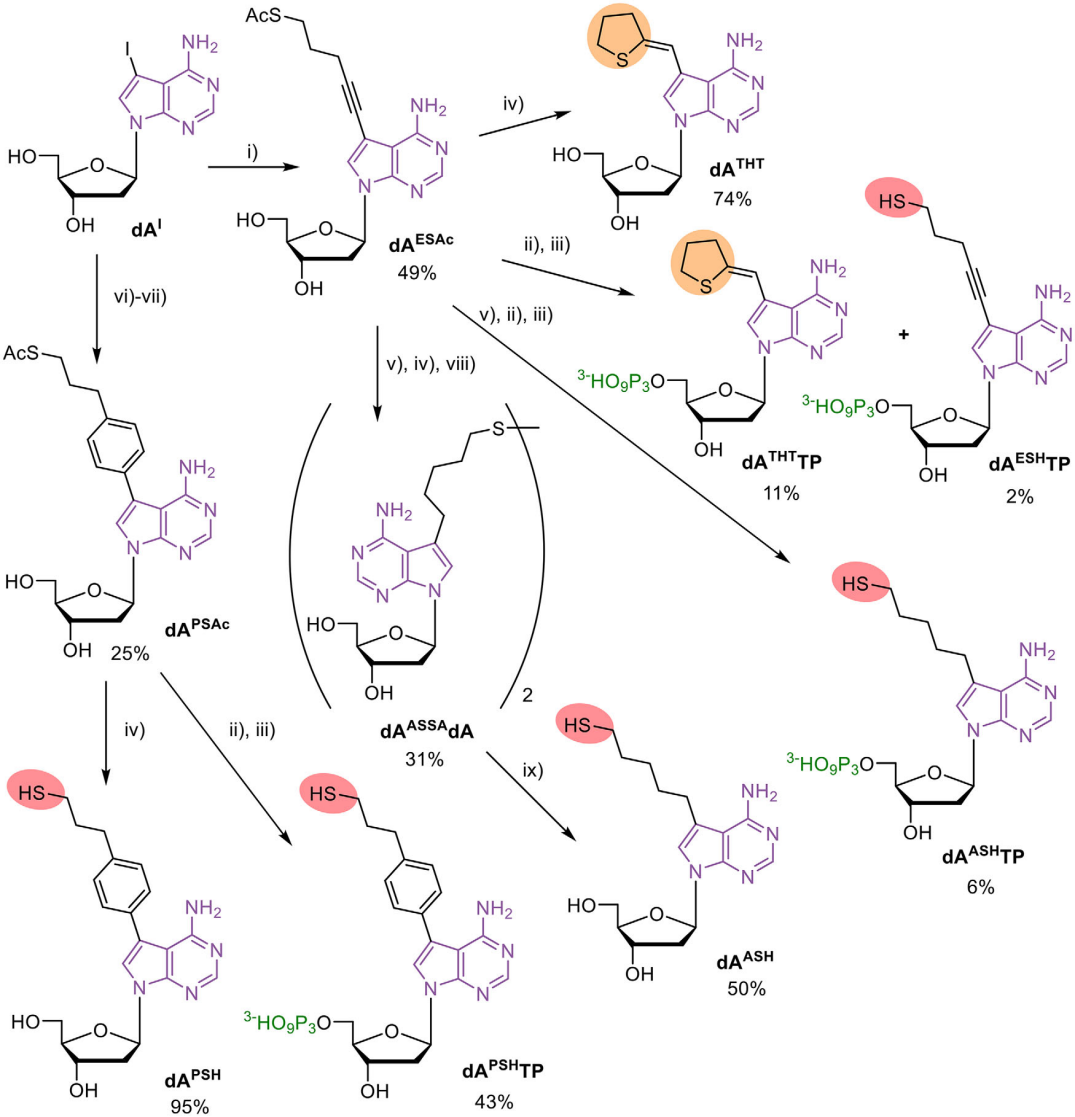
Synthesis of sulfur‐modified nucleosides and dNTPs. Reagents and conditions: (i) **dA^I^** (1 equiv.), 5‐(acetylthio)‐pent‐1‐yne (**4**) (1.2 equiv.), Pd(PPh_3_)_4_ (10 mol.%), CuI (20 mol.%), triethylamine (2.5 equiv.), DMF, under argon, overnight, r.t.; (ii) 1) POCl_3_ (1.2 equiv.), PO(OMe)_3_, −10 °C, 2 hours, under Ar; 2) (NHBu_3_)_2_H_2_P_2_O_7_ (5 equiv.), Bu_3_N (4 equiv.), DMF, −10 °C, 1 h, under Ar; 3) 2 M TEAB; (iii) 25% NH_3_ (aq.), 1.5 hours, r.t.; (iv) 25% NH_3_ (aq.) in MeOH (1:9), 2 hours, r.t.; (v) Pd/C (1 equiv.), H_2_, MeOH, 8 hours, reflux; (vi) boronate ester (**5**) (1 equiv.), **dA^I^** (0.9 equiv.), Na_2_CO_3_ (3 equiv.) MeCN/H_2_O (4:1), 45 minutes, 90 °C; (vii) crude product from (v), potassium thioacetate (1 equiv.), acetone, 3 hours, reflux; (viii) oxidation by air, MeOH, overnight, r.t.; (ix) TCEP·HCl (1.5 equiv.), MeOH/H_2_O (4:1), 1 h, r.t. For more details see Supporting Information, Parts 1.1. and 1.2.

Then we planned to explore the syntheses of hitherto unknown mercapto derivatives (analogues of Cys side chain) of dNTPs and decided to attach the sulfanyl moiety to position 7 of 7‐deaza‐dATP through different tethers. Here, we envisaged to first perform cross‐coupling reactions with 7‐iodo‐2’‐deoxy‐7‐deazaadenosine (**dA^I^**) nucleoside followed by triphosphorylation in the second step. We started by the Sonogashira cross‐coupling of **dA^I^** with corresponding S‐acetyl‐protected 5‐(acetylthio)‐pent‐1‐yne (**4**)^[^ ^31^ ^]^ (Scheme 2, Scheme S1 in Supporting Information) in DMF in presence of Pd(PPh_3_)_4_, CuI, and Et_3_N which gave the modified nucleoside **dA^ESAc^** in 49% yield. The follow up triphosphorylation following standard protocol^[^ ^32^ ^]^ and the removal of the protecting acetyl group by 25% aqueous ammonia (aq. NH_3_) gave only trace amounts of expected thiol‐linked **dA^ESH^TP** while the major product (isolated in 11% yield) was the cyclized tetrahydrothiophene‐linked nucleotide **dA^THT^TP** as the product of intramolecular addition of thiol to the alkyne triple bond. We have also tried to deprotect the acetylated nucleoside **dA^ESAc^** using analogous reaction with aq. NH_3_ and also here we observed formation of the cyclized product **dA^THT^** in 74% yield. Since apparently the mercaptopropyl‐alkyne is unstable and prone to intramolecular cyclization, we performed catalytic hydrogenation of the acetylated nucleoside **dA^ESAc^** using H_2_ on Pd/C in methanol under reflux. This reaction led to an inseparable mixture of reduced protected thiol and unwanted desulfurized 7‐pentyl‐2’‐deoxy‐7‐deazaadenosine. Therefore, the crude product mixture after HPFC chromatography was dried and directly used in follow up triphosphorylation and thiol deprotection. The desired 7‐(5‐sulfanylpentyl)‐7‐deaza‐dATP (**dA^ASH^TP**) was then isolated by HPLC in moderate overall yield of 6% (over three steps). We also tried to do the deprotection of the crude nucleoside after catalytic hydrogenation with aq. NH_3_ in methanol but we were unable to isolate the free thiol nucleoside. Instead, we oxidized the crude product with air and managed to isolate the disulfide **dA^ASSA^dA** by HPFC in 31% yield after HPFC chromatography. Subsequent reduction of disulfide with tris(2‐carboxyethyl)phosphine (TCEP) hydrochloride in MeOH/H_2_O gave the free‐thiol nucleoside **dA^ASH^** after reverse‐phase HPFC in 50% yield.

Because of the difficulties in the synthesis and reactivity of the alkyne and alkyl linked thiol dATP derivatives, we also tried to attach the sulfanylalkyl group to 7‐deazaadenine through rigid and conjugate but unreactive phenylene linker and introduce it by the Suzuki‐Miyaura reaction with the corresponding arylboronate ester. To avoid possible poisoning of Pd catalyst by free thiol that would be at least partially formed under basic conditions of the Suzuki reaction, we performed the Suzuki reaction of **dA^I^** with 4‐(3‐bromopropyl)phenylboronate ester (**5)** ^[^ ^33^ ^]^ (Scheme S1 in Supporting Information) followed by the reaction with potassium thioacetate to afford the 7‐[4‐(acetylsulfanylpropyl)phenyl]‐7‐deaza‐2’‐deoxyadenosine (**dA^PSAc^**) in 25% yield which was also deprotected to give 7‐[4‐(sulfanylpropyl)phenyl]‐7‐deaza‐2’‐deoxyadenosine (**dA^PSH^**). The triphosphorylation of acetyl‐protected nucleoside **dA^PSAc^** followed by treatment with aq. NH_3_ gave the desired **dA^PSH^TP** in 43% yield after HPLC purification.

**Figure 1:**
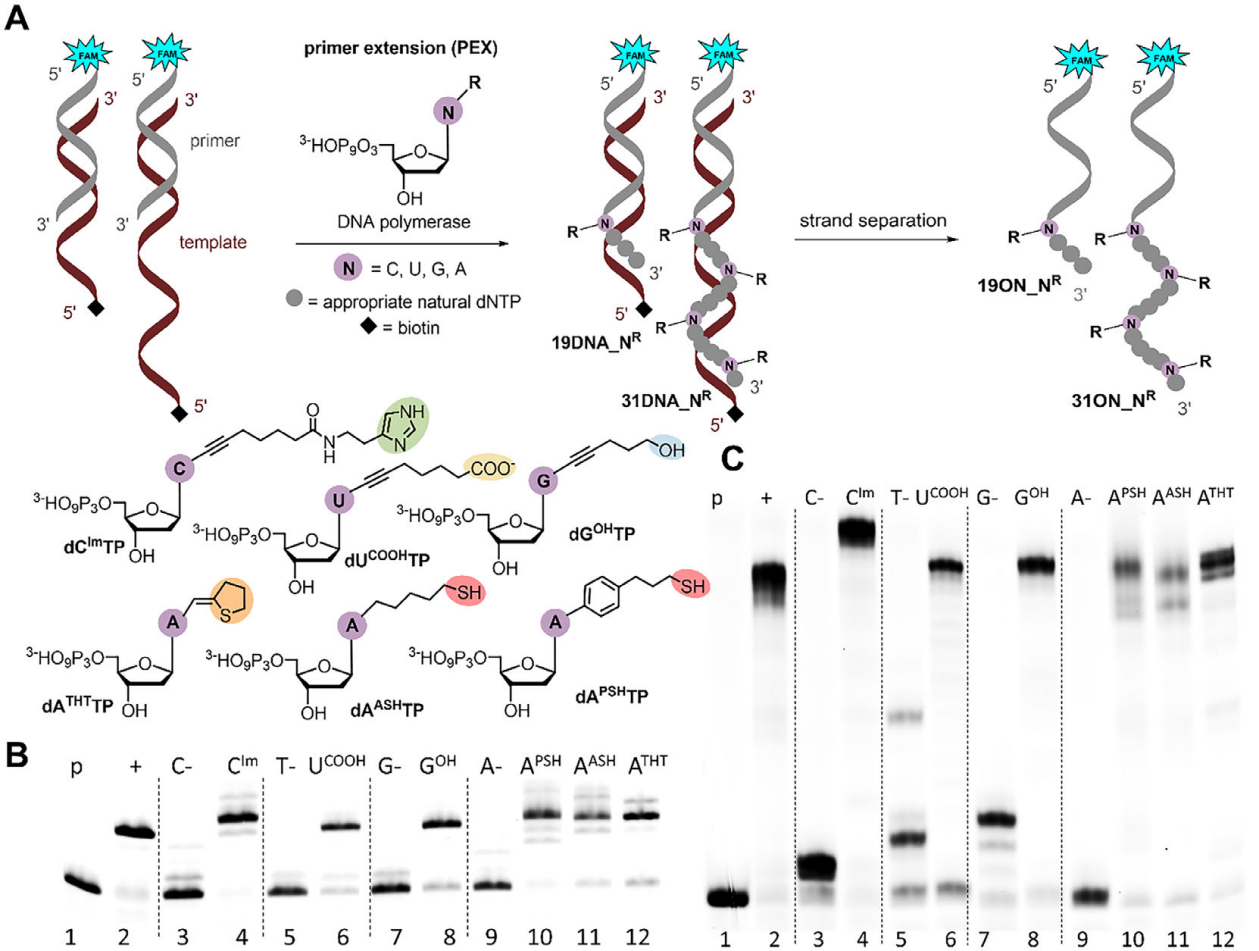
(A) Synthetic scheme of modified DNAs (and ONs) syntheses by PEX reaction using one modified dN^R^TP. (B) dPAGE analysis of PEX using Vent(exo–) DNA polymerase, 5′‐(6‐FAM)‐labelled primer, and 19‐mer appropriate template; (C) dPAGE analysis of PEX using Pwo DNA polymerase, 5′‐(6‐FAM)‐labelled primer, and 31‐mer template, lane (1) primer; lane (2) positive control (PEX using four natural dNTPs); lanes (3), (5), (7), and (9) negative controls in absence of modified or natural dCTP, dTTP, dGTP, or dATP; lanes (4), (6), (8), and (10)–(12) PEX reactions using dC^Im^TP, dU^COOH^TP, dG^OH^TP, dA^PSH^TP, dA^ASH^TP, or dA^THT^TP in combination with the other three natural dNTPs. For reaction details see Supporting Information, Parts 2.1.1. and 2.1.2.

Then, the six base‐modified triphosphates which were obtained in sufficient quantities (**dC^Im^TP**, **dG^OH^TP**, **dU^COOH^TP**, **dA^PSH^TP**, **dA^ASH^TP,** and **dA^THT^TP**) were tested as substrates for DNA polymerases in primer extension (PEX). Prior to biochemical profiling, the thiol‐linked nucleotides (**dA^PSH^TP** and **dA^ASH^TP**) were treated with TCEP to reduce any disulfides. At first, the PEX reactions were performed separately for each modified **dN^R^TP** in combination with corresponding natural dNTPs (Figure 1A). We used a 15‐mer primer and two different templates: a 19‐mer template, encoding incorporation of one modified triphosphate and a 31‐mer template encoding for four incorporations of each nucleotide (for sequences see Supporting Information, Table S1). Thermostable Vent(exo‐) DNA polymerase (lacking the 3’→5’ proofreading exonuclease activity) showed the best incorporation results on denaturing polyacrylamide gel (dPAGE) for 19‐mer template providing **19DNA_N^R^** (R = Im, OH, COOH, PSH, ASH, and THT) full‐length products (Figure 1B, and in SI, Table S2, Part 2.1.1.), whereas for the longer 31‐mer template, Pwo DNA polymerase (thermostable polymerase with 3′→5′ exonuclease activity), showed the most efficient extension of the primer giving a single full‐length products of **31DNA_N^R^** (Figure 1C, and in Supporting Information, Table S2, Part 2.1.2.). The thiol‐linked dNTPs (**dA^PSH^TP** and **dA^ASH^TP**) were somewhat worse substrates giving either smeared or less pure product with the longer 31‐mer template (lanes 10 and 11 in Figure 1C). In the case of **dA^ASH^TP** incorporation, the presence of shorter byproducts visible on dPAGE was also supported by lower mass signals in mass spectrum of **31ON_A^ASH^** (Figure S33 in SI). For **31ON_A^PSH^** no significant shorter byproducts signals were observed by MALDI (Figure S32 in SI), suggesting the smeared product band might be a feature of the free thiol group under dPAGE analysis. The PEX was also successful with KOD XL DNA polymerase (Figure S2 and S4 in Supporting Information). For MALDI‐TOF characterization of modified oligonucleotides (ONs), the PEX was performed in larger scale with biotinylated templates followed by magnetoseparation using streptavidin‐coated magnetic beads (Chapter 2.2 in Supporting Information). In all cases, the correct mass of the full‐length products of **19ON_N^R^** and **31ON_N^R^** (R = Im, OH, COOH, PSH, ASH, and THT) was confirmed by MALDI (Table S6, Figures S23 and S34. in Supporting Information).

**Figure 2:**
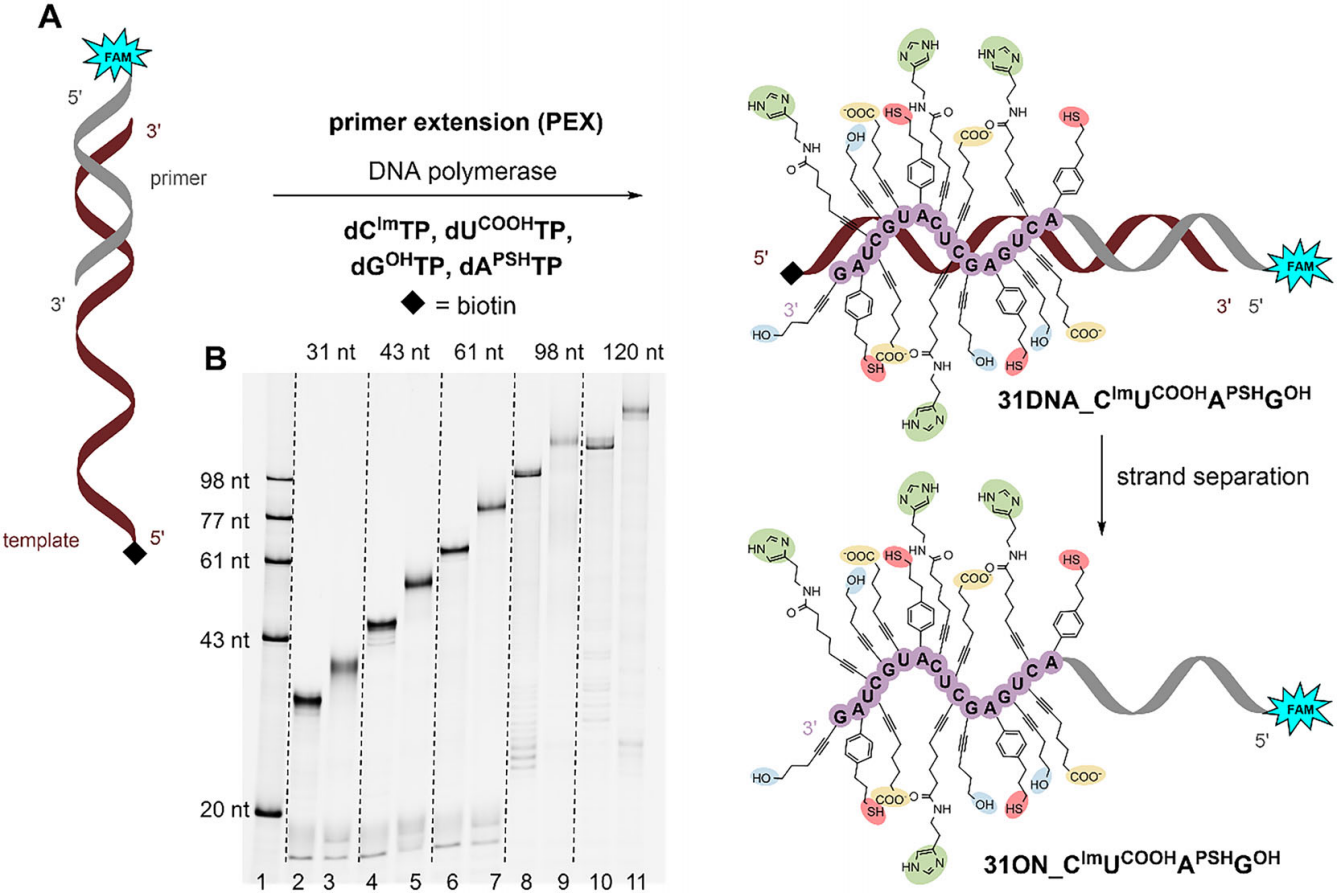
(A) Synthesis of hypermodified DNA by PEX reaction using a combination of **dC^Im^TP**, **dG^OH^TP**, **dU^COOH^TP,** and **dA^PSH^TP**. (B) dPAGE analysis of PEX reactions using four different modifications with different template length, lane (1) single‐stranded ladder; lanes (2), (4), (6), (8), and (10) positive controls – PEX reactions with natural dNTPs; lanes (3), (5), (7), (9), and (11) hypermodified PEX products using the combination of all four modified **dN^R^TPs**. For reaction details see Supporting Information, Parts 2.1.5. and 2.1.6.

Next, we tested whether the polymerases will be able to synthesize hypermodified DNA using a combination of all four modified **dN^R^TPs** (Figure 2). To this end, we selected **dC^Im^TP**, **dG^OH^TP**, **dU^COOH^TP** and **dA^PSH^TP**, and Pwo DNA polymerase and aforementioned 31‐mer template and 15‐mer primer. The full‐length hypermodified product **31DNA_C^Im^U^COOH^A^PSH^G^OH^** was observed on dPAGE (Figure 2B, lane 3) and confirmed by MALDI‐TOF analysis, again after using biotinylated template and magnetoseparation (**31ON_C^Im^U^COOH^A^PSH^G^OH^**, Table S6, Figure S35 in Supporting Information). We have also successfully prepared hypermodified DNA using combinations of **dN^R^TPs** containing **dA^ASH^TP** or **dA^THT^TP** (Figure S9 in Supporting Information) and the correct mass of the full‐length products was confirmed by MALDI again using biotinylated template and magnetoseparation (Table S6, Figures S36 and S37 in Supporting Information). To test whether we can prepare longer hypermodified DNA, we performed the PEX reactions with longer templates (43, 61, 98, and 120nt) with Pwo DNA polymerase and the set of four modified nucleotides (**dC^Im^TP**, **dG^OH^TP**, **dU^COOH^TP**, and **dA^PSH^TP**). In all cases, we observed the formation of fully extended products on dPAGE gel (Figure 2B, lanes 5, 7, 9, and 11, and in Supporting Information, Figures S8–S10, Parts 2.1.5, 2.1.6.), confirming that this combination of modified **dN^R^TPs** is suitable for polymerase synthesis of even longer hypermodified polymers **43/61/98/120DNA_C^Im^U^COOH^A^PSH^G^OH^**.

**Figure 3:**
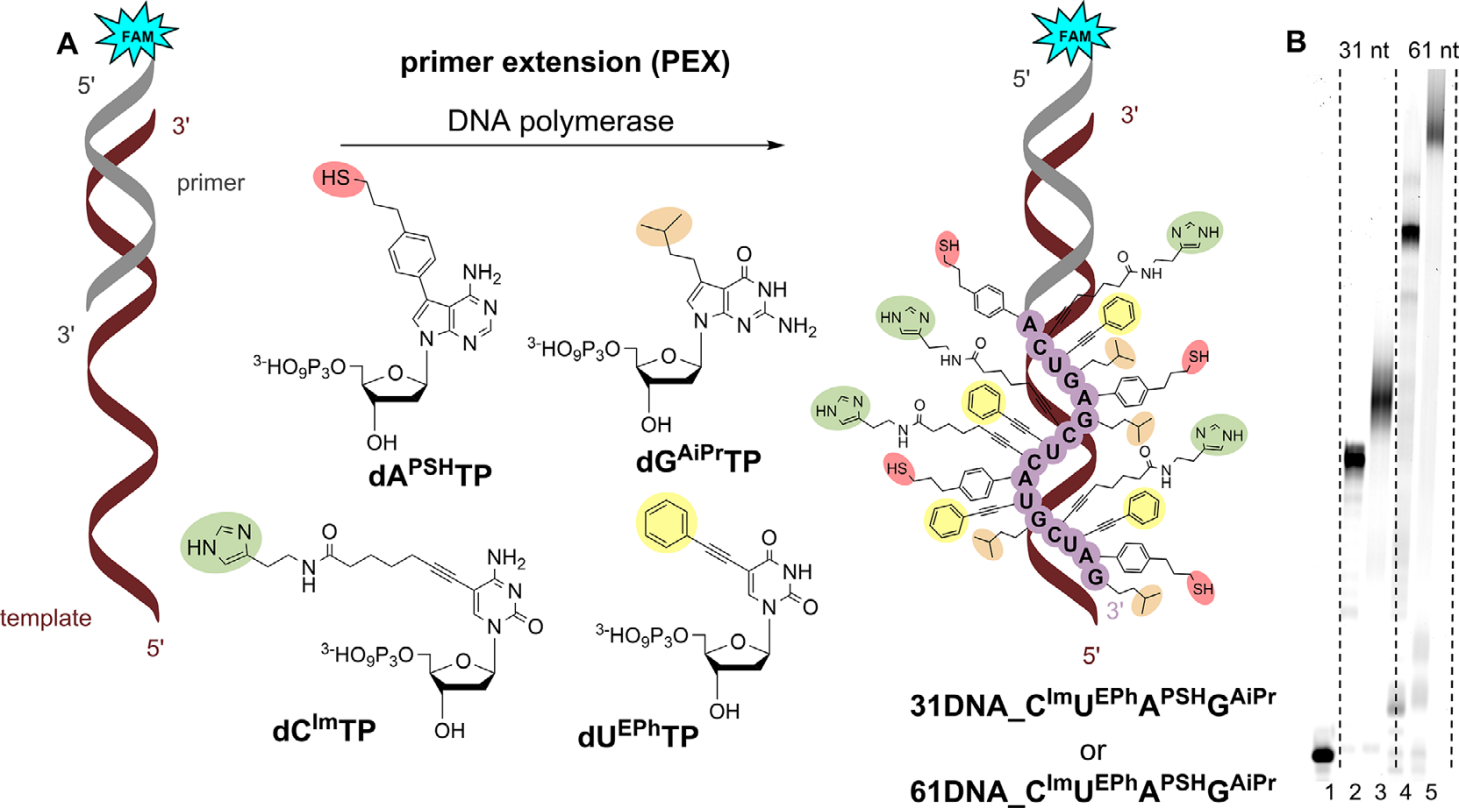
(A) Synthesis of hypermodified DNA by PEX reaction using a combination of **dC^Im^TP**, **dG^AiPr^TP**, **dU^EPh^TP,** and **dA^PSH^TP**, **(B)** dPAGE analysis of PEX reactions using four different modifications (**dC^Im^TP**, **dU^EPh^TP**, **dG^AiPr^TP, and dA^PSH^TP**) with different template length, lane (1) primer; lanes (2), (4) positive controls – PEX reactions with natural dNTPs; lanes (3), (5) hypermodified DNA products **31DNA_C^Im^U^EPh^A^PSH^G^AiPr^** and **61DNA_C^Im^U^EPh^A^PSH^G^AiPr^**. For reaction details see SI, Parts 2.1.7. and 2.1.8.

We also attempted to synthesize hypermodified DNA bearing two protein‐like **dN^R^TPs**: **dC^Im^TP**, **dA^PSH^TP** in combination with two previously reported^16^ 2´‐deoxyribonucleoside triphosphates **dU^EPh^TP** and **dG^AiPr^TP** bearing different hydrophobic modifications – phenyl and isopropyl – attached to the nucleobase via ethynyl‐ and alkyl‐linker, respectively (Figure 3A). We performed the PEX reaction with templates of two lengths: 31‐mer and 61‐mer using KOD XL DNA polymerase showing the applicability in incorporation of this combination for longer templates (Figure 3B, and in SI, Figures S11‐S12). In both cases, full‐length products **31/61DNA_C^Im^U^EPh^A^PSH^G^AiPr^** were observed. The correct mass of the full‐length 31nt long product was confirmed by MALDI after strand separation using biotinylated template in PEX giving **31ON_C^Im^U^EPh^A^PSH^G^AiPr^** (Table S6, Figure S38 in SI).

**Figure 4:**
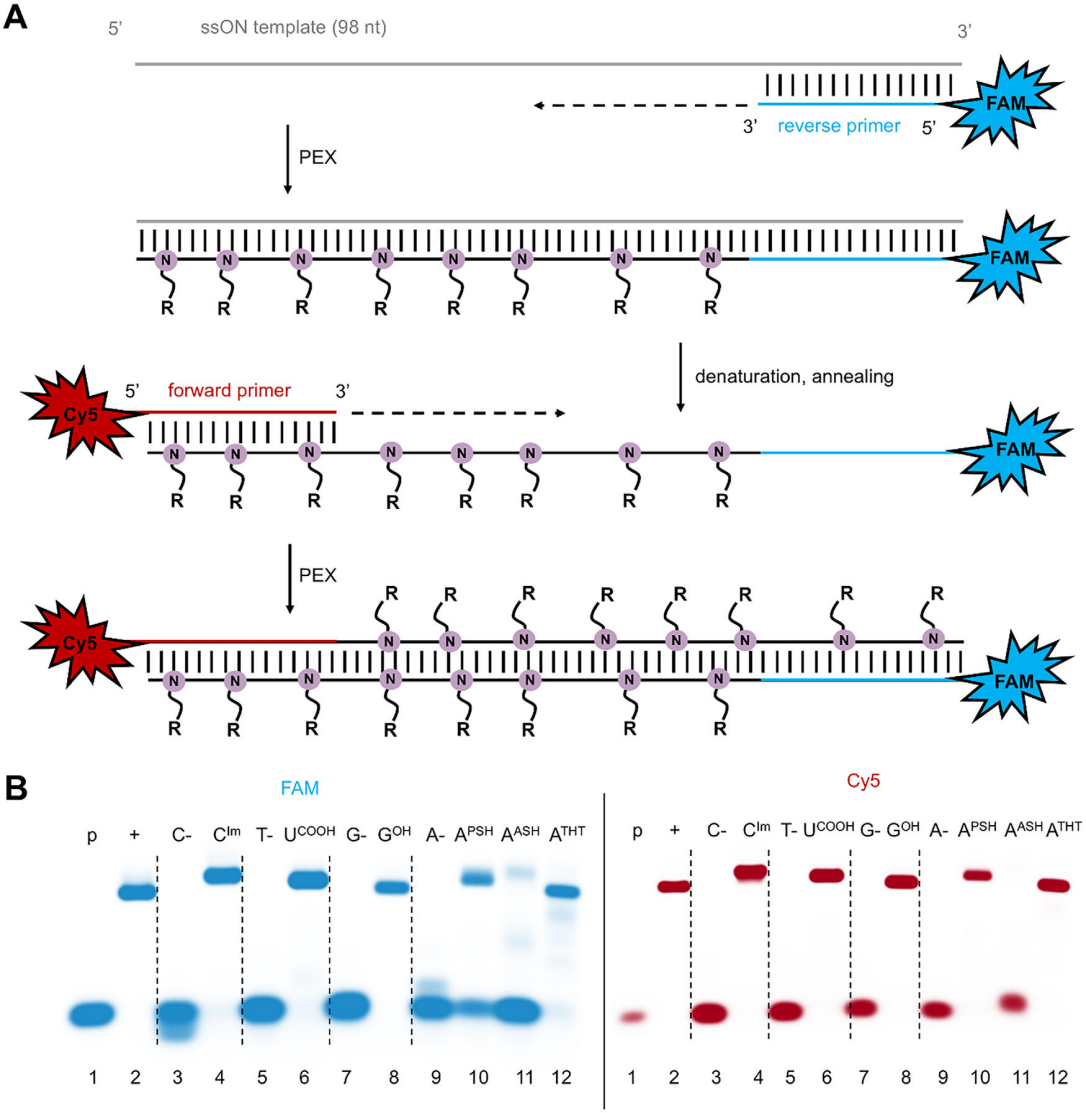
(A) Scheme of reverse and forward primers extension in PCR. (B) Native agarose gel analysis of PCR experiments with one modified **dN^R^TP** using 5′‐(6‐FAM)‐labelled reverse primer, 5′‐Cy5‐labelled forward primer, 98‐mer template, and KOD XL DNA polymerase; lanes (1) primer; (2) positive control – reaction with natural dNTPs; (4), (6), (8), and (10‐12) reactions using **dC^Im^TP**, **dU^COOH^TP**, **dG^OH^TP**, **dA^PSH^TP**, **dA^ASH^TP**, and **dA^THT^TP** in combination with the other three natural dNTPs; (3), (5), (7), and (9) negative controls, in absence of modified or natural dCTP, dTTP, dGTP, or dATP. For more details see SI, Chapter 2.4.

After successful PEX experiments that confirmed acceptable substrate activity of the modified **dN^R^TPs** with DNA polymerases, we studied them in more challenging PCR experiments. For an exponential amplification in PCR, the modified **dN^R^TPs** must not only serve as good substrates for the DNA polymerase but also the polymerase needs to be able to read through the hypermodified strand and use it as a template in further cycles. We used a 98‐mer single‐stranded DNA template in presence of differently labelled primers (for sequences see Table S1 in Supporting Information). The reverse primer was labelled with 6‐FAM and the forward primer labelled with Cy5 (Figure 4A). The PCR amplification experiments were performed using KOD XL DNA polymerase. When using one modified **dN^R^TP** in combination with three natural dNTPs, we could observe clean formation of the amplified full‐length products **98PCR_N^R^** with significantly lower mobility on the agarose gel compared to natural counterpart **98PCR** (Figure 4B, and in Supporting Information Figure S13) and visible in both FAM and Cy5 channels confirming that it is an exponential amplification with equal extension of both primers. However, thiol‐modified triphosphates gave somehow less convincing results. In case of **dA^PSH^TP**, in addition to the full‐length product **98PCR_A^PSH^** we could observe substantial amount of residual unreacted primers, whereas with **dA^ASH^TP** we could barely observe any product formation. On the other hand, **dA^THT^TP** worked as good as the other nonsulfur **dN^R^TP**s. To evaluate whether the lower amplification yields of PCR products containing thiol‐modified **dN^R^TP**s are caused by possible inhibition of the DNA polymerase (KOD XL) by these thiol‐modified **dN^R^TP**s (through possible disulfide cross‐linking with the polymerase), we performed PCR and PEX experiments in the presence of either **dA^PSH^TP** or **dA^ASH^TP** and mixture of all four natural dNTPs. We did not observe any reduced amplification in PCR or formation of full‐length product in PEX (compared to all natural PCR) indicating that the inhibition of the polymerase is not the main reason for the poor outcome (Figures S14 and S5 in Supporting Information). To assess the redox behavior of the **98PCR_A^PSH^**, we purified the amplification product DNA and let it be oxidized by air and analyzed by native agarose gel electrophoresis (Figure S22 in Supporting Information). We observed some minor slower‐mobility bands that decreased when using reducing agents (TCEP or DTT). These bands apparently correspond to some disulfide dsDNA side‐products indicating that disulfide formation on DNA during the polymerase reactions might be the reason for the poor performance of the thiol‐linked nucleotides in PCR. When comparing the thiol dNTPs, the phenyl‐linked nucleotide **dA^PSH^TP** is a better substrate for polymerases than and alkyl‐linked **dA^PSH^TP** which is in accord with previous findings showing that flexible non‐conjugate alkyl‐substituted dNTPs are typically worse substrates compared to conjugate alkenyl, alkynyl or aryl‐substituted dNTPs.^6,13,25^

**Figure 5:**
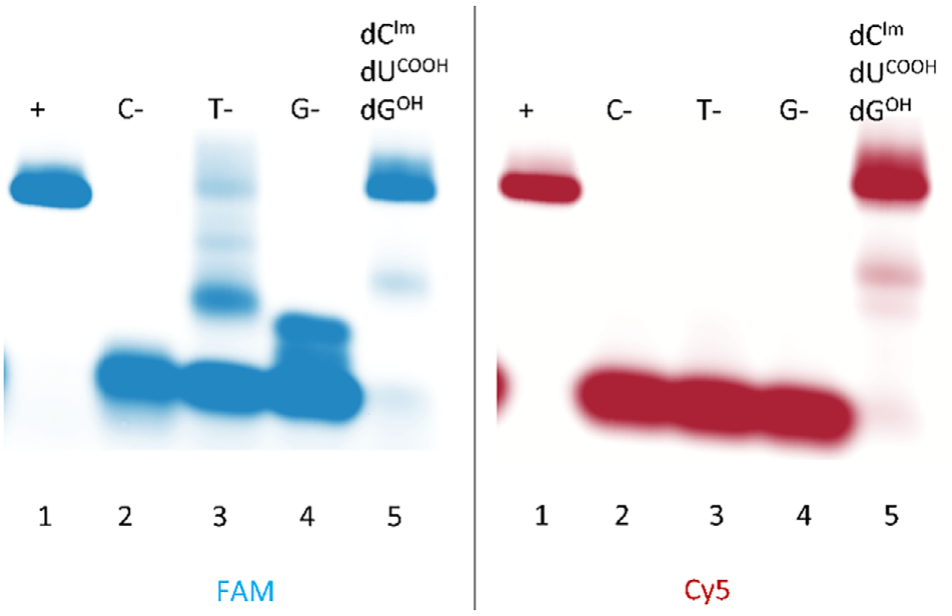
Native agarose gel analysis of PCR experiments with three modified **dN^R^TPs** (**dC^Im^TP**, **dU^COOH^TP**, **dG^OH^TP**) using 5′‐(6‐FAM)‐labelled reverse primer, 5′‐Cy5‐labelled forward primer, 98‐mer template, and KOD XL DNA polymerase; (1) positive control – reaction with natural dNTPs; (2) **dU^COOH^TP**, **dG^OH^TP**, dATP; (3) **dC^Im^TP**, **dG^OH^TP**, dATP; (4) **dU^COOH^TP**, **dG^OH^TP**, dATP; (5) **dC^Im^TP**, **dU^COOH^TP**, **dG^OH^TP**, dATP.

In the next step, we attempted a more demanding task – incorporation of a combination of several modified nucleotides. The combinations of two or three modified triphosphates **dC^Im^TP**, **dG^OH^TP**, **dU^COOH^TP** worked well with this polymerase and full‐length products could be observed on agarose gel in each case (Figure 5, and Figures S15A and S16A in SI, Parts 2.4.2., 2.4.3.). We also tested the combination of thiol modified **dA^PSH^TP** together with another one (Figure S15B in Supporting Information, Part 2.4.2.) or two (Figure S16B in Supporting Information, Part 2.4.3) modified **dN^R^TP**s. In some cases, we could observe minor bands of full‐length product but generally the efficiency was much lower compared to the other combinations of **dN^R^TP**s. We also attempted to perform PCR with the combination of all four modified nucleotides **dC^Im^TP**, **dG^OH^TP**, **dU^COOH^TP** and **dA^PSH^TP**, but even with higher amounts of polymerase we could barely see any hypermodified product on agarose gel (Figure S17 in SI, Part 2.4.4). Apparently, even the phenyl‐linked thiol **dA^PSH^TP**, which worked well in PEX in combination with modified nucleotides and reasonably well in PCR in combination with natural nucleotides (vide supra), is problematic for PCR amplification in combination with other three modified dNTPs. Since the previously published mixed disulfide‐linked dNTPs were reported^25^ to work relatively well in PCR, perhaps the use of disulfide protection of dNTPs followed by reduction could be considered an alternative way to synthesize thiol‐linked hypermodified DNA in the future.

**Figure 6:**
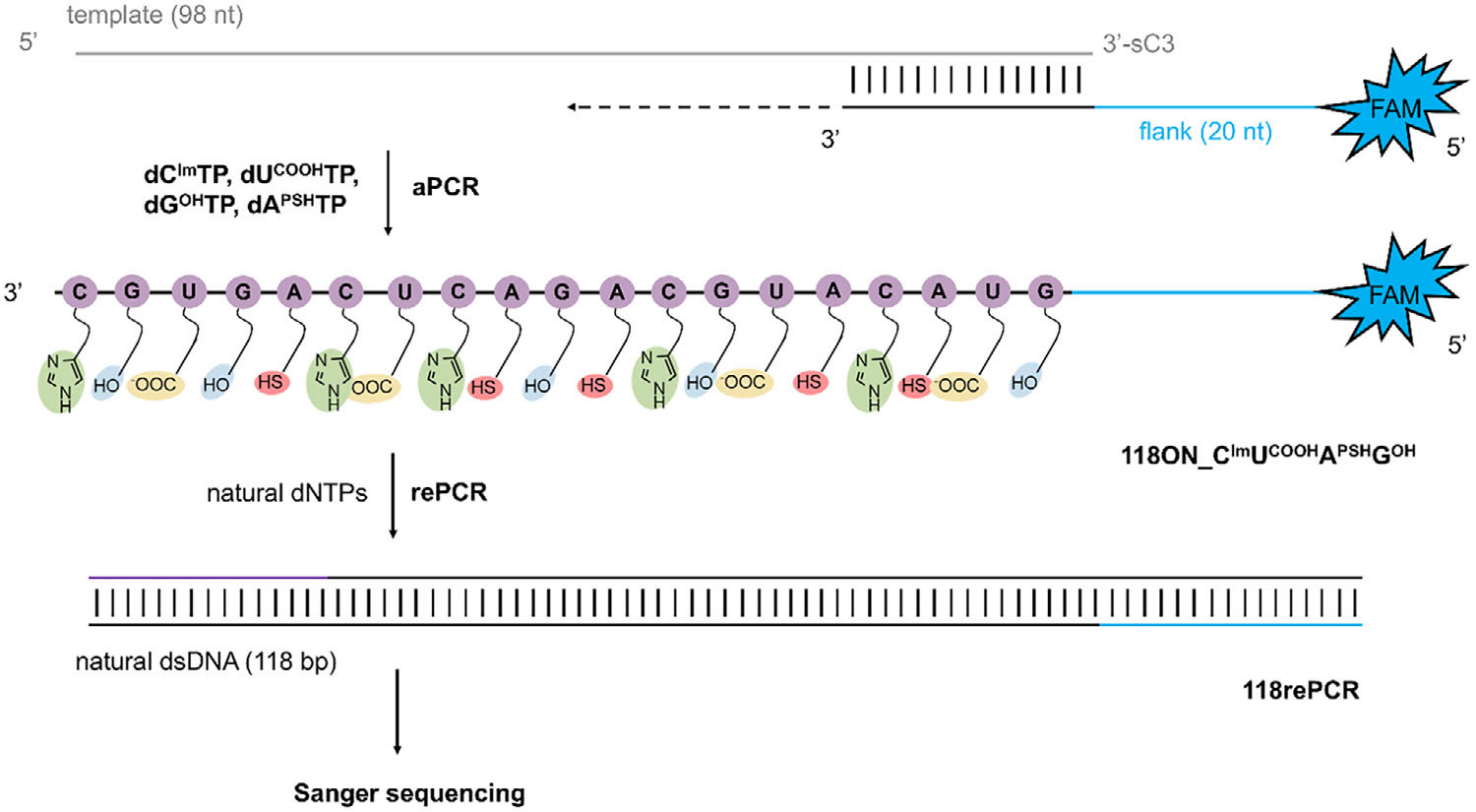
Scheme of asymmetric PCR (aPCR) using set of four modified **dN^R^TPs**, followed by rePCR with natural dNTPs to prepare dsDNA for Sanger sequencing.

For the prospective in vitro selection applications, the modified ONs need to be sequencable and show high fidelity. To test this, we used the method previously developed by our group^16^ which ensures that only the modified strand is amplified and sequenced rather than the natural template strand. In order to meet this requirement, the template is modified at 3’‐end with three carbon spacer (sC3) preventing unwanted extension during asymmetric PCR (aPCR). The primer sequence was extended by a 20‐mer overhang region, which serves as a new primer binding site for subsequent rePCR with natural dNTPs^16,17^ (for sequences see SI, Table S1). This primer was extended in aPCR reaction in the presence of 98‐mer template, set of modified triphosphates (**dC^Im^TP**, **dG^OH^TP**, **dU^COOH^TP**, **dA^PSH^TP**) and KOD XL DNA polymerase (Figure 6 and Figure S18 in Supporting Information, Part 2.5). To purify the aPCR product, Agencourt AMPure XP magnetic particles were used (Figure S19 in Supporting Information). The resulting purified hypermodified **118ON_C^Im^U^COOH^A^PSH^G^OH^** was used as a template for subsequent re‐PCR reaction in presence of natural dNTPs and KOD XL DNA polymerase (Figure S20 in Supporting Information, Part 2.6). The natural DNA product **118rePCR** was then subjected to Sanger sequencing which confirmed the high fidelity of DNA polymerase in the replication step of rePCR (Figure S21 in Supporting Information, Part 2.7).

## 3 Conclusions

We designed and synthesized a set of six modified **dN^R^TP**s bearing functional groups mimicking amino acids side chains (namely carboxylate, hydroxy group, imidazole and thiol) attached to the 5‐position of the pyrimidines and 7‐position of the 7‐deazapurines via various linkers. The modified **dN^R^TP**s proved to be moderate‐to‐good substrates for several DNA polymerases in primer extension reactions incorporating from one to combination of four different modified triphosphates resulting in hypermodified DNA displaying protein‐like functional groups in the major groove. We were able to synthesize hypermodified DNA strands with up to 95 modified nucleotides in a row. Moreover, we prepared hypermodified DNA bearing a combination of two protein‐like and two hydrophobic modifications. In PCR, exponential amplification of both strands worked well when using one, two or even three non‐sulfur modified **dN^R^TP** but the thiol‐modified **dN^R^TP**s showed lower efficiency and also did not work in combination with other modified **dN^R^TP**s. For the prospective use in selection of functional nucleic acids, we prepared single‐stranded hypermodified ON (by PEX or aPCR) which served as template for rePCR with natural dNTPs and sequencing which confirmed good fidelity of DNA polymerase in PEX or aPCR reactions. The presented set of new modified **dN^R^TP**s extends the portfolio of available modified nucleotides suitable for prospective applications in selection of DNAzymes and other functional nucleic acids.

## Supporting Information

Supporting information is available for this paper and includes additional figures and schemes, uncropped gels, complete experimental part and copies of NMR and MALDI spectra. Raw data are available at: https://doi.org/10.48700/datst.2aqm7‐66s35.

## Conflict of Interests

The authors declare no conflicts of interest.

## Supporting information



Supporting Information

## Data Availability

The data that support the findings of this study are available in the supporting information of this article.
