# Supplementary material for: Polymerase Synthesis of Hypermodified DNA Displaying a Combination of Thiol, Hydroxyl, Carboxylate, and Imidazole Functional Groups in the Major Groove
Source: Chemistry. 2025 May 15;31(34):e202501034. doi: 10.1002/chem.202501034 (PMC12172604; doi:10.1002/chem.202501034)
Supplement: Supplementary file 1 — Supporting Information [file CHEM-31-e202501034-s001.pdf]

Supplementary

Table of Contents

1. Experimental section – organic chemistry 2
   1.1. Synthesis of modified dUTP, dGTP, dCTP 3
   1.2. Synthesis of sulfur modified 7-iodo-2'-deoxy-7-deazaadenosine and 7-iodo-2'-deoxy-7-deazaadenosine 5'-*O*-triphosphates 7
2. Biochemistry 19
   2.1. PEX – Single incorporation (one modified $dN^{R}TP$) 27
      2.1.1. Single incorporation of $dN^{R}TP$ using 19-mer template – analytical scale 27
      2.1.2. Multiple incorporation (one modified $dN^{R}TP$) – 31-mer template – analytical scale 29
      2.1.3. Multiple incorporation (two modified $dN^{R}TPs$) – 31-mer template – analytical scale 32
      2.1.4. Multiple incorporation (three modified $dN^{R}TPs$) – 31-mer template – analytical scale 34
      2.1.5. Multiple incorporation (four modified $dN^{R}TPs$) – 31-mer template – analytical scale 35
      2.1.6. Multiple incorporation (four modified $dN^{R}TPs$) – different template lengths – analytical scale 36
      2.1.7. Multiple incorporation (four modified $dN^{R}TPs$) – mixed PEX using protein-like and lipophilic $dN^{R}TPs$ - 31-mer template - analytical scale 38
      2.1.8. Multiple incorporation (four modified $dN^{R}TPs$) – mixed PEX using $dC^{Im}TP$, $dA^{PSH}TP$ and other two $dN^{R}TPs$ ($dU^{EPh}TP$, $dG^{AiPr}TP$) (two template lengths – analytical scale) 39
   2.2. General procedure for preparation of ONs for MALDI-TOF 40
   2.3. MALDI-TOF measurments 41
   2.4. PCR 41
      2.4.1. PCR – Single incorporation (one modified $dN^{R}TP$) 41
      2.4.2. PCR – Multiple incorporation (two modified $dN^{R}TP$) 43
      2.4.3. PCR – Multiple incorporation (three modified $dN^{R}TPs$) 45
      2.4.4. PCR – Multiple incorporation (four modified $dN^{R}TPs$) 46
   2.5. aPCR (four modified $dN^{R}TPs$) 47
   2.6. rePCR 49
   2.7. Sanger sequencing 50
   2.8. Redox study of $98PCR\_A^{PSH}$ 53
3. Copies of MALDI-TOF mass spectra 54
4. Copies of NMR spectra 62
5. References 83

1. Experimental section – organic chemistry

General remarks

All reagents and solvents were purchased from the commercial suppliers and used as received. Unless otherwise stated, all the reactions were performed under a positive atmosphere of argon by standard syringe, cannula and septa techniques. The reactions were monitored by thin-layer chromatography (TLC) on Merck silica gel 60 F254 plates and visualized by UV (254 nm) and by solution of 4-anisaldehyde in ethanol with 10% of sulfuric acid or by solution of potassium permanganate and potassium carbonate in water with 10% of sodium hydroxide. Purifications were performed using manual column chromatography using silica gel (40–63 μm). Separations of nucleoside triphosphates were performed using HPLC (Waters modular HPLC system) on a column packed with 5 μm C18 reversed phase (Phenomenex, Kinetex EVO C18) or on 50 μm anion exchange resin (Thermo Fisher Scientific, POROS 50 HQ). NMR spectra were measured on Bruker Avance III 400 MHz spectrometer (400.1 MHz for ^{1}H and 100.6 MHz for ^{13}C) and JEOL ECZR 500 MHz spectrometer (1H at 500.2 MHz, ^{13}C at 125.8 MHz and ^{31}P at 202.4 MHz) in $CDCl_3$, DMSO-d6, CD_3OD or D_2O (referenced to the residual solvent signal) at 25 °C. Chemical shifts are given in ppm (δ-scale), coupling constants (J) in Hz. Complete assignment of all NMR signals was performed using a combination of H,H-COSY, H,C-HSQC and H,C-HMBC experiments. Mass spectra and high-resolution mass spectra were measured on a LTQ Orbitrap XL spectrometer (Thermo Fisher Scientific) by ESI ionization technique. All mass spectra were acquired by the MS service at IOCB. Purity of all final compounds was determined by NMR spectra.

Chemicals were of analytical grade. 6-heptynoic acid (**1**), 4-pentyn-1-ol (**2**), histamine hydrochloride, 5-iodo-2'-deoxyuridine (**dU$^{\mathbf{I}}$**) and 5-iodo-2'-deoxycytidine (**dC$^{\mathbf{I}}$**) were purchased from Fluorochem Ltd. Synthesis and characterization data for 7-iodo-2’-deoxy-7-deazaadenosine (**dA$^{\mathbf{I}}$**)[1], 5-iodo-2'-deoxyuridine 5’-*O*-triphosphate (**dU$^{\mathbf{I}}$TP**)[2], 5-iodo-2'-deoxycytidine 5’-*O*-triphosphate (**dC$^{\mathbf{I}}$TP**)[3], 7-iodo-2’-deoxy-7-deazaguanosine 5’-*O*-triphosphate (**dG$^{\mathbf{I}}$TP**)[4], 5-(acetylthio)-pent-1-yne (**4**)[5], 4-(3-bromopropyl)phenyl-B-pin (**5**)[6] were reported previously. The structures of cross-coupling alkynes and boronate ester are displayed in **Scheme S1**. Synthesis of alkyne **3** is described below in Section 1.1.

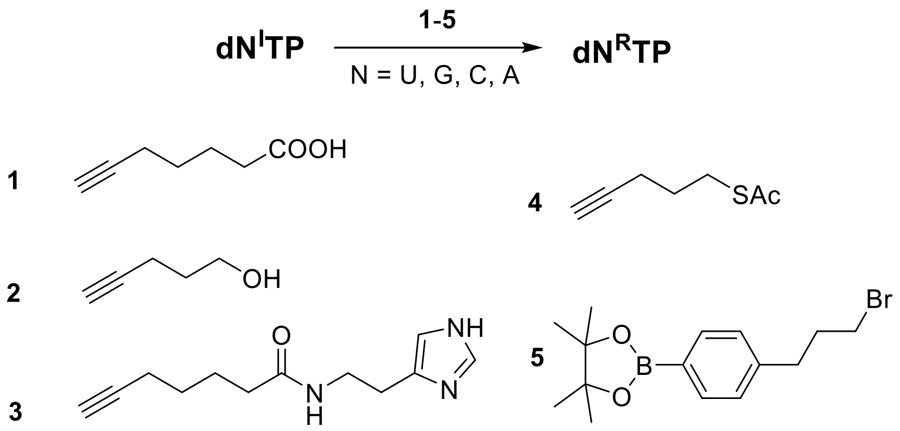

Scheme S1. Structures of compounds **1-5** for cross-coupling reactions

1.1. Synthesis of modified dUTP, dGTP, dCTP

Method A: Halogenated nucleoside triphosphate **dN^{I}TP** (N = U, G, C) (1 equiv.) was dissolved in an argon-purged flask in a mixture of MeCN/H_2O (1:1) 1 mL followed by addition of Et_3N (8 equiv.). $Pd(OAc)_2$ (10 mol %), TPPTS (50 mol %) were dissolved separately in the MeCN/H_2O mixture (1 mL) and added to the nucleoside triphosphate followed by addition of corresponding alkyne (**1-3**) (2 equiv.) The mixture was stirred for 1 hour at 60 °C under argon atmosphere. Solvents were evaporated under vacuum. The product was purified by HPLC with linear gradient of 0.1 M TEAB (triethylammonium bicarbonate) in water to 0.1 M TEAB in H_2O/MeOH (1:1) as eluent in 60 min. Solid product was obtained after lyophilization.

5-(6-Carboxy-hex-1-yn-1-yl)-2'-deoxyuridine 5'-*O*-triphosphate (dU^{COOH}TP)

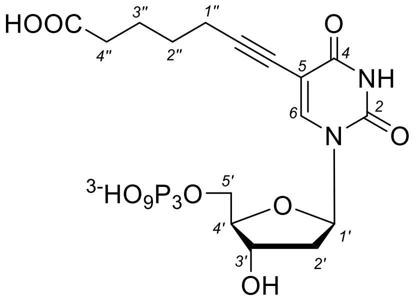

The compound **dU^{COOH}TP** was prepared from dU^{I}TP by **Method A** described above. The product was obtained as a white solid (34 mg, 51 %). ^{1}H NMR (600.1 MHz, D_2O): 1.61 (m, 2H, CH_2-2´´); 1.70 (m, 2H, CH_2-3´´); 2.29 (t, 2H, $J_{4´´,3´´}$ = 7.4 Hz, CH_2-4´´); 2.37 – 2.43 (m, 2H, H-2´); 2.46 (t, 2H, $J_{1´´,2´´}$ = 7.1 Hz, CH_2-1´´); 4.14 - 4.24 (m, 3H; H-4´,5´); 4.62 (m, 1H, H-3´); 6.27 (t, 1H, $J_{1´,2´}$ = 6.9 Hz, H-1´); 8.01 (s, 1H, H-6). ^{13}C NMR (150.9 MHz, D_2O): 20.53 (CH_2-1´´); 26.89 (CH_2-3´´); 29.51 (CH_2-2´´); 38.10 (CH_2-4´´); 40.31 (CH_2-2´); 67.40 (d, $J_{C,P}$ = 5.1 Hz, CH_2-5´); 72.60 (CH-3´); 72.80 (**C**≡C-1´´); 87.40 (d, $J_{C,P}$ = 8.6 Hz, CH-4´); 87.68 (CH-1´); 98.37 (C≡**C**CH_2); 102.33 (C-5); 145.55 (CH-6); 152.56 (C-2); 166.81 (C-4); 184.28 (COO). ^{31}P NMR (202.4 MHz, D_2O): -22.69 (t, 1P, $J_{\beta,\alpha} = J_{\beta,\gamma}$ = 20.1 Hz, P_β); -10.77 (d, 1P, $J_{\alpha,\beta}$ = 20.6 Hz, P_α); -10.22 (d, 1P, $J_{\gamma,\beta}$ = 19.7 Hz, P_γ). HR/MS (ESI-) for $C_{16}H_{22}O_{16}N_2P_3$ 591.01877 $[M-H]^-$ calculated, found 591.01856 $[M-H]^-$

7-(5-Hydroxy-pent-1-yn-1-yl)-2'-deoxy-7-deazaguanosine 5'-*O*-triphosphate (dG^{OH}TP)

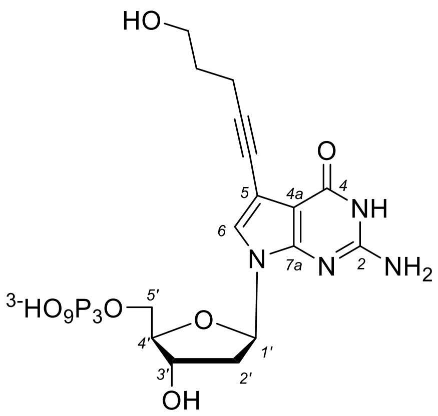

The compound **dG^{OH}TP** was prepared from dG^{I}TP by **Method A** described above. The product was obtained as a brown solid (24 mg, 48 %). ^{1}H NMR (600.1 MHz, D_2O): 1.84 (pent, 2H, $J_{CH2,CH2}$ = 6.7 Hz, $CH_2C\mathbf{H_2}CH_2OH$); 2.39 (ddd, 1H, J_{gem} = 14.0 Hz, $J_{2´a,1´}$ = 6.2 Hz, $J_{2´a,3´}$ = 3.1 Hz, H-2´a); 2.53 (t, 2H, $J_{CH2,CH2}$ = 7.1 Hz, $C\mathbf{H_2}CH_2CH_2OH$); 2.65 (ddd, 1H, J_{gem} = 14.0 Hz, $J_{2´b,1´}$ = 8.2 Hz, $J_{2´b,3´}$ = 6.2 Hz, H-2´b); 3.77 (t, 2H, $J_{CH2,CH2}$ = 6.4 Hz, $CH_2CH_2C\mathbf{H_2}OH$); 4.12 (ddd, 1H, J_{gem} = 11.2 Hz, $J_{5´a,P}$ = 5.5 Hz, $J_{5´a,4´}$ = 4.5 Hz, H-5´a); 4.17 (ddd, 1H, J_{gem} = 11.2 Hz, $J_{5´b,P}$ = 6.4 Hz, $J_{5´b,4´}$ = 4.2 Hz, H-5´b); 4.20 (m, 1H, H-4´); 4.72 (dt, 1H, $J_{3´,2´b}$ = 6.1 Hz, $J_{3´,2´a} = J_{3´,4´}$ = 3.0 Hz, H-3´); 6.41 (dd, 1H, $J_{1´,2´b}$ = 8.2 Hz, $J_{1´,2´a}$ = 6.2 Hz, H-1´); 7.28 (s, 1H, H-6). ^{13}C NMR (150.9 MHz, D_2O): 17.44 (**C**$H_2CH_2CH_2OH$); 32.25 (CH_2**C**H_2CH_2OH); 40.21 (CH_2-2´); 62.55 (CH_2CH_2**C**H_2OH); 67.66 (d, $J_{C,P}$ = 5.4 Hz, CH_2-5´); 73.34 (CH-3´); 75.28 (**C**≡CCH_2); 85.03 (CH-1´); 87.08 (d, $J_{C,P}$ =

9.0 Hz, CH-4′); 94.02 (C≡**C**CH_2); 101.61 (C-5); 102.12 (C-4a); 124.72 (CH-6); 152.71 (C-7a); 155.21 (C-2); 162.90 (C-4). ^{31}P NMR (202.4 MHz, D_2O): -22.61 (t, 1P, $J_{\beta,\alpha} = J_{\beta,\gamma}$ = 19.7 Hz, P_β); -10.64 (d, 1P, $J_{\alpha,\beta}$ = 19.9 Hz, P_α); -10.04 (d, 1P, $J_{\gamma,\beta}$ = 19.4 Hz, P_γ). HR/MS (ESI-) for C_{16} H_{22} O_{14} N_4 P_3 587.03508 $[M-H]^-$ calculated, found 587.03477 $[M-H]^-$

***N*-(2-(Imidazol-4-yl)ethyl)hept-6-ynamide (3)**

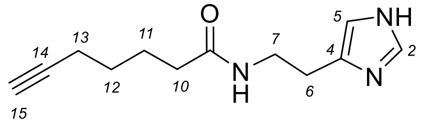

6-Heptynoic acid (45 mg, 0.357 mmol) was dissolved in dry THF (2.5 mL), the solution was cooled with ice. Addition of *N*-methylmorpholine (118 µL, 1.070 mmol) and isobutyl chlorformate (46.3 µL, 0.357 mmol) followed and the mixture was stirred for 10 minutes. After that, histamine hydrochloride (65.7 mg, 0.357 mmol) was added as a suspension in DMF (1 mL). The mixture was stirred overnight at room temperature. Upon evaporation of the solvent, the mixture was purified using HPFC on silica gel (dichloromethane/methanol as eluent, 0 → 10 % of methanol, using iodine staining). The product **3** was isolated as a white solid (66.2 mg, 73 %). ^{13}C NMR (125.7 MHz, CD_3OD): 18.73 (CH_2-13); 25.82 (CH_2-6); 25.98 (CH_2-11); 29.11 (CH_2-12); 36.41 (CH_2-10); 38.86 (CH_2-7); 69.82 (CH-15); 84.59 (C-14); 117.60 (CH-5); 132.89 (C-4); 134.85 (CH-2); 176.11 (CO-9).^{1}H NMR (500 MHz, CD_3OD): 1.46 (m, 2H, H-12); 1.67 (m, 2H, H-11); 2.17 (td, 2H, $J_{13,12}$ = 7.0 Hz, $J_{13,15}$ = 2.7 Hz, H-13); 2.19 (t, 2H, $J_{10,11}$ = 7.5 Hz, H-10); 2.22 (t, 1H, $J_{15,13}$ = 2.7 Hz, H-15); 2.92 (td, 2H, $J_{6,7}$ = 6.8 Hz, $J_{6,5}$ = 0.9 Hz, H-6); 3.50 (t, 2H, $J_{7,6}$ = 6.8 Hz, H-7); 7.36 (q, 1H, $J_{5,2}$ = $J_{5,6}$ = 1.1 Hz, H-5); 8.82 (d, 1H, $J_{2,5}$ = 1.4 Hz, H-2). HR/MS (ESI-) $C_{12}H_{16}ON_3$ 218.12989 $[M-H]^-$ calculated, found 218.12986 $[M-H]^-$

5-(7-((2-(Imidazol-4-yl)ethyl)amino)-7-oxohept-1-yn-1-yl)-2'-deoxycytidine 5'-*O*-triphosphate (dC^{Im}TP)

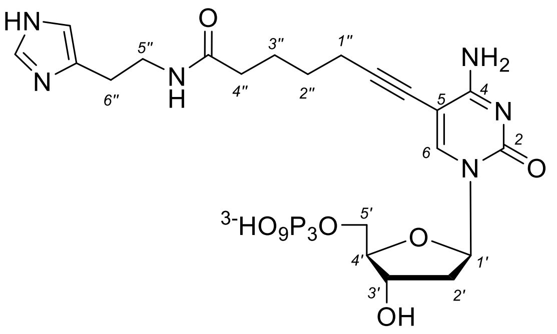

Alkyne **3** (18.5 mg, 0.072 mmol) was added to the mixture of **dC^{I}TP** (48 mg, 0.048 mmol), CuI (1.8 mg, 0.010 mmol). The mixture of starting material was dissolved in a mixture of water and acetonitrile (1:1, 2 mL). Catalyst was prepared separately from $Pd(OAc)_2$ (1.1 mg, 0.005 mmol) and TPPTS (13.6 mg, 0.024 mmol) and added into the reaction mixture followed by addition of Et_3N (40 µL, 0.385 mmol). The reaction mixture was stirred at 60 °C for 1 hour and further proceeded as described above by **Method A**. The product was obtained as a white solid (24 mg, 48 %)

^{1}H NMR (500.0 MHz, D_2O): 1.48 (m, 2H, CH_2-2´´); 1.66 (m, 2H, CH_2-3´´); 2.26 (t, 2H, $J_{4'',3''}$ = 7.2 Hz, CH_2-4´´); 2.29 (dt, 1H, J_{gem} = 14.2 Hz, $J_{2'a,1'} = J_{2'a,3'}$ = 6.7 Hz, H-2´a); 2.43 (t, 2H, $J_{1'',2''}$ = 7.0 Hz, CH_2-1´´); 2.44 (ddd, 1H, J_{gem} = 14.1 Hz, $J_{2'b,1'}$ = 6.4 Hz, $J_{2'b,3'}$ = 3.9 Hz, H-2´b); 2.95 (t, 2H, $J_{6'',5''}$ = 6.5 Hz, CH_2-6´´); 3.52 (t, 2H, $J_{5'',6''}$ = 6.5 Hz, CH_2-5´´); 4.18 - 4.29 (m, 3H; H-4´,5´); 4.63 (dt, 1H, $J_{3',2'a}$ = 6.3 Hz, $J_{3',2'b} = J_{3',4'}$ = 3.7 Hz, H-3´); 6.28 (t, 1H, $J_{1',2'}$ = 6.7 Hz, H-1´); 7.25 (d, 1H, $J_{CH,CH}$ = 1.0 Hz, NCHC); 8.09 (s, 1H, H-6); 8.53 (d, 1H, $J_{CH,CH}$ = 1.0 Hz, NCHN). ^{13}C NMR (125.7 MHz, D_2O): 20.40 (CH_2-1´´); 26.18 (CH_2-6´´); 26.60 (CH_2-3´´); 28.86 (CH_2-2´´); 37.19 (CH_2-4´´); 39.84 (CH_2-5´´); 41.37 (CH_2-2´); 67.14 (d, $J_{C,P}$ = 5.7 Hz, CH_2-5´); 72.32 (CH-3´); 72.57 (**C**≡C-1´´); 87.53 (d, $J_{C,P}$ = 9.0 Hz, CH-4´); 88.08 (CH-1´); 95.37 (C-5); 99.66 (C≡**C**CH_2); 118.34 (N**C**HC); 133.02 (NCH**C**); 135.34 (NCHN); 145.62 (CH-6); 158.03 (C-2); 167.18 (C-4); 178.86 (NHCO). ^{31}P NMR (202.4 MHz, D_2O): -22.27 (t, 1P, $J_{\beta,\alpha} = J_{\beta,\gamma}$ = 20.1 Hz, P_β); -10.71 (d, 1P, $J_{\alpha,\beta}$ = 20.0 Hz, P_α); -8.70 (d, 1P, $J_{\gamma,\beta}$ = 20.2 Hz, P_γ). HR/MS (ESI-) for $C_{21}H_{30}O_{14}N_6P_3$ 683.10383 $[M-H]^-$ calculated, found 683.10307 $[M-H]^-$

1.2.Synthesis of sulfur modified 7-iodo-2’-deoxy-7-deazaadenosine and 7-iodo-2’-deoxy-7-deazaadenosine 5’-*O*-triphosphates

Method B: The triphosphorylation was usually performed from 100 mg of starting nucleoside, which was in advance dried and then dissolved in $PO(OMe)_3$ (approx. 1 mL) in a microwave vial under argon. After cooling down to 0 °C, $POCl_3$ (1.2 equiv.) was added. The reaction mixture was stirred for 2 hours. Then, pyrophosphate $(Bu_3NH)_2H_2P_2O_7$ (5 equiv.) dissolved in dry MeCN (1 mL) was added together with Bu_3N (4 equiv., cooled to 0 °C) into the reaction mixture. After that, the reaction mixture was stirred at 0 °C for additional 1 hour. The reaction was then treated with 2 M TEAB. After partial evaporation of the solvent, the product was purified by HPLC with linear gradient of 0.1 M TEAB (triethylammonium bicarbonate) in water to 0.1 M TEAB in H_2O/MeOH (1:1) as eluent in 60 min. Solid product was obtained after lyophilization.

7-(6-(Acetylthio)pent-1-yn-1-yl)-2’-deoxy-7-deazaadenosine (dA^{ESAc})

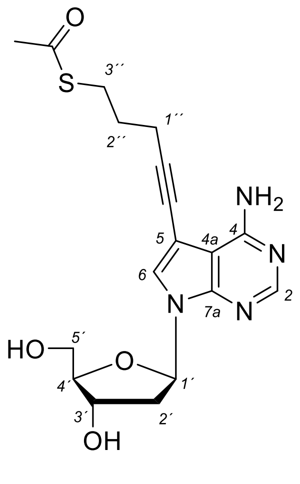

Compound **dA^{ESAc}** was prepared from 7-iodo-2’-deoxy-7-deazaadenosine (**dA^{I}**, 100 mg, 0.27 mmol) was mixed with $Pd(PPh_3)_4$ (10 mol. %) and CuI (20 mol. %). Alkyne **4** (1.2 equiv.) was added, and the mixture was dissolved in DMF. Et_3N (2.5 equiv.) was added, and the reaction mixture was stirred overnight under argon. After evaporation of the solvent, HPFC (dichloromethane and methanol 0 → 10 % of methanol) pure product was obtained as an off-white solid (51 mg, 49 %).

^{1}H NMR (500 MHz, CD_3OD): 1.86 (pent, 2H, $J_{2'',1''} = J_{2'',3''}$ = 7.0 Hz, H-2´´); 2.31 (ddd, 1H, J_{gem} = 13.4 Hz, $J_{2'a,1'}$ = 6.0 Hz, $J_{2'a,3'}$ = 2.7 Hz, H-2´a); 2.33 (s, 3H, CH_3CO); 2.55 (t, 2H, $J_{1'',2''}$ = 6.9 Hz, H-1´´); 2.62 (ddd, 1H, J_{gem} = 13.4 Hz, $J_{2'b,1'}$ = 8.1 Hz, $J_{2'b,3'}$ = 5.9 Hz, H-2´b); 3.04 (bt, 2H, $J_{3'',2''}$ = 7.1 Hz, H-3´´); 3.72 (dd, 1H, J_{gem} = 12.1 Hz, $J_{5'a,4'}$ = 3.7 Hz, H-5´a); 3.79 (dd, 1H, J_{gem} = 12.1 Hz, $J_{5'b,4'}$ = 3.3 Hz, H-5´b); 4.00 (q, 1H, $J_{4',5'} = J_{4',3'}$ = 3.3 Hz, H-4´); 4.51 (dt, 1H, $J_{3',2'b}$ = 5.9 Hz, $J_{3',2'a} = J_{3',4'}$ = 2.8 Hz, H-3´); 6.47 (dd, 1H, $J_{1',2'b}$ = 8.1 Hz, $J_{1',2'a}$ = 6.0 Hz, H-1´); 7.53 (s, 1H, H-6); 8.08 (s, 1H, H-2). ^{13}C NMR (125.7 MHz, CD_3OD): 19.24 (CH_2-1´´); 28.95 (CH_2-3´´); 29.93 (CH_2-2´´); 30.53 (**C**H_3CO); 41.51 (CH_2-2´); 63.64 (CH_2-5´); 73.00 (CH-3´); 74.75 (**C**≡C-1´´); 86.56 (CH-1´); 89.15 (CH-4´); 92.42 (C≡**C**-1´´); 97.73 (C-5); 104.65 (C-4a); 127.62 (CH-6); 149.73 (C-7a); 153.04 (CH-2); 159.20 (C-4); 197.47 (CH_3**C**O). HR/MS (ESI+) for $C_{18}H_{23}O_4N_4S$ 391.14345 $[M+H]^+$ calculated, found 391.14370 $[M+H]^+$

7-(6-(Acetylthio)pent-1-yn-1-yl)-2'-deoxy-7-deazaadenosine 5'-*O*-triphosphate (dA^{ESAc}TP)

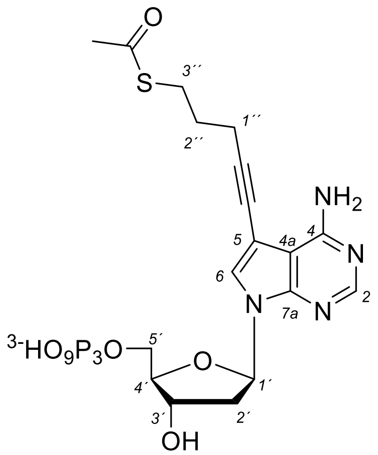

Compound **dA^{ESAc}TP** was prepared from corresponding nucleoside **dA^{ESAc}** according to the general **Method B**. Product **dA^{ESAc}TP** was obtained as a white solid (77 mg, 32 % yield).

^{1}H NMR (600.1 MHz, D_2O): 1.91 (pent, 2H, $J_{2'',1''} = J_{2'',3''}$ = 7.0 Hz, H-2´´); 2.39 (s, 3H, CH_3CO); 2.48 (ddd, 1H, J_{gem} = 13.9 Hz, $J_{2'a,1'}$ = 6.2 Hz, $J_{2'a,3'}$ = 3.2 Hz, H-2´a); 2.58 (t, 2H, $J_{1'',2''}$ = 6.9 Hz, H-1´´); 2.68 (ddd, 1H, J_{gem} = 13.9 Hz, $J_{2'b,1'}$ = 8.1 Hz, $J_{2'b,3'}$ = 6.3 Hz, H-2´b); 3.06 (bt, 2H, $J_{3'',2''}$ = 7.1 Hz, H-3´´); 4.12 (ddd, 1H, J_{gem} = 11.3 Hz, $J_{5'a,P}$ = 5.5 Hz, $J_{5'a,4'}$ = 4.2 Hz, H-5´a); 4.18 (ddd, 1H, J_{gem} = 11.3 Hz, $J_{5'b,P}$ = 6.4 Hz, $J_{5'b,4'}$ = 4.2 Hz, H-5´b); 4.24 (td, 1H, $J_{4',5'a} = J_{4',5'b}$ = 4.2 Hz, $J_{4',3'}$ = 2.9 Hz, $J_{4',P}$ = 1.3 Hz, H-4´); 4.75 (m, 1H, H-3´); 6.59 (dd, 1H, $J_{1',2'b}$ = 8.1 Hz, $J_{1',2'a}$ = 6.1

Hz, H-1´); 7.63 (s, 1H, H-6); 8.13 (s, 1H, H-2). ^{13}C NMR (150.7 MHz, D_2O): 19.92 (CH_2-1´´); 29.63 (CH_2-2´´); 30.02 (CH_2-3´´); 32.04 (**C**H_3CO); 40.51 (CH_2-2´); 67.58 (d, $J_{C,P}$ =5.8 Hz, CH_2-5´); 73.20 (CH-3´); 75.20 (**C**≡C-1´´); 84.91 (CH-1´); 87.22 (d, $J_{C,P}$ = 9.0 Hz, CH-4´); 94.98 (C≡**C**-1´´); 99.11 (C-5); 104.92 (C-4a); 127.54 (CH-6); 150.47 (C-7a); 154.00 (CH-2); 159.31 (C-4); 203.74 (CH_3**C**O). ^{31}P NMR (202.4 MHz, D_2O): -22.43 (t, 1P, $J_{\beta,\alpha} = J_{\beta,\gamma}$ = 20.2 Hz, P_β); -10.61 (d, 1P, $J_{\alpha,\beta}$ = 20.6 Hz, P_α); -9.16 (d, 1P, $J_{\gamma,\beta}$ = 19.9 Hz, P_γ). HR/MS (ESI-) for $C_{18}H_{24}O_{13}N_4P_3S$ 629.02789 $[M-H]^-$ calculated, found 629.02768 $[M-H]^-$.

7-(Dihydrothiophen-2(3H)-ylidene)-2'-deoxy-7-deazaadenosine 5'-*O*-triphosphate (dA^{THT}TP)

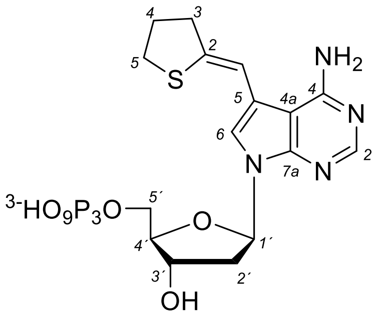

dA^{ESAc}TP (34 mg) was dissolved in aq. ammonia (25 %, 2 mL). After stirring for 1.5 hours, the solvent was evaporated, and product **dA^{THT}TP** was purified by HPLC with linear gradient of 0.1 M TEAB (triethylammonium bicarbonate) in water to 0.1 M TEAB in H_2O/MeOH (1:1) as eluent in 60 min. Solid product was obtained as a white solid (11 mg, 35 % yield).

^1H NMR (500.0 MHz, D_2O): 2.02 – 2.13 (m, 2H, CH_2C**H₂**CH_2S); 2.48 (ddd, 1H, J_{gem} = 14.0 Hz, $J_{2´a,1´}$ = 6.2 Hz, $J_{2´a,3´}$ = 3.2 Hz, H-2´a); 2.61 (ddd, 1H, J_{gem} = 14.0 Hz, $J_{2´b,1´}$ = 7.9 Hz, $J_{2´b,3´}$ = 6.2 Hz, H-2´b); 2.74 – 2.87 (m, 2H, C**H_2**CH_2CH_2S); 3.26 (t, 2H, $J_{CH2,CH2}$ = 6.4 Hz, CH_2CH_2C**H_2**S); 4.14 - 4.25 (m, 2H, H-5´); 4.28 (btd, 1H, $J_{4´,5´}$ = 4.6 Hz, $J_{4´,3´}$ = 3.1 Hz, H-4´); 4.71 (dt, 1H, $J_{3´,2´b}$ = 6.1 Hz, $J_{3´,2´a} = J_{3´,4´}$ = 3.1 Hz, H-3´); 6.35 (bs, 1H, CH=C); 6.55 (dd, 1H, $J_{1´,2´b}$ = 7.9 Hz, $J_{1´,2´a}$ = 6.2 Hz, H-1´); 7.34 (s, 1H, H-6); 8.15 (s, 1H, H-2). ^{13}C NMR (125.7 MHz, D_2O): 30.71 (CH_2**C**H_2CH_2S); 37.39 (CH_2CH_2**C**H_2S);); 40.63 (**C**$H_2CH_2CH_2$S); 40.88 (CH_2-2´); 67.90 (d, $J_{C,P}$ = 5.6 Hz, CH_2-5´); 73.25 (CH-3´); 85.22 (CH-1´); 87.13 (d, $J_{C,P}$ = 8.6 Hz, CH-4´); 102.55 (C-4a); 107.43 (**C**H=C); 117.91 (C-5); 121.37 (CH-6); 148.89 (CH=**C**); 149.20 (C-7a); 156.33 (C-4). ^{31}P

NMR (202.4 MHz, D_2O): -22.35 (t, 1P, $J_{\beta,\alpha} = J_{\beta,\gamma}$ = 19.8 Hz, P_β); -10.56 (d, 1P, $J_{\alpha,\beta}$ = 19.7 Hz, P_α); -9.57 (d, 1P, $J_{\gamma,\beta}$ = 19.8 Hz, P_γ). HR/MS (ESI-) for $C_{16}H_{22}O_{12}N_4P_3S$ 587.01733 $[M-H]^-$ calculated, found 587.01692 $[M-H]^-$

7-(5-Sulfanylpentyl)-2'-deoxy-7-deazaadenosine 5'-*O*-triphosphate (dA^{ESH}TP)

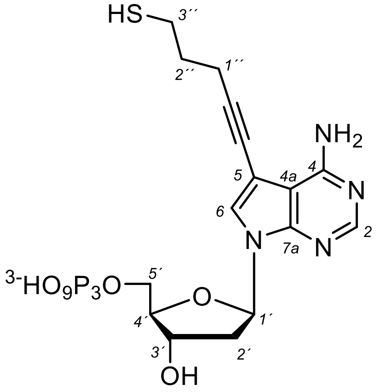

dA^{ESAc}TP (34 mg) was dissolved in aq. ammonia (25 %, 2 mL). After stirring for 1.5 hours, the solvent was evaporated, and product **dA^{ESH}TP** was purified by HPLC with linear gradient of 0.1 M TEAB (triethylammonium bicarbonate) in water to 0.1 M TEAB in H_2O/MeOH (1:1) as eluent in 60 min. Solid product was obtained as a white solid (2 mg, 6 % yield).

^{1}H NMR (500.0 MHz, D_2O): 2.08 (m, 2H, $CH_2C\mathbf{H_2}CH_2SH$); 2.45 (ddd, 1H, J_{gem} = 13.9 Hz, $J_{2'a,1'}$ = 6.4 Hz, $J_{2'a,3'}$ = 3.9 Hz, H-2´a); 2.51 (ddd, 1H, J_{gem} = 13.9 Hz, $J_{2'b,1'}$ = 7.3 Hz, $J_{2'b,3'}$ = 6.3 Hz, H-2´b); 2.69 (m, 2H, $C\mathbf{H_2}CH_2CH_2SH$); 2.97 (m, 2H, $CH_2CH_2C\mathbf{H_2}SH$); 4.19 – 4.30 (m, 3H, H-5´,4´); 4.67 (m, 1H, H-3´); 6.39 (bt, 1H, $J_{1',2'b} = J_{1',2'a}$ = 6.8 Hz, H-1´); 7.56 (s, 1H, H-6); 8.02 (s, 1H, H-2). ^{13}C NMR (125.7 MHz, D_2O): 19.93 ($\mathbf{C}H_2CH_2CH_2SH$); 29.66 ($CH_2\mathbf{C}H_2CH_2SH$); 40.23 ($CH_2CH_2\mathbf{C}H_2SH$); 41.38 (CH_2-2´); 67.61 (d, $J_{C,P}$ = 5.7 Hz, CH_2-5´); 72.81 (CH-3´); 74.07 ($\mathbf{C}{\equiv}CCH_2$); 85.14 (CH-1´); 87.14 (d, $J_{C,P}$ = 8.8 Hz, CH-4´); 96.23 ($C{\equiv}\mathbf{C}CH_2$); 100.24 (C-5); 103.71 (C-4a); 128.48 (CH-6); 148.32 (C-7a); 149.18 (CH-2); 155.33 (C-4). ^{31}P NMR (202.4 MHz, D_2O): -22.37 (t, 1P, $J_{\beta,\alpha} = J_{\beta,\gamma}$ = 19.7 Hz, P_β); -10.54 (d, 1P, $J_{\alpha,\beta}$ = 19.9 Hz, P_α); -9.79 (d, 1P, $J_{\gamma,\beta}$ = 19.6 Hz, P_γ). HR/MS (MALDI) for $C_{16}H_{24}O_{12}N_4P_3S$ 589.0319 $[M+H]^+$ calculated, found 589.03341 $[M+H]^+$

7-(Dihydrothiophen-2(3*H*)-ylidene)-2'-deoxy-7-deazaadenosine (dA^{THT})

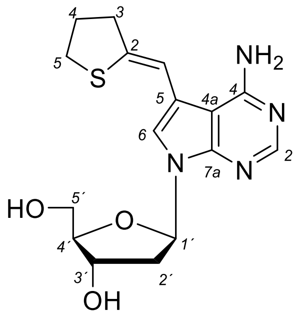

Compound **dA^{THT}** was prepared from **dA^{ESAc}**. The starting material (50 mg) was dissolved in methanol (9 mL) and aq. ammonia (25 %, 1 mL). After stirring for 2 hours, the solvent was evaporated and product **dA^{THT}** was isolated using HPFC (dichloromethane/methanol, 0 → 15 % of methanol) as a sticky yellow solid (37 mg, 74 % yield).

^{1}H NMR (500.0 MHz, CD_3OD): 2.01 – 2.10 (m, 2H, H-4-C_4H_6S); 2.34 (ddd, 1H, J_{gem} = 13.5 Hz, $J_{2'a,1'}$ = 6.1 Hz, $J_{2'a,3'}$ = 3.0 Hz, H-2´a); 2.65 (ddd, 1H, J_{gem} = 13.5 Hz, $J_{2'b,1'}$ = 7.9 Hz, $J_{2'b,3'}$ = 5.9 Hz, H-2´b); 2.80 – 2.88 (m, 2H, H-3-C_4H_6S); 3.16 – 3.21 (m, 2H, H-5-C_4H_6S); 3.73 (dd, 1H, J_{gem} = 12.0 Hz, $J_{5'a,4'}$ = 3.9 Hz, H-5´a); 3.80 (dd, 1H, J_{gem} = 12.0 Hz, $J_{5'b,4'}$ = 3.6 Hz, H-5´b); 4.02 (td, 1H, $J_{4',5'a}$ = $J_{4',5'b}$ = 3.8 Hz, $J_{4',3'}$ = 2.8 Hz, H-4´); 4.53 (dt, 1H, $J_{3',2'b}$ = 5.9 Hz, $J_{3',2'a}$ = $J_{3',4'}$ = 2.9 Hz, H-3´); 6.54 (dd, 1H, $J_{1',2'b}$ = 7.9 Hz, $J_{1',2'a}$ = 6.1 Hz, H-1´); 6.67 (td, 1H, $J_{CH,6}$ = 0.6 Hz, C=CH); 7.42 (s, 1H, H-6); 8.05 (s, 1H, H-2). ^{13}C NMR (125.7 MHz, CD_3OD): 30.03 (CH_2-4-C_4H_6S); 35.97 (CH_2-5-C_4H_6S); 39.81 (CH_2-3-C_4H_6S); 41.31 (CH_2-2´); 63.86 (CH_2-5´); 73.16 (CH-3´); 86.41 (CH-1´); 88.95 (CH-4´); 103.15 (C-4a); 107.72 (C=**C**H); 116.05 (C-5); 121.95 (CH-6); 145.71 (C-2-C_4H_6S); 150.19 (C-7a); 151.30 (CH-2); 158.91 (C-4).HR/MS (ESI+) for $C_{16}H_{21}O_3N_4S$ 349.13289 $[M+H]^+$ calculated, found 349.13280 $[M+H]^+$

7-(5-Sylfanylpentyl)-2'-deoxy-7-deazaadenosine 5'-*O*-triphosphate ($dA^{ASH}TP$)

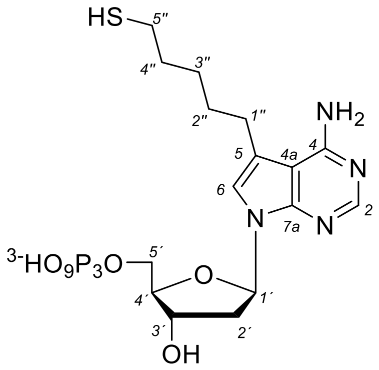

Nucleoside triphosphate **$dA^{ASH}TP$** was prepared from **dA^{ESAc}** through series of reaction. First, **dA^{ESAc}** (120 mg, 0.307 mmol) was dissolved in dry methanol (15 mL) and catalytically hydrogenated using inlet of H_2 (from a balloon) and Pd/C (1 equiv.), that was poured into the mixture. The reaction was stirred at reflux for 8 hours. Then, the catalyst was filtered off on a celite. After evaporation of the solvent and HPFC (dichloromethane/methanol, 0 → 15 % of methanol), crude product was obtained. The dried crude product (60 mg) underwent triphosphorylation according to **Method B** and further deprotection using aq. ammonia (25 %, 5 mL). The product was purified by HPLC with linear gradient of 0.1 M TEAB (triethylammonium bicarbonate) in water to 0.1 M TEAB in H_2O/MeOH (1:1) as eluent in 60 min. The product **$dA^{ASH}TP$** was obtained as a white solid (11 mg, 6 % yield overall)

^{1}H NMR (600.1 MHz, D_2O): 1.40 – 1.50 (m, 2H, H-3´´); 1.55 – 1.70 (m, 4H, H-2´´,4´´); 2.42 (ddd, 1H, J_{gem} = 14.0 Hz, $J_{2'a,1'}$ = 6.1 Hz, $J_{2'a,3'}$ = 3.0 Hz, H-2´a); 2.54 (t, 2H, $J_{5'',4''}$ = 7.1 Hz, H-5´´); 2.66 (ddd, 1H, J_{gem} = 14.0 Hz, $J_{2'b,1'}$ = 8.1 Hz, $J_{2'b,3'}$ = 6.2 Hz, H-2´b); 2.70 (bt, 2H, $J_{1'',2''}$ = 7.4 Hz, H-1´´); 4.12 – 4.22 (m, 2H, H-5´); 4.23 (m, 1H, H-4´); 4.73 (dt, 1H, $J_{3',2'b}$ = 6.1 Hz, $J_{3',2'a}$ = $J_{3',4'}$ = 2.9 Hz, H-3´); 6.59 (dd, 1H, $J_{1',2'b}$ = 8.1 Hz, $J_{1',2'a}$ = 6.1 Hz, H-1´); 7.27 (s, 1H, H-6); 8.13 (s, 1H, H-2). ^{13}C NMR (150.9 MHz, D_2O): 25.70 (CH_2-5´´); 27.46 (CH_2-1´´); 28.82 (CH_2-3´´); 30.85 (CH_2-2´´); 34.73 (CH_2-4´´); 40.48 (CH_2-2´); 67.72 (d, $J_{C,P}$ = 5.4 Hz, CH_2-5´); 73.37 (CH-3´); 84.75 (CH-1´); 87.02 (d, $J_{C,P}$ = 9.0 Hz, CH-4´); 104.09 (C-4a); 120.05 (C-5); 121.60 (CH-6); 150.31 (CH-2); 151.02 (C-7a); 157.34 (C-4). ^{31}P NMR (202.4 MHz, D_2O): -22.41 (t, 1P, $J_{\beta,\alpha} = J_{\beta,\gamma}$ = 19.9 Hz, P_β); -10.62 (d, 1P, $J_{\alpha,\beta}$ = 20.0 Hz, P_α); -9.48 (d, 1P, $J_{\gamma,\beta}$ = 19.7 Hz, P_γ). HR/MS (ESI-) for $C_{16}H_{26}O_{12}N_4P_3S$ 591.04863 $[M-H]^-$ calculated, found 591.04809 $[M-H]^-$

dA^{ASSA}dA

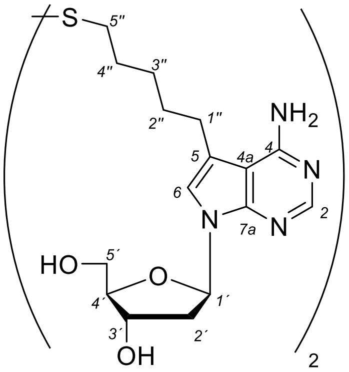

Disulfide **dA^{ASSA}dA** was prepared from corresponding acetyl protected nucleoside **dA^{ESAc}TP** through three subsequent reactions. First, compound **dA^{ESAc}TP** (164 mg, 0.420 mmol) was dissolved in dry methanol (20 mL) and reduced using Pd/C (1 equiv.), that was poured into the mixture. The reaction was stirred at reflux with inlet of H_2 from a balloon for 8 hours. Then, the catalyst was filtered off on a celite. After evaporation of the solvent and HPFC (dichloromethane/methanol, 0 → 15 % of methanol), crude product was obtained. This mixture (82 mg) containing reduced product underwent deprotection after being treated with aq. ammonia in methanol (10 mL, 1 : 9), for 2 hours at room temperature. After evaporation of the solvent, the mixture was re-dissolved in methanol and the inseparable thiol was oxidized using air inlet. The disulfide **dA^{ASSA}dA** was isolated (46 mg, 31 %) using HPFC on silica gel (dichloromethane/methanol, 0 → 20 % of methanol).

^{1}H NMR (500.0 MHz, CD_3OD): 1.46 – 1.58 (m, 4H, H-3´´); 1.64 – 1.80 (m, 8H, H-2´´,4´´); 2.28 (ddd, 2H, J_{gem} = 13.4 Hz, $J_{2'a,1'}$ = 6.0 Hz, $J_{2'a,3'}$ = 2.5 Hz, H-2´a); 2.676 (ddd, 2H, J_{gem} = 13.4 Hz, $J_{2'b,1'}$ = 8.4 Hz, $J_{2'b,3'}$ = 6.0 Hz, H-2´b); 2.684 (t, 4H, $J_{5'',4''}$ = 7.1 Hz, H-5´´); 2.80 (bt, 4H, $J_{1'',2''}$ = 7.2 Hz, H-1´´); 3.73 (dd, 2H, J_{gem} = 12.1 Hz, $J_{5'a,4'}$ = 3.7 Hz, H-5´a); 3.80 (dd, 2H, J_{gem} = 12.1 Hz, $J_{5'b,4'}$ = 3.4 Hz, H-5´b); 4.01 (td, 2H, $J_{4',5'a}$ = $J_{4',5'b}$ = 3.5 Hz, $J_{4',3'}$ = 2.5 Hz, H-4´); 4.52 (dt, 2H, $J_{3',2'b}$ = 5.9 Hz, $J_{3',2'a}$ = $J_{3',4'}$ = 2.5 Hz, H-3´); 6.48 (dd, 2H, $J_{1',2'b}$ = 8.4 Hz, $J_{1',2'a}$ = 6.0 Hz, H-1´); 7.11 (s, 2H, H-6); 8.04 (s, 2H, H-2). ^{13}C NMR (125.7 MHz, CD_3OD): 27.14 (CH_2-1´´); 28.72 (CH_2-3´´); 29.96 (CH_2-4´´); 31.03 (CH_2-2´´); 39.65 (CH_2-5´´); 41.22 (CH_2-2´); 63.83 (CH_2-5´); 73.17 (CH-3´); 86.40 (CH-1´); 88.91 (CH-4´); 104.47 (C-4a); 117.19 (C-5); 121.31 (CH-6); 151.08 (C-7a); 151.77 (CH-2); 159.18 (C-4). HR/MS (ESI+) for $C_{32}H_{47}O_6N_8S_2$ 703.30545 $[M+H]^+$ calculated, found 703.30531 $[M+H]^+$

7-(5-Sulfanylpent-1-yl)-2'-deoxy-7-deazaadenosine (dA^{ASH})

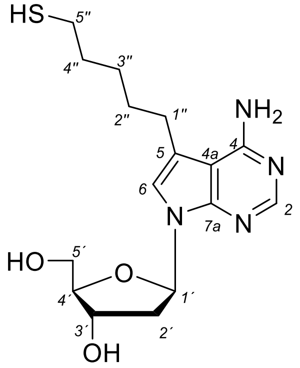

Disulfide **dA^{ASSA}dA** (43.5 mg, 0.062 mmol) was mixed with TCEP · HCl (26.6 mg, 0.093 mmol) and dissolved in a mixture of methanol and water (4 : 1) for 1 hour. After reverse phase HPFC on C18, the product **dA^{ASH}** was obtained as a white solid (22 mg, 50 % yield).

^{1}H NMR (500.0 MHz, CD_3OD): 1.48 – 1.57 (m, 2H, H-3´´); 1.61 – 1.73 (m, 4H, H-2´´,4´´); 2.27 (ddd, 1H, J_{gem} = 13.4 Hz, $J_{2'a,1'}$ = 6.0 Hz, $J_{2'a,3'}$ = 2.5 Hz, H-2´a); 2.51 (t, 2H, $J_{5'',4''}$ = 7.1 Hz, H-5´´); 2.66 (ddd, 1H, J_{gem} = 13.4 Hz, $J_{2'b,1'}$ = 8.4 Hz, $J_{2'b,3'}$ = 6.0 Hz, H-2´b); 2.79 (bt, 2H, $J_{1'',2''}$ = 7.6 Hz, H-1´´); 3.71 (dd, 1H, J_{gem} = 12.1 Hz, $J_{5'a,4'}$ = 3.7 Hz, H-5´a); 3.79 (dd, 1H, J_{gem} = 12.1 Hz, $J_{5'b,4'}$ = 3.4 Hz, H-5´b); 3.99 (td, 1H, $J_{4',5'a}$ = $J_{4',5'b}$ = 3.5 Hz, $J_{4',3'}$ = 2.5 Hz, H-4´); 4.50 (dt, 1H, $J_{3',2'b}$ = 6.0 Hz, $J_{3',2'a}$ = $J_{3',4'}$ = 2.6 Hz, H-3´); 6.47 (dd, 1H, $J_{1',2'b}$ = 8.4 Hz, $J_{1',2'a}$ = 5.9 Hz, H-1´); 7.11 (s, 1H, H-6); 8.03 (s, 1H, H-2). ^{13}C NMR (125.7 MHz, CD_3OD): 24.92 (CH_2-5´´); 27.20 (CH_2-1´´); 28.82 (CH_2-3´´); 30.99 (CH_2-2´´); 35.03 (CH_2-4´´); 41.21 (CH_2-2´); 63.83 (CH_2-5´); 73.16 (CH-3´); 86.40 (CH-1´); 88.90 (CH-4´); 104.45 (C-4a); 117.28 (C-5); 121.25 (CH-6); 151.07 (C-7a); 151.76 (CH-2); 159.20 (C-4). HR/MS (ESI+) for $C_{16}H_{25}O_3N_4S$ $[M+H]^+$ 353.16419 calculated, 353.16409 found $[M+H]^+$

7-(4-(3-(Acetylthio)propyl)phenyl)-2'-deoxy-7-deazaadenosine (dA^{PSAc})

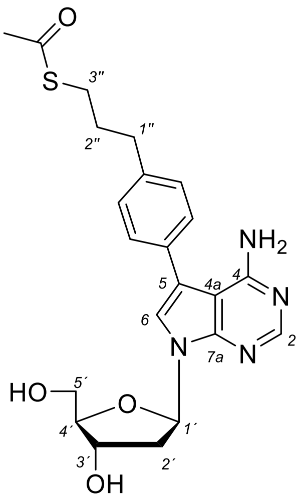

Starting nucleoside **dA^{I}** (104 mg, 0.278 mmol) and boronate **5** (100 mg, 0.308 mmol) were mixed with Pd(PPh_3)$_4$ (36 mg, 0.031 mmol) and sodium carbonate (98 mg, 0.924 mmol). The mixture was dissolved in acetonitrile/water (4 mL, 4:1) and stirred for 45 minutes at 90 °C under argon atmosphere. After evaporation of the solvent and quick column chromatography (dichloromethane/methanol, 10 % of methanol) the crude bromo-intermediate underwent subsequent substitution reaction with potassium thioacetate (31 mg, 0.278 mmol) in acetone (5 mL) at reflux for 3 hours. After that, solvent was evaporated and the product was isolated using HPFC on silica gel (dichloromethane/methanol, 0 → 10 % of methanol). Product **dA^{PSAc}** was obtained as yellow solid (30 mg, 25 %). ^{1}H NMR (500.0 MHz, CD_3OD): 1.89 – 1.96 (m, 2H, H-2´´); 2.32 (s, 3H, CH_3O); 2.35 (ddd, 1H, J_{gem} = 13.4 Hz, $J_{2'a,1'}$ = 6.0 Hz, $J_{2'a,3'}$ = 3.0 Hz, H-2´a); 2.72 (ddd, 1H, J_{gem} = 13.4 Hz, $J_{2'b,1'}$ = 8.3 Hz, $J_{2'b,3'}$ = 6.0 Hz, H-2´b); 2.75 (bt, 2H, $J_{1'',2''}$ = 7.6 Hz, H-1´´); 2.90 (bt, 2H, $J_{3'',2''}$ = 7.3 Hz, H-3´´); 3.73 (dd, 1H, J_{gem} = 12.1 Hz, $J_{5'a,4'}$ = 3.7 Hz, H-5´a); 3.81 (dd, 1H, J_{gem} = 12.1 Hz, $J_{5'b,4'}$ = 3.4 Hz, H-5´b); 4.02 (td, 1H, $J_{4',5'a}$ = $J_{4',5'b}$ = 3.5 Hz, $J_{4',3'}$ = 2.6 Hz, H-4´); 4.54 (dt, 1H, $J_{3',2'b}$ = 5.9 Hz, $J_{3',2'a}$ = $J_{3',4'}$ = 2.6 Hz, H-3´); 6.58 (dd, 1H, $J_{1',2'b}$ = 8.3 Hz, $J_{1',2'a}$ = 6.0 Hz, H-1´); 7.31 (m, 2H, H-*m*-Ph); 7.39 (s, 1H, H-6); 7.42 (m, 2H, H-*o*-Ph); 9.13 (s, 1H, H-2).^{13}C NMR (125.7 MHz, CD_3OD): 29.26 (CH_2-3´´); 30.52 (**C**H_3CO); 32.45 (CH_2-2´´); 35.40 (CH_2-1´´); 41.45 (CH_2-2´); 63.71 (CH_2-5´); 73.10 (CH-3´); 86.51 (CH-1´); 89.06 (CH-4´); 102.87 (C-4a); 118.63 (C-5); 122.44 (CH-6); 130.06 (CH-*o*-Ph); 130.26 (CH-*m*-Ph); 133.37 (C-*i*-Ph); 142.01 (C-*p*-Ph); 151.03 (C-7a); 152.21 (CH-2); 158.92 (C-4); 197.50 (CH_3**CO**). HR/MS (ESI+) for $C_{22}H_{27}O_4N_4S$ 443.17475 $[M+H]^+$ calculated, found 443.17468 $[M+H]^+$

7-(4-(3-(Acetylthio)propyl)phenyl)-2’-deoxy-7-deazaadenosine 5’-*O*-triphosphate (dA^{PSAc}TP)

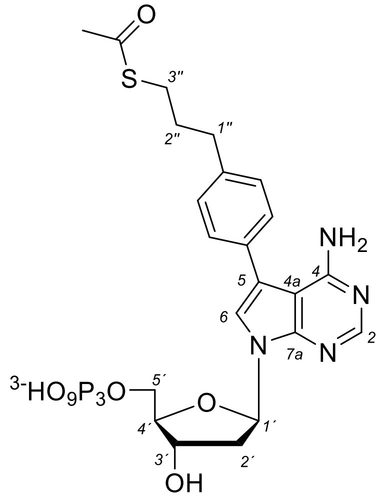

Compound **dA^{PSAc}TP** was prepared using **Method B** from compound **dA^{PSAc}** as a yellow solid (75 mg, 64 %). ^{1}H NMR (600.1 MHz, D_2O): 1.88 (pent, 2H, $J_{2'',3''} = J_{2'',1''} = 7.4$ Hz, H-2´´); 2.37 (s, 3H, CH_3O); 2.47 (ddd, 1H, $J_{gem} = 14.0$ Hz, $J_{2'a,1'} = 6.0$ Hz, $J_{2'a,3'} = 3.0$ Hz, H-2´a); 2.70 (t, 2H, $J_{1'',2''} = 7.5$ Hz, H-1´´); 2.73 (ddd, 1H, $J_{gem} = 14.0$ Hz, $J_{2'b,1'} = 7.9$ Hz, $J_{2'b,3'} = 6.5$ Hz, H-2´b); 2.87 (t, 2H, $J_{3'',2''} = 7.2$ Hz, H-3´´); 4.13 (bdt, 1H, $J_{gem} = 11.4$ Hz, $J_{5'a,P} = J_{5'a,4'} = 5.0$ Hz, H-5´a); 4.18 (ddd, 1H, $J_{gem} = 11.4$ Hz, $J_{5'b,P} = 6.3$ Hz, $J_{5'b,4'} = 4.4$ Hz, H-5´b); 4.24 (bq, 1H, $J_{4',5'a} = J_{4',5'b} = J_{4',3'} = 3.6$ Hz, H-4´); 4.77 (m, 1H, H-3´); 6.65 (dd, 1H, $J_{1',2'b} = 7.9$ Hz, $J_{1',2'a} = 6.2$ Hz, H-1´); 7.28 (m, 2H, H-*m*-Ph); 7.34 (m, 2H,H-*o*-Ph); 7.50 (s, 1H, H-6); 8.16 (s, 1H, H-2). ^{13}C NMR (150.9 MHz, D_2O): 30.34 (CH_2-3´´); 32.08 (**C**H_3CO); 32.26 (CH_2-2´´); 35.65 (CH_2-1´´); 40.40 (CH_2-2´); 67.69 (bd, $J_{C,P} = 5.2$ Hz, CH_2-5´); 73.30 (CH-3´); 84.80 (CH-1´); 87.15 (d, $J_{C,P} = 8.8$ Hz, CH-4´); 102.78 (C-4a); 120.24 (C-5); 122.11 (CH-6); 130.70 (CH-*o*-Ph); 131.34 (CH-*m*-Ph); 133.01 (C-*i*-Ph); 143.03 (C-*p*-Ph); 151.70 (C-7a); 152.69 (CH-2); 158.67 (C-4); 203.89 (CH_3**CO**). ^{31}P NMR (202.4 MHz, D_2O): -22.40 (t, 1P, $J_{\beta,\alpha} = J_{\beta,\gamma} = 20.2$ Hz, P_β); -10.64 (d, 1P, $J_{\alpha,\beta} = 20.1$ Hz, P_α); -8.81 (d, 1P, $J_{\gamma,\beta} = 20.4$ Hz, P_γ). HR/MS (ESI-) for $C_{22}H_{28}O_{13}N_4P_3S$ 681.05919 $[M-H]^-$ calculated, found 681.05874 $[M-H]^-$

7-(4-(3-Sulfanylpropyl)phenyl)-2'-deoxy-7-deazaadenosine 5'-*O*-triphosphate (dA^{PSH}TP)

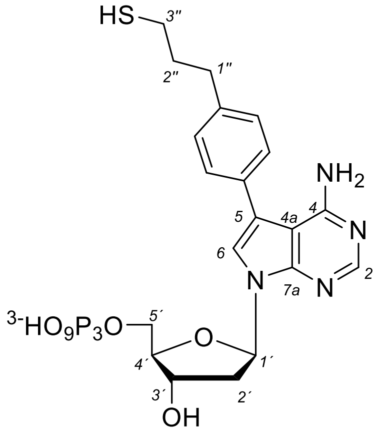

Compound **dA^{PSAc}TP** (50 mg, 0.06 mmol) was dissolved in water (5 mL) and aq. ammonia was added subsequently (25 %, 0.5 mL). The mixture was stirred for 1.5 hours at room temperature. The product **dA^{PSH}TP** was purified by HPLC with linear gradient of 0.1 M TEAB (triethylammonium bicarbonate) in water to 0.1 M TEAB in H_2O/MeOH (1:1) as eluent in 60 min and obtained as a white solid (31 mg, 67 %). ^{1}H NMR (600.1 MHz, D_2O): 1.94 (m, 2H, H-2´´); 2.45 (ddd, 1H, J_{gem} = 14.0 Hz, $J_{2'a,1'}$ = 6.2 Hz, $J_{2'a,3'}$ = 3.1 Hz, H-2´a); 2.57 (t, 2H, $J_{3'',2''}$ = 7.2 Hz, H-3´´); 2.73 (ddd, 1H, J_{gem} = 14.0 Hz, $J_{2'b,1'}$ = 8.1 Hz, $J_{2'b,3'}$ = 6.4 Hz, H-2´b); 2.76 (bt, 2H, $J_{1'',2''}$ = 7.6 Hz, H-1´´); 4.12 (ddd, 1H, J_{gem} = 11.3 Hz, $J_{5'a,P}$ = 5.6 Hz, $J_{5'a,4'}$ = 4.5 Hz, H-5´a); 4.18 (ddd, 1H, J_{gem} = 11.3 Hz, $J_{5'b,P}$ = 6.4 Hz, $J_{5'b,4'}$ = 4.3 Hz, H-5´b); 4.24 (m, 1H, H-4´); 4.76 (dt, 1H, $J_{3',2'b}$ = 6.1 Hz, $J_{3',2'a}$ = $J_{3',4'}$ = 3.0 Hz, H-3´); 6.66 (dd, 1H, $J_{1',2'b}$ = 8.2 Hz, $J_{1',2'a}$ = 6.2 Hz, H-1´); 7.33 (m, 2H, H-*m*-Ph); 7.38 (m, 2H, H-*o*-Ph); 7.49 (s, 1H, H-6); 8.16 (s, 1H, H-2). ^{13}C NMR (150.9 MHz, D_2O): 25.23 (CH_2-3´´); 35.32 (CH_2-1´´); 36.74 (CH_2-2´´); 40.31 (CH_2-2´); 67.61 (bd, $J_{C,P}$ = 5.1 Hz, CH_2-5´); 73.25 (CH-3´); 84.77 (CH-1´); 87.10 (d, $J_{C,P}$ = 8.8 Hz, CH-4´); 102.87 (C-4a); 120.22 (C-5); 122.01 (CH-6); 130.76 (CH-*o*-Ph); 131.29 (CH-*m*-Ph); 133.01 (C-*i*-Ph); 143.36 (C-*p*-Ph); 151.73 (C-7a); 152.86 (CH-2); 158.85 (C-4). ^{31}P NMR (202.4 MHz, D_2O): -21.91 (t, 1P, $J_{\beta,\alpha}$ = $J_{\beta,\gamma}$ = 20.0 Hz, P_β); -10.50 (d, 1P, $J_{\alpha,\beta}$ = 19.9 Hz, P_α); -7.81 (d, 1P, $J_{\gamma,\beta}$ = 20.1 Hz, P_γ). HR/MS (ESI-) for $C_{20}H_{26}O_{12}N_4P_3S$ 639.04863 [M-H]$^-$ calculated, found 639.04769 [M-H]$^-$

7-(4-(3-Sulfanylpropyl)phenyl)-2'-deoxy-7-deazaadenosine (dA^{PSH})

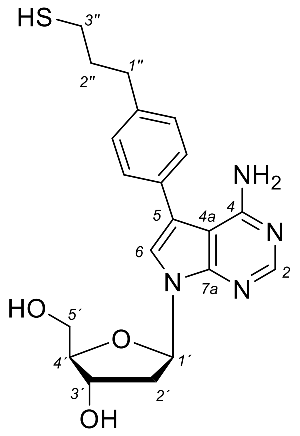

dA^{PSAc} (50 mg, 0.113 mmol) was mixed with DTT (21 mg, 0.124 mmol) and dissolved in methanol (10 mL), upon which aq. ammonia was added (25 %, 1 mL). The mixture was stirred for 2 hours at room temperature under argon. After evaporation of the solvent, the product **dA^{PSH}** was isolated using HPFC on silica gel (dichloromethane/methanol, 0 → 10 % of methanol) as an off-white solid (43 mg, 95 %). ^{1}H NMR (500.0 MHz, CD_3OD): 1.94 (m, 2H, H-2´´); 2.34 (ddd, 1H, J_{gem} = 13.4 Hz, $J_{2'a,1'}$ = 6.0 Hz, $J_{2'a,3'}$ = 2.6 Hz, H-2´a); 2.53 (t, 2H, $J_{3'',2''}$ = 7.1 Hz, H-3´´); 2.71 (ddd, 1H, J_{gem} = 13.4 Hz, $J_{2'b,1'}$ = 8.3 Hz, $J_{2'b,3'}$ = 6.0 Hz, H-2´b); 2.78 (bt, 2H, $J_{1'',2''}$ = 7.6 Hz, H-1´´); 3.73 (dd, 1H, J_{gem} = 12.1 Hz, $J_{5'a,4'}$ = 3.6 Hz, H-5´a); 3.81 (dd, 1H, J_{gem} = 12.1 Hz, $J_{5'b,4'}$ = 3.7 Hz, H-5´b); 4.02 (td, 1H, $J_{4',5'a}$ = $J_{4',5'b}$ = 3.5 Hz, $J_{4',3'}$ = 2.5 Hz, H-4´); 4.54 (dt, 1H, $J_{3',2'b}$ = 5.9 Hz, $J_{3',2'a}$ = $J_{3',4'}$ = 2.6 Hz, H-3´); 6.58 (dd, 1H, $J_{1',2'b}$ = 8.2 Hz, $J_{1',2'a}$ = 6.0 Hz, H-1´); 7.31 (m, 2H, H-*m*-Ph); 7.38 (s, 1H, H-6); 7.40 (m, 2H, H-*o*-Ph); 8.13 (s, 1H, H-2). ^{13}C NMR (125.7 MHz, CD_3OD): 24.37 (CH_2-3´´); 34.97 (CH_2-1´´); 36.82 (CH_2-2´´); 41.46 (CH_2-2´); 63.71 (CH_2-5´); 73.09 (CH-3´); 86.51 (CH-1´); 89.07 (CH-4´); 102.87 (C-4a); 118.62 (C-5); 122.45 (CH-6); 130.00 (CH-*o*-Ph); 130.26 (CH-*m*-Ph); 133.28 (C-*i*-Ph); 142.31 (C-*p*-Ph); 151.04 (C-7a); 152.21 (CH-2); 158.91 (C-4). HR/MS (ESI+) for $C_{20}H_{25}O_3N_4S$ $[M+H]^+$ 401.16419 calculated, 401.16425 found $[M+H]^+$.

2. Biochemistry

General remarks

All polyacrylamide (PAGE) gels were analyzed by fluorescence imaging using Typhoon FLA 9500 (GE Healthcare) or Amersham Typhoon (Cytiva). The MALDI-TOF spectra of modified oligonucleotides were measured on UltrafleXtreme MALDI-TOF/TOF (Bruker) mass spectrometer with 1 kHz smartbeam II laser by MS service at IOCB. UV-Vis spectra were measured at room temperature in a NanoPhotometer N60 (Implen). Synthetic oligonucleotides (primers, templates and biotinylated templates; for sequences see **Table S1**) were purchased from Generi Biotech (Czech Republic) or biomers.net (Germany). Natural nucleoside triphosphates (dATP, dGTP, TTP, dCTP) were purchased from Thermo Fisher Scientific. Pwo DNA polymerase and corresponding polymerase reaction buffer were purchased from Merck Life Science, Vent(exo-) and corresponding polymerase reaction buffer were purchased from NEB, KOD XL DNA polymerase and corresponding buffer were purchased from Merck Life Science. Streptavidin magnetic particles (Roche) were obtained from Merck Life Science, QIAquick® Nucleotide Removal Kit (QIAGEN) was purchased from BioTech (Czech Republic). Sanger sequencing was done by SeqMe (Czech Republic). Milli-Q water was used for all experiments. PAGE stop solution used after PEX reactions contains: 95% [v/v] formamide, 0.5 mM EDTA, 0.025% bromophenol blue and 0.025% [w/v] xylene cyanol, 0.025% [w/v] SDS and Milli-Q water. Samples after PEX reactions were always separated on a 12.5% PAGE (acrylamide/bisacrylamide 19:1, 25% urea) under denaturing conditions in 1X TBE buffer (42 mA, 1h). Samples after PCR were analyzed by a 2% agarose gel (Serva) in 0.5X TBE buffer (120 V, 75 min) using 6X DNA Gel Loading Dye (60 mM EDTA, 10 mM Tris-HCl (pH 7.6), 60% glycerol, 0.03% bromophenol blue, 0.03% xylene cyanol FF, ThermoFisher Scientific). In case of no fluorescent label, the gel contained Gel Red (Biotinum, 10,000X in water). Single-stranded ladder in several dPAGE scans was prepared by mixing of ten natural ONs (20-, 43-, 61-, 77- an 98nt long) labeled at 5'- end with 6-FAM and Cy5.

Unless other specified, **dA$^{\mathbf{PSH}}$TP** and **dA$^{\mathbf{ASH}}$TP** was pretreated with 4 equivalents of TCEP before every reaction to reduce any oxidized disulfide products.

Table S1. List of oligonucleotides used in this study

ON Name	Size (nt)	Sequence in 5’→3’ direction with primer regions underlined
**Prim248short**[a]	15	CATGGGCGGCATGGG
**Oligo 1A**	19	CCCTCCCATGCCGCCCATG
**Oligo 1A**[b]	19	CCCTCCCATGCCGCCCATG
**Oligo 1C**	19	CCCGCCCATGCCGCCCATG
**Oligo 1C**[b]	19	CCCGCCCATGCCGCCCATG
**Oligo 1T**	19	CCCACCCATGCCGCCCATG
**Oligo 1T**[b]	19	CCCACCCATGCCGCCCATG
**Oligo 1G**	19	AAACCCCATGCCGCCCATG
**Oligo 1G**[b]	19	TTTCCCCATGCCGCCCATG
**Prb4basII**	31	CTAGCATGAGCTCAGTCCCATGCCGCCCATG
**Prb4basII**[b]	31	CTAGCATGAGCTCAGTCCCATGCCGCCCATG
**MO43**	43	CATGAGCTCAGTCTAGCATGAGCTCAGTCCCATGCCGCCCATG
**MO61**	61	GACATCATGAGAGACATCGCCTAGCATGAGCTCAGTAAGGAATACAGGTATTTTGTCCTTG
**FVL-A**	98	GACATCATGAGAGACATCGCCTCTGGGCTAATAGGACTACTTCTAATCTGTAAGAGCAGATCCCTGGACAGGCAAGGAATACAGGTATTTTGTCCTTG
**FVL-A-sC3**	98	GACATCATGAGAGACATCGCCTCTGGGCTAATAGGACTACTTCTAATCTGTAAGAGCAGATCCCTGGACAGGCAAGGAATACAGGTATTTTGTCCTTG
**MO120**	120	GACATCATGAGAGACATCGCCTAGCATGAGCTCAGTCTAGCATGAGCTCAGTCTAGCATGAGCTCAGTCTAGCATGAGCTCAGTCTAGCATGAGCAAGGAATACAGGTATTTTGTCCTTG
**LT25TH** [a]	25	CAAGGACAAAATACCTGTATTCCTT
**L20** [c]	20	GACATCATGAGAGACATCGC
**Flank_LT25TH** [a]	45	CATTCGGCTGCTCTTGATTTCAAGGACAAAATACCTGTATTCCTT

**Flank** [a]	20	CATTCGGCTGCTCTTGATTT
**Flank** [c]	20	CATTCGGCTGCTCTTGATTT
**Flank**	20	CATTCGGCTGCTCTTGATTT
**Flank Seq+** [d]	20+	CATTCGGCTGCTCTTGATTT
**L20** [a]	20	GACATCATGAGAGACATCGC
**L20**	20	GACATCATGAGAGACATCGC
**L20 Seq+** [d]	20+	GACATCATGAGAGACATCGC

[a] 5'-(6-FAM); [b] 5'-biotinylated; [c] 5'-(Cy5); sC3 = three carbon spacer at 3' end; [d] extended at 5′-end with unknown sequence from the manufacturing.

Table S2. List of synthesized oligonucleotides

ON Name	Sequence in 5'→3' direction with primer regions underlined
**19ON_A**	CATGGGCGGCATGGGAGGG
**19DNA_A**	CATGGGCGGCATGGGAGGG
**19ON_A^{PSH}** [a]	CATGGGCGGCATGGG**A^{PSH}**GGG
**19DNA_A^{PSH}** [a]	CATGGGCGGCATGGG**A^{PSH}**GGG
**19ON_A^{ASH}** [a]	CATGGGCGGCATGGG**A^{ASH}**GGG
**19DNA_A^{ASH}** [a]	CATGGGCGGCATGGG**A^{ASH}**GGG
**19ON_A^{THT}** [a]	CATGGGCGGCATGGG**A^{THT}**GGG
**19DNA_A^{THT}** [a]	CATGGGCGGCATGGG**A^{THT}**GGG
**19ON_C^{Im}** [a]	CATGGGCGGCATGGG**C^{Im}**GGG
**19DNA_C^{Im}** [a]	CATGGGCGGCATGGG**C^{Im}**GGG
**19DNA_U^{COOH}** [a]	CATGGGCGGCATGGG**U^{COOH}**GGG
**19ON_G^{OH}** [a]	CATGGGCGGCATGGG**G^{OH}**TTT
**19DNA_G^{OH}** [a]	CATGGGCGGCATGGG**G^{OH}**TTT
**31ON**	CATGGGCGGCATGGGACTGAGCTCATGCTAG
**31DNA**	CATGGGCGGCATGGGACTGAGCTCATGCTAG

**31ON_A**$^{\mathbf{PSH}}$ [a]	CATGGGCGGCATGGG**A**$^{\mathbf{PSH}}$CTG**A**$^{\mathbf{PSH}}$GCTC**A**$^{\mathbf{PSH}}$TGCT**A**$^{\mathbf{PSH}}$G
**31DNA_A**$^{\mathbf{PSH}}$ [a]	CATGGGCGGCATGGG**A**$^{\mathbf{PSH}}$CTG**A**$^{\mathbf{PSH}}$GCTC**A**$^{\mathbf{PSH}}$TGCT**A**$^{\mathbf{PSH}}$G
**31ON_A**$^{\mathbf{ASH}}$ [a]	CATGGGCGGCATGGG**A**$^{\mathbf{ASH}}$CTG**A**$^{\mathbf{ASH}}$GCTC**A**$^{\mathbf{ASH}}$TGCT**A**$^{\mathbf{ASH}}$G
**31DNA_A**$^{\mathbf{ASH}}$ [a]	CATGGGCGGCATGGG**A**$^{\mathbf{ASH}}$CTG**A**$^{\mathbf{ASH}}$GCTC**A**$^{\mathbf{ASH}}$TGCT**A**$^{\mathbf{ASH}}$G
**31ON_A**$^{\mathbf{THT}}$ [a]	CATGGGCGGCATGGG**A**$^{\mathbf{THT}}$CTG**A**$^{\mathbf{THT}}$GCTC**A**$^{\mathbf{THT}}$TGCT**A**$^{\mathbf{THT}}$G
**31DNA_A**$^{\mathbf{THT}}$ [a]	CATGGGCGGCATGGG**A**$^{\mathbf{THT}}$CTG**A**$^{\mathbf{THT}}$GCTC**A**$^{\mathbf{THT}}$TGCT**A**$^{\mathbf{THT}}$G
**31ON_C**$^{\mathbf{Im}}$ [a]	CATGGGCGGCATGGGA**C**$^{\mathbf{Im}}$TGAG**C**$^{\mathbf{Im}}$T**C**$^{\mathbf{Im}}$ATG**C**$^{\mathbf{Im}}$TAG
**31DNA_C**$^{\mathbf{Im}}$ [a]	CATGGGCGGCATGGGA**C**$^{\mathbf{Im}}$TGAG**C**$^{\mathbf{Im}}$T**C**$^{\mathbf{Im}}$ATG**C**$^{\mathbf{Im}}$TAG
**31ON_U**$^{\mathbf{COOH}}$ [a]	CATGGGCGGCATGGGAC**U**$^{\mathbf{COOH}}$GAGC**U**$^{\mathbf{COOH}}$CA**U**$^{\mathbf{COOH}}$GC**U**$^{\mathbf{COOH}}$AG
**31DNA_U**$^{\mathbf{COOH}}$ [a]	CATGGGCGGCATGGGAC**U**$^{\mathbf{COOH}}$GAGC**U**$^{\mathbf{COOH}}$CA**U**$^{\mathbf{COOH}}$GC**U**$^{\mathbf{COOH}}$AG
**31ON_G**$^{\mathbf{OH}}$ [a]	CATGGGCGGCATGGGACT**G**$^{\mathbf{OH}}$A**G**$^{\mathbf{OH}}$CTCAT**G**$^{\mathbf{OH}}$CTA**G**$^{\mathbf{OH}}$
**31DNA_G**$^{\mathbf{OH}}$ [a]	CATGGGCGGCATGGGACT**G**$^{\mathbf{OH}}$A**G**$^{\mathbf{OH}}$CTCAT**G**$^{\mathbf{OH}}$CTA**G**$^{\mathbf{OH}}$
**31DNA_G**$^{\mathbf{OH}}$**C**$^{\mathbf{Im}}$ [a]	CATGGGCGGCATGGGA**C**$^{\mathbf{Im}}$T**G**$^{\mathbf{OH}}$A**G**$^{\mathbf{OH}}$**C**$^{\mathbf{Im}}$T**C**$^{\mathbf{Im}}$AT**G**$^{\mathbf{OH}}$**C**$^{\mathbf{Im}}$TA**G**$^{\mathbf{OH}}$
**31DNA_G**$^{\mathbf{OH}}$**U**$^{\mathbf{COOH}}$ [a]	CATGGGCGGCATGGGAC**U**$^{\mathbf{COOH}}$**G**$^{\mathbf{OH}}$A**G**$^{\mathbf{OH}}$C**U**$^{\mathbf{COOH}}$CA**U**$^{\mathbf{COOH}}$**G**$^{\mathbf{OH}}$C**U**$^{\mathbf{COOH}}$A**G**$^{\mathbf{OH}}$
**31DNA_C**$^{\mathbf{Im}}$**U**$^{\mathbf{COOH}}$ [a]	CATGGGCGGCATGGGA**C**$^{\mathbf{Im}}$**U**$^{\mathbf{COOH}}$GAG**C**$^{\mathbf{Im}}$**U**$^{\mathbf{COOH}}$**C**$^{\mathbf{Im}}$A**U**$^{\mathbf{COOH}}$G**C**$^{\mathbf{Im}}$**U**$^{\mathbf{COOH}}$AG
**31DNA_A**$^{\mathbf{PSH}}$**C**$^{\mathbf{Im}}$ [a]	CATGGGCGGCATGGG**A**$^{\mathbf{PSH}}$**C**$^{\mathbf{Im}}$TG**A**$^{\mathbf{PSH}}$G**C**$^{\mathbf{Im}}$T**C**$^{\mathbf{Im}}$**A**$^{\mathbf{PSH}}$TG**C**$^{\mathbf{Im}}$T**A**$^{\mathbf{PSH}}$G
**31DNA_A**$^{\mathbf{PSH}}$**U**$^{\mathbf{COOH}}$ [a]	CATGGGCGGCATGGG**A**$^{\mathbf{PSH}}$C**U**$^{\mathbf{COOH}}$G**A**$^{\mathbf{PSH}}$GC**U**$^{\mathbf{COOH}}$C**A**$^{\mathbf{PSH}}$**U**$^{\mathbf{COOH}}$GC**U**$^{\mathbf{COOH}}$**A**$^{\mathbf{PSH}}$G

**31DNA_A^{PSH}G^{OH} [a]**	CATGGGCGGCATGGGA^{PSH}CTG^{OH}A^{PSH}G^{OH}CTCA^{PSH}TG^{OH}CTA^{PSH}G^{OH}
**31DNA_C^{Im}U^{COOH}A^{PSH} [a]**	CATGGGCGGCATGGGA^{PSH}C^{Im}U^{COOH}GA^{PSH}GC^{Im}U^{COOH}C^{Im}A^{PSH}U^{COOH}GC^{Im}U^{COOH}A^{PSH}G
**31DNA_C^{Im}U^{COOH}G^{OH} [a]**	CATGGGCGGCATGGGAC^{Im}U^{COOH}G^{OH}AG^{OH}C^{Im}U^{COOH}C^{Im}AU^{COOH}G^{OH}C^{Im}U^{COOH}AG^{OH}
**31DNA_A^{PSH}G^{OH}U^{COOH} [a]**	CATGGGCGGCATGGGA^{PSH}CU^{COOH}G^{OH}A^{PSH}G^{OH}CU^{COOH}CA^{PSH}U^{COOH}G^{OH}CU^{COOH}A^{PSH}G^{OH}
**31DNA_A^{PSH}G^{OH}C^{Im} [a]**	CATGGGCGGCATGGGA^{PSH}C^{Im}TG^{OH}A^{PSH}G^{OH}C^{Im}TC^{Im}A^{PSH}TG^{OH}C^{Im}TA^{PSH}G^{OH}
**31ON_C^{Im}U^{COOH}A^{PSH}G^{OH} [a]**	CATGGGCGGCATGGGA^{PSH}C^{Im}U^{COOH}G^{OH}A^{PSH}G^{OH}C^{Im}U^{COOH}C^{Im}A^{PSH}U^{COOH}G^{OH}C^{Im}U^{COOH}A^{PSH}G^{OH}
**31DNA_C^{Im}U^{COOH}A^{PSH}G^{OH} [a]**	CATGGGCGGCATGGGA^{PSH}C^{Im}U^{COOH}G^{OH}A^{PSH}G^{OH}C^{Im}U^{COOH}C^{Im}A^{PSH}U^{COOH}G^{OH}C^{Im}U^{COOH}A^{PSH}G^{OH}
**31ON_C^{Im}U^{COOH}A^{ASH}G^{OH} [a]**	CATGGGCGGCATGGGA^{ASH}C^{Im}U^{COOH}G^{OH}A^{ASH}G^{OH}C^{Im}U^{COOH}C^{Im}A^{ASH}U^{COOH}G^{OH}C^{Im}U^{COOH}A^{ASH}G^{OH}
**31DNA_C^{Im}U^{COOH}A^{ASH}G^{OH} [a]**	CATGGGCGGCATGGGA^{ASH}C^{Im}U^{COOH}G^{OH}A^{ASH}G^{OH}C^{Im}U^{COOH}C^{Im}A^{ASH}U^{COOH}G^{OH}C^{Im}U^{COOH}A^{ASH}G^{OH}
**31ON_C^{Im}U^{COOH}A^{THT}G^{OH} [a]**	CATGGGCGGCATGGGA^{THT}C^{Im}U^{COOH}G^{OH}A^{THT}G^{OH}C^{Im}U^{COOH}C^{Im}A^{THT}U^{COOH}G^{OH}C^{Im}U^{COOH}A^{THT}G^{OH}
**31DNA_C^{Im}U^{COOH}A^{THT}G^{OH} [a]**	CATGGGCGGCATGGGA^{THT}C^{Im}U^{COOH}G^{OH}A^{THT}G^{OH}C^{Im}U^{COOH}C^{Im}A^{THT}U^{COOH}G^{OH}C^{Im}U^{COOH}A^{THT}G^{OH}
**31ON_C^{Im}U^{EPh}A^{PSH}G^{AiPr} [a]**	CATGGGCGGCATGGGA^{PSH}C^{Im}U^{EPh}G^{AiPr}A^{PSH}G^{AiPr}C^{Im}U^{EPh}C^{Im}A^{PSH}U^{EPh}G^{AiPr}C^{Im}U^{EPh}A^{PSH}G^{AiPr}
**31DNA_C^{Im}U^{EPh}A^{PSH}G^{AiPr} [a]**	CATGGGCGGCATGGGA^{PSH}C^{Im}U^{EPh}G^{AiPr}A^{PSH}G^{AiPr}C^{Im}U^{EPh}C^{Im}A^{PSH}U^{EPh}G^{AiPr}C^{Im}U^{EPh}A^{PSH}G^{AiPr}
**43DNA**	CATGGGCGGCATGGGACTGAGCTCATGCTAGACTGAGCTCATG
**43DNA_C^{Im}U^{COOH}A^{PSH}G^{OH} [a]**	CATGGGCGGCATGGGA^{PSH}C^{Im}U^{COOH}G^{OH}A^{PSH}G^{OH}C^{Im}U^{COOH}C^{Im}A^{PSH}U^{COOH}G^{OH}C^{Im}U^{COOH}A^{PSH}G^{OH}A^{PSH}C^{Im}U^{COOH}G^{OH}A^{PSH}G^{OH}C^{Im}U^{COOH}C^{Im}A^{PSH}U^{COOH}G^{OH}
**61DNA**	CAAGGACAAAATACCTGTATTCCTTACTGAGCTCATGCTAGGCGATGTCTCTCATGATGTC

**61DNA_$\mathbf{C^{Im}U^{COOH}A^{PSH}G^{OH}}$** [a]	<u>CAAGGACAAAATACCTGTATTCCTT</u>$\mathbf{A^{PSH}C^{Im}U^{COOH}G^{OH}A^{PSH}G^{OH}C^{Im}U^{COOH}C^{Im}A^{PSH}U^{COOH}G^{OH}C^{Im}U^{COOH}A^{PSH}G^{OH}G^{OH}C^{Im}G^{OH}A^{PSH}U^{COOH}G^{OH}U^{COOH}C^{Im}U^{COOH}C^{Im}U^{COOH}C^{Im}A^{PSH}U^{COOH}G^{OH}A^{PSH}U^{COOH}G^{OH}U^{COOH}C^{Im}}$
**61DNA_$\mathbf{C^{Im}U^{EPh}A^{PSH}G^{AiPr}}$** [a]	<u>CAAGGACAAAATACCTGTATTCCTT</u>$\mathbf{A^{PSH}C^{Im}U^{EPh}G^{AiPr}A^{PSH}G^{AiPr}C^{Im}U^{EPh}C^{Im}A^{PSH}U^{EPh}G^{AiPr}C^{Im}U^{EPh}A^{PSH}G^{AiPr}G^{AiPr}C^{Im}G^{AiPr}A^{PSH}U^{EPh}G^{AiPr}U^{EPh}C^{Im}U^{EPh}C^{Im}U^{EPh}C^{Im}A^{PSH}U^{EPh}G^{AiPr}A^{PSH}U^{EPh}G^{AiPr}U^{EPh}C^{Im}}$
**120DNA**	<u>CAAGGACAAAATACCTGTATTCCTT</u>GCTCATGCTAGACTGAGCTCATGCTAGACTGAGCTCATGCTAGACTGAGCTCATGCTAGACTGAGCTCATGCTAGGCGATGTCTCTCATGATGTC
**120DNA_$\mathbf{C^{Im}U^{COOH}A^{PSH}G^{OH}}$**	<u>CAAGGACAAAATACCTGTATTCCTT</u>$\mathbf{G^{OH}C^{Im}U^{COOH}C^{Im}A^{PSH}U^{COOH}G^{OH}C^{Im}U^{COOH}A^{PSH}G^{OH}A^{PSH}C^{Im}U^{COOH}G^{OH}A^{PSH}G^{OH}C^{Im}U^{COOH}C^{Im}A^{PSH}U^{COOH}G^{OH}C^{Im}U^{COOH}A^{PSH}G^{OH}A^{PSH}C^{Im}U^{COOH}G^{OH}A^{PSH}G^{OH}C^{Im}A^{PSH}U^{COOH}G^{OH}C^{Im}U^{COOH}A^{PSH}G^{OH}A^{PSH}C^{Im}U^{COOH}G^{OH}A^{PSH}G^{OH}C^{Im}U^{COOH}C^{Im}A^{PSH}U^{COOH}G^{OH}C^{Im}U^{COOH}A^{PSH}G^{OH}A^{PSH}C^{Im}U^{COOH}G^{OH}A^{PSH}G^{OH}C^{Im}U^{COOH}C^{Im}A^{PSH}U^{COOH}G^{OH}C^{Im}U^{COOH}A^{PSH}G^{OH}G^{OH}C^{Im}G^{OH}A^{PSH}U^{COOH}G^{OH}U^{COOH}C^{Im}U^{COOH}C^{Im}U^{COOH}C^{Im}A^{PSH}U^{COOH}G^{OH}A^{PSH}U^{COOH}G^{OH}U^{COOH}C^{Im}}$
**98PCR** [a,b]	<u>CAAGGACAAAATACCTGTATTCCTT</u>GCCTGTCCAGGGATCTGCTCTTACAGATTAGAAGTAGTCCTATTAGCCCAGAGGCGATGTCTCTCATGATGTC
**98PCR_$\mathbf{A^{PSH}}$** [a,b]	<u>CAAGGACAAAATACCTGTATTCCTT</u>GCCTGTCC$\mathbf{A^{PSH}}$GGG$\mathbf{A^{PSH}}$TCTGCTCTT$\mathbf{A^{PSH}}$C$\mathbf{A^{PSH}}$G$\mathbf{A^{PSH}}$TT$\mathbf{A^{PSH}}$G$\mathbf{A^{PSH}}$$\mathbf{A^{PSH}}GT\mathbf{A^{PSH}}$GTCCT$\mathbf{A^{PSH}}TT\mathbf{A^{PSH}}$GCCC$\mathbf{A^{PSH}}G\mathbf{A^{PSH}}$GGCG$\mathbf{A^{PSH}}$TGTCTCTC$\mathbf{A^{PSH}}TG\mathbf{A^{PSH}}$TGTC
**98PCR_$\mathbf{A^{ASH}}$** [a,b]	<u>CAAGGACAAAATACCTGTATTCCTT</u>GCCTGTCC$\mathbf{A^{ASH}}$GGG$\mathbf{A^{ASH}}$TCTGCTCTT$\mathbf{A^{ASH}}$C$\mathbf{A^{ASH}}$G$\mathbf{A^{ASH}}$TT$\mathbf{A^{ASH}}$G$\mathbf{A^{ASH}}$$\mathbf{A^{ASH}}GT\mathbf{A^{ASH}}$GTCCT$\mathbf{A^{ASH}}TT\mathbf{A^{ASH}}$GCCC$\mathbf{A^{ASH}}G\mathbf{A^{ASH}}$GGCG$\mathbf{A^{ASH}}$TGTCTCTC$\mathbf{A^{ASH}}TG\mathbf{A^{ASH}}$TGTC
**98PCR_$\mathbf{A^{THT}}$** [a,b]	<u>CAAGGACAAAATACCTGTATTCCTT</u>GCCTGTCC$\mathbf{A^{THT}}$GGG$\mathbf{A^{THT}}$TCTGCTCTT$\mathbf{A^{THT}}$C$\mathbf{A^{THT}}$G$\mathbf{A^{THT}}$TT$\mathbf{A^{THT}}$G$\mathbf{A^{THT}}$$\mathbf{A^{THT}}GT\mathbf{A^{THT}}$GTCCT$\mathbf{A^{THT}}TT\mathbf{A^{THT}}$GCCC

$\mathbf{A}^{THT}$G$\mathbf{A}^{THT}$GGCG$\mathbf{A}^{THT}$TGTCTCTC$\mathbf{THT}^{cS}$TG$\mathbf{A}^{THT}$TGTC

98PCR_C^{Im} [a,b]

CAAGGACAAAATACCTGTATTCCTTG$\mathbf{C}^{Im}\mathbf{C}^{Im}TGT\mathbf{C}^{Im}\mathbf{C}^{Im}$AGGGAT$\mathbf{C}^{Im}TG\mathbf{C}^{Im}T\mathbf{C}^{Im}TTA\mathbf{C}^{Im}$AGATTAGAAGTAGT$\mathbf{C}^{Im}\mathbf{C}^{Im}$TATTAG$\mathbf{C}^{Im}\mathbf{C}^{Im}\mathbf{C}^{Im}$AGAGG$\mathbf{C}^{Im}$GATGT$\mathbf{C}^{Im}T\mathbf{C}^{Im}T\mathbf{C}^{Im}$ATGATGT$\mathbf{C}^{Im}$

98PCR_U^{COOH} [a,b]

CAAGGACAAAATACCTGTATTCCTTGCC$\mathbf{U}^{COOH}$G$\mathbf{U}^{COOH}$CCAGGGA$\mathbf{U}^{COOH}$C$\mathbf{U}^{COOH}$GC$\mathbf{U}^{COOH}$C$\mathbf{U}^{COOH}\mathbf{U}^{COOH}$ACAGA$\mathbf{U}^{COOH}\mathbf{U}^{COOH}$AGAAG$\mathbf{U}^{COOH}AG\mathbf{U}^{COOH}CC\mathbf{U}^{COOH}A\mathbf{U}^{COOH}$AGCCCAGAGGCGA$\mathbf{U}^{COOH}G\mathbf{U}^{COOH}C\mathbf{U}^{COOH}C\mathbf{U}^{COOH}CA\mathbf{U}^{COOH}GA\mathbf{U}^{COOH}G\mathbf{U}^{COOH}$C

98PCR_G^{OH} [a,b]

CAAGGACAAAATACCTGTATTCCTT$\mathbf{G}^{OH}$CCT$\mathbf{G}^{OH}$TCCA$\mathbf{G}^{OH}\mathbf{G}^{OH}\mathbf{G}^{OH}$ATCT$\mathbf{G}^{OH}$CTCTTACA$\mathbf{G}^{OH}$ATTA$\mathbf{G}^{OH}AA\mathbf{G}^{OH}TA\mathbf{G}^{OH}$TCCTATTA$\mathbf{G}^{OH}$CCCA$\mathbf{G}^{OH}A\mathbf{G}^{OH}\mathbf{G}^{OH}C\mathbf{G}^{OH}AT\mathbf{G}^{OH}$TCTCTCAT$\mathbf{G}^{OH}AT\mathbf{G}^{OH}$TC

98PCR_C^{Im}**U**^{COOH} [a,b]

CAAGGACAAAATACCTGTATTCCTTG$\mathbf{C}^{Im}\mathbf{C}^{Im}\mathbf{U}^{COOH}G\mathbf{U}^{COOH}\mathbf{C}^{Im}\mathbf{C}^{Im}$AGGGA$\mathbf{U}^{COOH}\mathbf{C}^{Im}\mathbf{U}^{COOH}G\mathbf{C}^{Im}\mathbf{U}^{COOH}\mathbf{C}^{Im}\mathbf{U}^{COOH}\mathbf{U}^{COOH}A\mathbf{C}^{Im}AGA\mathbf{U}^{COOH}\mathbf{U}^{COOH}$AGAAG$\mathbf{U}^{COOH}AG\mathbf{U}^{COOH}\mathbf{C}^{Im}\mathbf{C}^{Im}\mathbf{U}^{COOH}A\mathbf{U}^{COOH}\mathbf{U}^{COOH}AG\mathbf{C}^{Im}\mathbf{C}^{Im}\mathbf{C}^{Im}$AGAGG$\mathbf{C}^{Im}GA\mathbf{U}^{COOH}G\mathbf{U}^{COOH}\mathbf{C}^{Im}\mathbf{U}^{COOH}\mathbf{C}^{Im}\mathbf{U}^{COOH}\mathbf{C}^{Im}A\mathbf{U}^{COOH}GA\mathbf{U}^{COOH}G\mathbf{U}^{COOH}\mathbf{C}^{Im}$

98PCR_C^{Im}**G**^{OH} [a,b]

CAAGGACAAAATACCTGTATTCCTT$\mathbf{G}^{OH}\mathbf{C}^{Im}\mathbf{C}^{Im}T\mathbf{G}^{OH}T\mathbf{C}^{Im}\mathbf{C}^{Im}A\mathbf{G}^{OH}\mathbf{G}^{OH}\mathbf{G}^{OH}AT\mathbf{C}^{Im}T\mathbf{G}^{OH}\mathbf{C}^{Im}T\mathbf{C}^{Im}TTA\mathbf{C}^{Im}A\mathbf{G}^{OH}$ATTA$\mathbf{G}^{OH}AA\mathbf{G}^{OH}TA\mathbf{G}^{OH}T\mathbf{C}^{Im}\mathbf{C}^{Im}$TATTA$\mathbf{G}^{OH}\mathbf{C}^{Im}\mathbf{C}^{Im}\mathbf{C}^{Im}A\mathbf{G}^{OH}A\mathbf{G}^{OH}\mathbf{G}^{OH}\mathbf{C}^{Im}\mathbf{G}^{OH}AT\mathbf{G}^{OH}T\mathbf{C}^{Im}T\mathbf{C}^{Im}T\mathbf{C}^{Im}AT\mathbf{G}^{OH}AT\mathbf{G}^{OH}T\mathbf{C}^{Im}$

98PCR_U^{COOH}**G**^{OH} [a,b]

CAAGGACAAAATACCTGTATTCCTT$\mathbf{G}^{OH}$CC$\mathbf{U}^{COOH}\mathbf{G}^{OH}\mathbf{U}^{COOH}CCA\mathbf{G}^{OH}\mathbf{G}^{OH}\mathbf{G}^{OH}A\mathbf{U}^{COOH}C\mathbf{U}^{COOH}\mathbf{G}^{OH}C\mathbf{U}^{COOH}C\mathbf{U}^{COOH}\mathbf{U}^{COOH}ACA\mathbf{G}^{OH}A\mathbf{U}^{COOH}\mathbf{U}^{COOH}A\mathbf{G}^{OH}AA\mathbf{G}^{OH}\mathbf{U}^{COOH}A\mathbf{G}^{OH}\mathbf{U}^{COOH}CC\mathbf{U}^{COOH}A\mathbf{U}^{COOH}A\mathbf{G}^{OH}$CCCA$\mathbf{G}^{OH}A\mathbf{G}^{OH}\mathbf{G}^{OH}C\mathbf{G}^{OH}A\mathbf{U}^{COOH}\mathbf{G}^{OH}\mathbf{U}^{COOH}C\mathbf{U}^{COOH}C\mathbf{U}^{COOH}CA\mathbf{U}^{COOH}\mathbf{G}^{OH}A\mathbf{U}^{COOH}\mathbf{G}^{OH}\mathbf{U}^{COOH}$C

98PCR_A^{PSH}**G**^{OH} [a,b]

CAAGGACAAAATACCTGTATTCCTT$\mathbf{G}^{OH}$CCT$\mathbf{G}^{OH}$TCC$\mathbf{A}^{PSH}\mathbf{G}^{OH}\mathbf{G}^{OH}\mathbf{G}^{OH}\mathbf{A}^{PSH}TCT\mathbf{G}^{OH}$CTCTT$\mathbf{A}^{PSH}C\mathbf{A}^{PSH}\mathbf{G}^{OH}\mathbf{A}^{PSH}TT\mathbf{A}^{PSH}\mathbf{G}^{OH}\mathbf{A}^{PSH}\mathbf{A}^{PSH}\mathbf{G}^{OH}T\mathbf{A}^{PSH}\mathbf{G}^{OH}$TCCT$\mathbf{A}^{PSH}TT\mathbf{A}^{PSH}\mathbf{G}^{OH}CCC\mathbf{A}^{PSH}\mathbf{G}^{OH}\mathbf{A}^{PSH}\mathbf{G}^{OH}\mathbf{G}^{OH}C\mathbf{G}^{OH}\mathbf{A}^{PSH}T\mathbf{G}^{OH}$TCTCTC$\mathbf{A}^{PSH}T\mathbf{G}^{OH}\mathbf{A}^{PSH}T\mathbf{G}^{OH}$TC

**98PCR_A^{PSH}C^{Im}** [a,b]	CAAGGACAAAATACCTGTATTCCTTGC^{Im}C^{Im}TGTC^{Im}C^{Im}A^{PSH}GGGA^{PSH}TC^{Im}TGC^{Im}TC^{Im}TTA^{PSH}C^{Im}A^{PSH}GA^{PSH}TTA^{PSH}GA^{PSH}A^{PSH}GTA^{PSH}GTC^{Im}C^{Im}TA^{PSH}TTA^{PSH}GC^{Im}C^{Im}C^{Im}A^{PSH}GA^{PSH}GGC^{Im}GA^{PSH}TGTC^{Im}TC^{Im}TC^{Im}A^{PSH}TGA^{PSH}TGTC^{Im}
**98PCR_A^{PSH}U^{COOH}** [a,b]	CAAGGACAAAATACCTGTATTCCTTGCCU^{COOH}GU^{COOH}CCA^{PSH}GGGA^{PSH}U^{COOH}CU^{COOH}GCU^{COOH}CU^{COOH}U^{COOH}A^{PSH}CA^{PSH}GA^{PSH}U^{COOH}U^{COOH}A^{PSH}GA^{PSH}A^{PSH}GU^{COOH}A^{PSH}GU^{COOH}CCU^{COOH}A^{PSH}U^{COOH}A^{PSH}GCCCA^{PSH}GA^{PSH}GGCGA^{PSH}U^{COOH}GU^{COOH}CU^{COOH}CU^{COOH}CA^{PSH}U^{COOH}GA^{PSH}U^{COOH}GU^{COOH}C
**98PCR_C^{Im}U^{COOH}G^{OH}** [a]	CAAGGACAAAATACCTGTATTCCTTG^{OH}C^{Im}C^{Im}U^{COOH}G^{OH}U^{COOH}C^{Im}C^{Im}AG^{OH}G^{OH}G^{OH}AU^{COOH}C^{Im}U^{COOH}G^{OH}C^{Im}U^{COOH}C^{Im}U^{COOH}U^{COOH}A^{PSH}C^{Im}AG^{OH}AU^{COOH}U^{COOH}AG^{OH}AAG^{OH}U^{COOH}AG^{OH}U^{COOH}C^{Im}C^{Im}U^{COOH}AU^{COOH}U^{COOH}AG^{OH}C^{Im}C^{Im}C^{Im}AG^{OH}AG^{OH}G^{OH}C^{Im}G^{OH}AU^{COOH}G^{OH}U^{COOH}C^{Im}U^{COOH}C^{Im}U^{COOH}C^{Im}AU^{COOH}G^{OH}AU^{COOH}G^{OH}U^{COOH}C^{Im}
**98PCR_C^{Im}U^{COOH}A^{PSH}G^{OH}** [a]	CAAGGACAAAATACCTGTATTCCTTG^{OH}C^{Im}C^{Im}U^{COOH}G^{OH}U^{COOH}C^{Im}C^{Im}A^{PSH}G^{OH}G^{OH}G^{OH}A^{PSH}U^{COOH}C^{Im}U^{COOH}G^{OH}C^{Im}U^{COOH}C^{Im}U^{COOH}U^{COOH}A^{PSH}C^{Im}A^{PSH}G^{OH}A^{PSH}U^{COOH}U^{COOH}A^{PSH}G^{OH}A^{PSH}A^{PSH}G^{OH}U^{COOH}A^{PSH}G^{OH}U^{COOH}C^{Im}C^{Im}U^{COOH}A^{PSH}U^{COOH}U^{COOH}A^{PSH}G^{OH}C^{Im}C^{Im}C^{Im}A^{PSH}G^{OH}A^{PSH}G^{OH}G^{OH}C^{Im}G^{OH}A^{PSH}U^{COOH}G^{OH}U^{COOH}C^{Im}U^{COOH}C^{Im}U^{COOH}C^{Im}A^{PSH}U^{COOH}G^{OH}A^{PSH}U^{COOH}G^{OH}U^{COOH}C^{Im}
**118ON_C^{Im}U^{COOH}A^{PSH}G^{OH}** [a]	CATTCGGCTGCTCTTGATTTCAAGGACAAAATACCTGTATTCCTTG^{OH}C^{Im}C^{Im}U^{COOH}G^{OH}U^{COOH}C^{Im}C^{Im}A^{PSH}G^{OH}G^{OH}G^{OH}A^{PSH}U^{COOH}C^{Im}U^{COOH}G^{OH}C^{Im}U^{COOH}C^{Im}U^{COOH}U^{COOH}A^{PSH}C^{Im}A^{PSH}G^{OH}A^{PSH}U^{COOH}U^{COOH}A^{PSH}G^{OH}A^{PSH}A^{PSH}G^{OH}U^{COOH}A^{PSH}G^{OH}U^{COOH}C^{Im}C^{Im}U^{COOH}A^{PSH}U^{COOH}U^{COOH}A^{PSH}G^{OH}C^{Im}C^{Im}C^{Im}A^{PSH}G^{OH}A^{PSH}G^{OH}G^{OH}C^{Im}G^{OH}A^{PSH}U^{COOH}G^{OH}U^{COOH}C^{Im}U^{COOH}C^{Im}U^{COOH}C^{Im}A^{PSH}U^{COOH}G^{OH}A^{PSH}U^{COOH}G^{OH}U^{COOH}C^{Im}
**118rePCR** [a,b]	CATTCGGCTGCTCTTGATTTCAAGGACAAAATACCTGTATTCCTTGCCTGTCCAGGGATCTGCTCTTACAGATTAGAAGTAGTCCTATTAGCCCAGAGGCGATGTCTCTCATGATGTC

**118rePCR**	CATTCGGCTGCTCTTGATTTCAAGGACAAAAT ACCTGTATTCCTTGCCTGTCCAGGGATCTGCT CTTACAGATTAGAAGTAGTCCTATTAGCCCAG AGGCGATGTCTCTCATGATGTC

[a] 5'-(6-FAM); [b] 5'-(Cy5); the complementary strand is an unmodified template, for simplicity not shown; ON = single-stranded DNA; DNA = double-stranded DNA; PCR = double-stranded PCR product

2.1. PEX – Single incorporation (one modified dN^{R}TP)

2.1.1. Single incorporation of dN^{R}TP using 19-mer template – analytical scale

Reaction mixture (20 µL) contained 5'-(6-FAM)-labelled Prim248short (3µM, 1µL), on of templates Oligo 1C/1A/1T/1G (3 µM, 1.5 µL), Vent(exo-) polymerase (1U/µL) (**Figure S1**) or KOD XL DNA polymerase (1U/uL) (**Figure S2**) natural dGTP (or dTTP in case of Oligo 1G) (1 µL), modified **dN^{R}TP** (2 µL) in enzyme reaction buffer (10X, 2 µL). Negative controls were performed in absence of the modified or natural triphosphate of study. The reaction mixture was incubated for 30 min at 60 °C in a thermomixer, stopped by addition of PAGE stop solution (20 µL) and denaturated for 5 min at 95 °C. Samples were separated by use of denaturing PAGE and visualized using fluorescence imaging. For the exact amount/concentration of the polymerase and **dN^{R}TPs**/dNTPs see **Table S3**.

Table S3. Reaction conditions specified for single nucleotide incorporation using Vent(exo-) DNA polymerase and KOD XL DNA polymerase

Templates	**dN^{R}TPs**	dNTPs	Vent(exo-) 1U/µL	KOD XL 1U/µL
Oligo 1A	**dA^{PSH}TP** 0.5 mM	dGTP 0.125 mM*	0.2 µL	0.1 µL
Oligo 1A	**dA^{THT}TP** 0.5 mM	dGTP 0.5 mM	0.2 µL	0.1 µL
Oligo 1A	**dA^{ASH}TP** 0.5 mM	dGTP 0.5 mM	0.2 µL	0.1 µL
Oligo 1C	**dC^{Im}TP** 2 mM	dGTP 0.5 mM	0.6 µL	0.1 µL
Oligo 1T	**dU^{COOH}TP** 2 mM	dGTP 0.5 mM	0.6 µL	0.1 µL
Oligo 1G	**dG^{OH}TP** 0.5 mM	dTTP 0.125 mM	0.2 µL	0.15 µL

* 0.5 mM dGTP in case of KOD XL

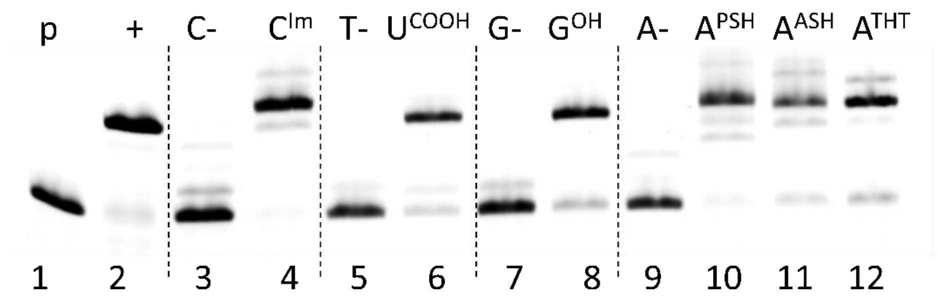

Figure S1. Denaturing PAGE analysis of PEX using Vent(exo–) DNA polymerase, 5′-(6-FAM)-labelled primer Prim248short, and 19-mer appropriate template Oligo 1C/1A/1T/1G; lane (1) primer; lane (2) positive control (dGTP, dATP); lanes (3), (5), (7), (9) negative controls in absence of modified or natural triphosphate of study dCTP, dTTP, dGTP or dATP; lanes (4), (6), (8), (10)-(12) reactions using **dC^{Im}TP**, **dU^{COOH}TP**, **dG^{OH}TP**, **dA^{PSH}TP**, **dA^{ASH}TP** or **dA^{THT}TP** in combination with the appropriate natural dNTPs.

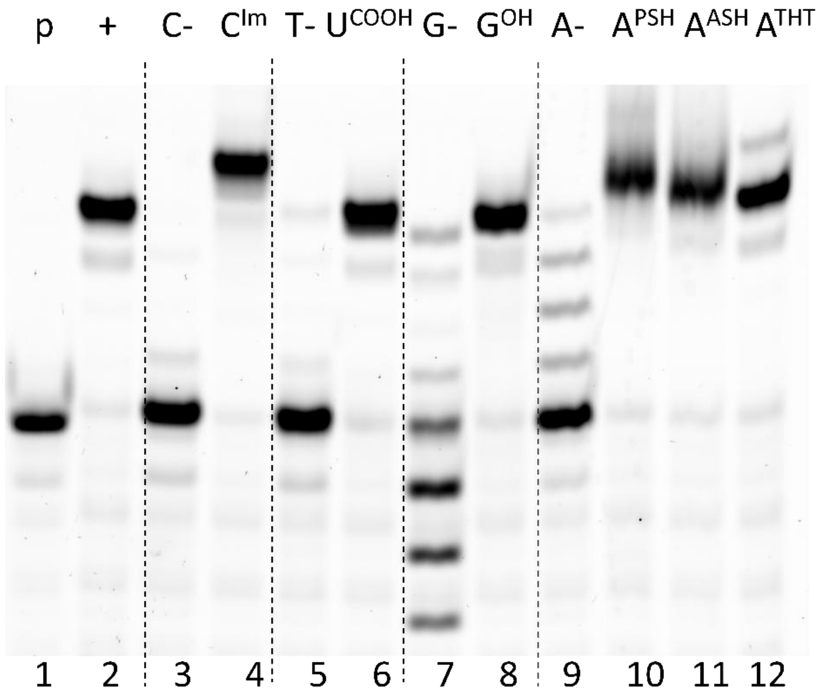

Figure S2. Denaturing PAGE analysis of PEX using KOD XL DNA polymerase, 5′-(6-FAM)-labelled primer Prim248short, and 19-mer appropriate template Oligo 1C/1A/1T/1G; lane (1) primer; lane (2) positive control (dGTP, dATP); lanes (3), (5), (7), (9) negative controls in absence of modified or natural triphosphate of study dCTP, dTTP, dGTP or dATP; lanes (4), (6), (8), (10)-(12) reactions using **dC^{Im}TP**, **dU^{COOH}TP**, **dG^{OH}TP**, **dA^{PSH}TP**, **dA^{ASH}TP** or **dA^{THT}TP** in combination with the appropriate natural dNTPs.

2.1.2. Multiple incorporation (one modified dN^{R}TP) – 31-mer template – analytical scale

The reaction mixture (20 µL) contained 5'-(6-FAM)-labelled primer Prim248short (3 µM, 1 µL), template Prb4basII (3 µM, 1.5 µL), Pwo DNA polymerase (1 U/µL, 0.525 µL) (**Figure S3.**) or KOD XL DNA polymerase (1 U/µL, 0.25 uL) (**Figure S4**), appropriate natural dNTPs (2 µL each), modified **dN^{R}TP**, (2 µL) and enzyme reaction buffer (10X, 2 µL) as supplied by the manufacturer. Negative controls were performed in absence of the modified or natural triphosphate of study. The reaction mixture was incubated for 15 min at 60 °C in a thermomixer, stopped by addition of PAGE stop solution (20 µL) and denatured for 5 min at 95 °C. Samples were separated with a 12.5% denaturing PAGE and visualized using fluorescence imaging. For the exact amount/concentration of **dN^{R}TPs**/dNTPs, see **Table S4**.

Table S4. Reaction conditions specified for multiple nucleotide incorporation - one **dN^{R}TP**

**dN^{R}TPs**	dNTPs
**dA^{PSH}TP** 1 mM	dGTP, dTTP, dCTP; 1 mM
**dA^{THT}TP** 1 mM	dGTP, dTTP, dCTP; 1 mM
**dA^{ASH}TP** 1 mM	dGTP, dTTP, dCTP; 1 mM
**dC^{Im}TP** 2 mM	dGTP, dTTP, dATP; 2 mM
**dU^{COOH}TP** 2 mM	dGTP, dATP, dCTP; 2 mM
**dG^{OH}TP** 1 mM	dTTP, dCTP, dATP; 1 mM

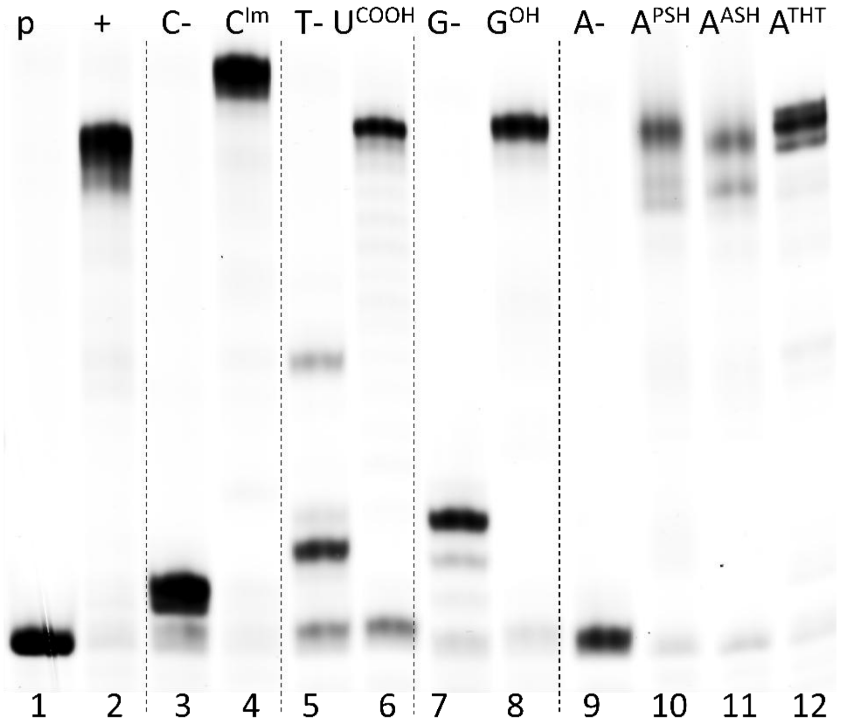

Figure S3. Denaturing PAGE analysis of PEX reaction with one modified **dN^{R}TP** using 5'-(6-FAM)-labelled primer Prim248short, 31-mer template Prb4basII and Pwo DNA polymerase; lane (1) primer; lane (2) positive control (dCTP, dTTP, dGTP and dATP) ; lanes (3), (5), (7), (9) negative controls in absence of modified or natural triphosphate of study dCTP, dTTP, dGTP or dATP; lanes (4), (6), (8), (10)-(12) reactions using **dC^{Im}TP**, **dU^{COOH}TP**, **dG^{OH}TP**, **dA^{PSH}TP**, **dA^{ASH}TP** or **dA^{THT}TP** in combination with the other three natural dNTPs.

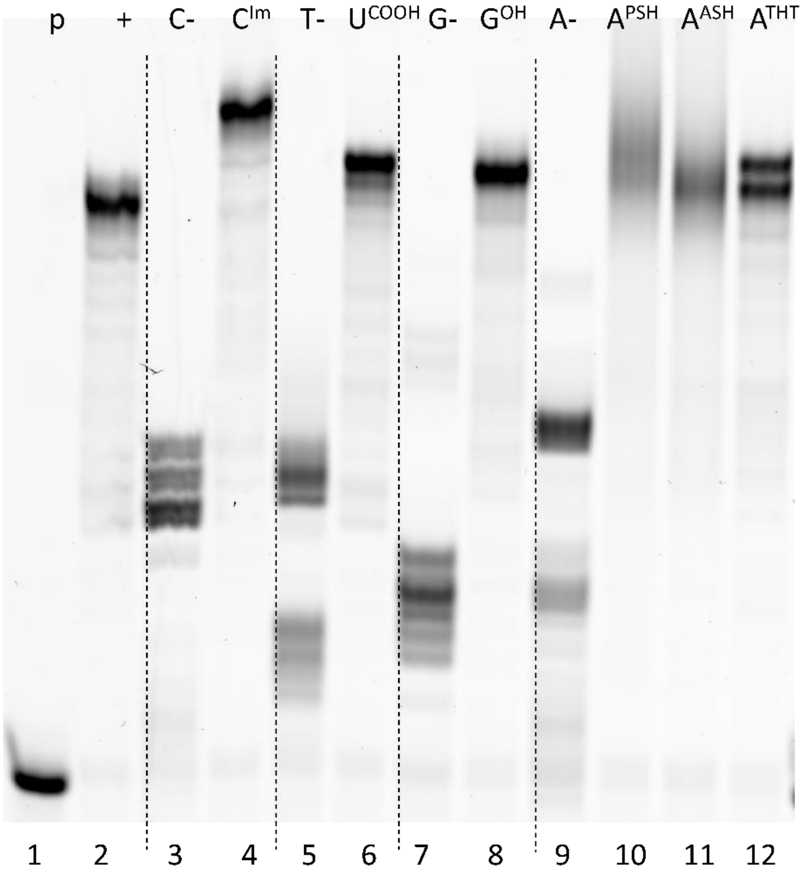

Figure S4. Denaturing PAGE analysis of PEX reaction with one modified **dN^R^TP** using 5'-(6-FAM)-labelled primer Prim248short, 31-mer template Prb4basII and KOD XL DNA polymerase; lane (1) primer; lane (2) positive control (dCTP, dTTP, dGTP and dATP); lanes (3), (5), (7), (9) negative controls in absence of modified or natural triphosphate of study dCTP, dTTP, dGTP or dATP; lanes (4), (6), (8), (10)-(12) reactions using **dC^{Im}TP**, **dU^{COOH}TP**, **dG^{OH}TP**, **dA^{PSH}TP**, **dA^{ASH}TP** or **dA^{THT}TP** in combination with the other three natural dNTPs.

For testing the inhibitory effect of the thiol-modified **dN^{R}TP**s on the DNA polymerases in PEX reaction the reaction mixture (20 μL) contained 5'-(6-FAM)-labelled primer Prim248short (3 μM, 1 μL), template Prb4basII (3 μM, 1.5 μL), either Pwo DNA polymerase (1 U/μL, 0.525 μL) or KOD XL DNA polymerase (1 U/μL, 0.25 uL), set of four natural dNTPs (1 mM, 1 μL each), either **dA^{PSH}TP** or **dA^{ASH}TP** (1 mM, 1 μL) and enzyme reaction buffer (10X, 2 μL) as supplied by the manufacturer. Positive controls were performed in presence of natural dNTPs. The reaction mixture was incubated for 15 min at 60 °C in a thermomixer, stopped by addition of PAGE stop solution (20 μL) and denatured for 5 min at 95 °C. Samples were separated with a 12.5% denaturing PAGE and visualized using fluorescence imaging (**Figure S5**).

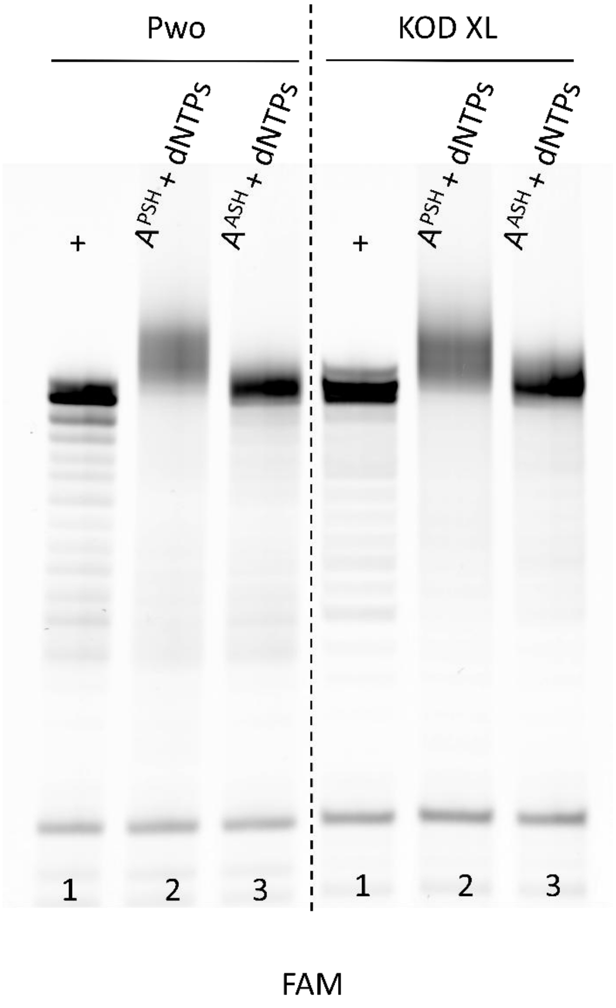

Figure S5. Denaturing PAGE analysis of PEX reaction with one thiol-modified **dN^R^TP (dA^PSH^TP** or **dA^ASH^TP)** and set of four natural dNTPs using 5'-(6-FAM)-labelled primer Prim248short, 31-mer template Prb4basII and either Pwo DNA polymerase (left) or KOD XL DNA polymerase (right); lane (1) positive control (dCTP, dTTP, dGTP and dATP); (2) **dA^{PSH}TP**, dCTP, dTTP, dGTP and dATP; (3) **dA^{ASH}TP**, dCTP, dTTP, dGTP and dATP.

2.1.3. Multiple incorporation (two modified dN^{R}TPs) – 31-mer template – analytical scale

The reaction mixture (20 μL) contained 5'-(6-FAM)-labelled primer Prim248short (3 μM, 1 μL), template Prb4basII (3 μM, 1.5 μL), Pwo DNA polymerase (1 U/μL, 0.525 μL), appropriate natural dNTPs (1 mM, 2 μL), set of two modified **dN^{R}TPs**, (1 mM, 2 μL) and enzyme reaction buffer (10X, 2 μL) as supplied by the manufacturer. Negative controls were performed in absence of one modified or natural triphosphate of study. The reaction mixture was incubated for 30 min at 60 °C in a thermomixer, stopped by addition of PAGE stop solution (20 μL) and denatured for 5 min at

95 °C. Samples were separated with a 12.5% denaturing PAGE and visualized using fluorescence imaging (**Figure S6**).

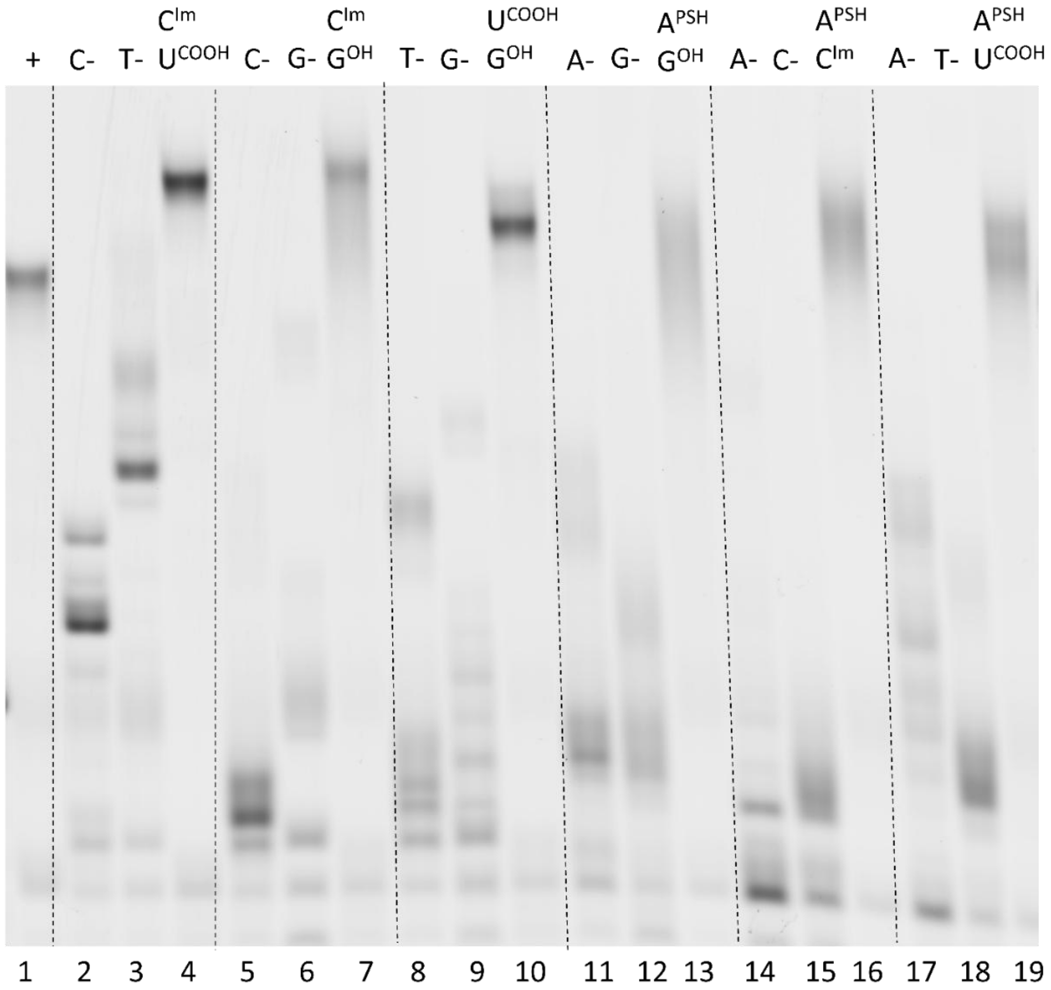

Figure S6. Denaturing PAGE analysis of PEX reaction with two modified **dN^{R}TPs** using 5'-(6-FAM)-labelled primer Prim248short, template Prb4basII; (1): positive control (dCTP, dTTP, dGTP and dATP); (2): dGTP, dATP, **dU^{COOH}TP**; (3): dGTP, dATP, **dC^{Im}TP**; (4): dATP, dGTP, **dC^{Im}TP**, **dU^{COOH}TP**; (5): dATP, dTTP, **dG^{OH}TP**; (6): dATP, dTTP, **dC^{Im}TP**; (7): dATP, dTTP, **dC^{Im}TP**, **dG^{OH}TP**; (8): dATP, dCTP, **dG^{OH}TP**; (9): dATP, dCTP, **dU^{COOH}TP**; (10): dATP, dCTP, **dU^{COOH}TP**, **dG^{OH}TP**; (11): dCTP, dTTP, **dG^{OH}TP**; (12): **dA^{PSH}TP**, dCTP, dTTP; (13): **dA^{PSH}TP**, dCTP, dTTP, **dG^{OH}TP**; (14): **dC^{Im}TP**, dTTP, dGTP; (15): **dA^{PSH}TP**, dTTP, dGTP; (16): **dA^{PSH}TP**, dTTP, dGTP, **dC^{Im}TP**; (17): dGTP, dCTP, **dU^{COOH}TP**; (18): **dA^{PSH}TP**, dGTP, dCTP; (19): **dA^{PSH}TP**, dGTP, dCTP, **dU^{COOH}TP**

2.1.4. Multiple incorporation (three modified dN^{R}TPs) – 31-mer template – analytical scale

The reaction mixture (20 µL) contained primer 5'-(6-FAM)-labelled primer Prim248short (3 µM, 1 µL), template Prb4basII (3 µM, 1.5 µL), Pwo DNA polymerase (1 U/µL, 0.525 µL), appropriate natural dNTPs (1 mM, 2 µL), set of three modified **dN^{R}TPs**, (1 mM, 2 µL) and enzyme reaction buffer (10X, 2 µL) as supplied by the manufacturer. Negative controls were performed in absence of one modified or natural triphosphate of study. The reaction mixture was incubated for 30 min at 60 °C in a thermomixer, stopped by addition of PAGE stop solution (20 µL) and denatured for 5 min at 95 °C. Samples were separated with a 12.5% denaturing PAGE and visualized using fluorescence imaging (**Figure S7**).

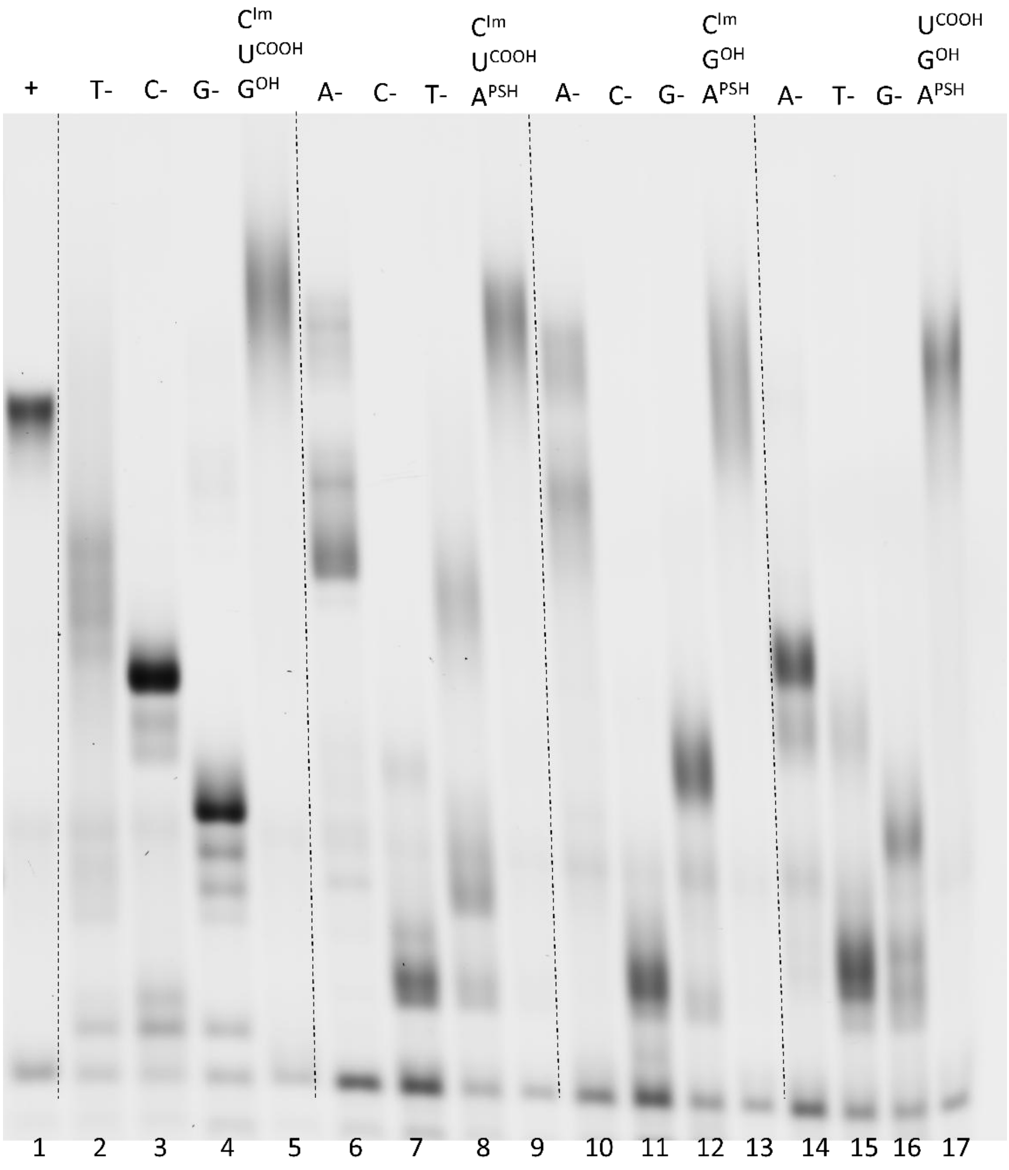

Figure S7. Denaturing PAGE analysis of PEX reaction with three modified **dN^{R}TPs** using 5'-(6-FAM)-labelled primer Prim248short, template Prb4basII; lanes (1) positive control (dCTP, dTTP,

dGTP and dATP); (2) **dG^{OH}TP**, dATP, **dC^{Im}TP**; (3): **dU^{COOH}TP, dG^{OH}TP**, dATP; (4): **dU^{COOH}TP**, **dC^{Im}TP**, dATP; (5): **dU^{COOH}TP**, **dC^{Im}TP**, **dG^{OH}TP**, dATP; (6): **dU^{COOH}TP**, **dC^{Im}TP**, dGTP; (7): **dA^{PSH}TP**, **dU^{COOH}TP**, dGTP, (8): **dA^{PSH}TP**, **dC^{Im}TP**, dGTP; (9): **dA^{PSH}TP**, **dC^{Im}TP, dU^{COOH}TP**, dGTP; (10): **dC^{Im}TP, dG^{OH}TP**, dTTP; (11): **dA^{PSH}TP**, **dG^{OH}TP**, dTTP; (12): **dA^{PSH}TP**, **dC^{Im}TP**, dTTP; (13): **dA^{PSH}TP**, **dC^{Im}TP**, dTTP, **dG^{OH}TP**; (14): **dU^{COOH}TP**, **dG^{OH}TP**, dCTP; (15): **dA^{PSH}TP**, **dG^{OH}TP,** dCTP; (16): **dA^{PSH}TP**, **dU^{COOH}TP**, dCTP; (17): **dA^{PSH}TP**, **dU^{COOH}TP**, **dG^{OH}TP**, dCTP

2.1.5. Multiple incorporation (four modified dN^{R}TPs) – 31-mer template – analytical scale

The reaction mixture (20 μL) contained 5'-(6-FAM)-labelled primer Prim248short (3 μM, 1 μL), template Prb4basII (3 μM, 1.5 μL), Pwo DNA polymerase (1 U/μL, 0.525 μL), set of four modified **dN^{R}TPs**, (1 mM, 1 μL) and enzyme reaction buffer (10X, 2 μL) as supplied by the manufacturer. Negative controls were performed in absence of one modified or natural triphosphate of study.The reaction mixture was incubated for 30 min at 60 °C in a thermomixer, stopped by addition of PAGE stop solution (20 μL) and denatured for 5 min at 95 °C. Samples were separated with a 12.5% denaturing PAGE and visualized using fluorescence imaging. (**Figure S8** and **Figure S9**).

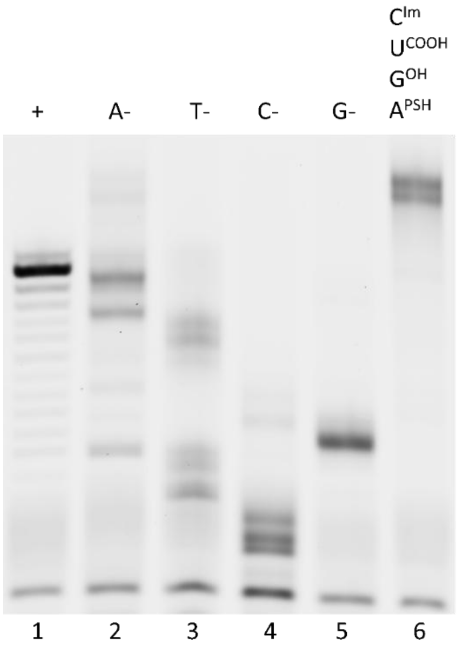

Figure S8. Denaturing PAGE analysis of PEX reaction with four modified **dN^{R}TPs** using 5'-(6-FAM)-labelled primer Prim248short, template Prb4basII; lanes (1) positive control (dCTP, dTTP, dGTP and dATP); (2) **dU^{COOH}TP, dC^{Im}TP, dG^{OH}TP**; (3): **dA^{PSH}TP**, **dC^{Im}TP**, **dG^{OH}TP**; (4): **dA^{PSH}TP**, **dU^{COOH}TP**, **dG^{OH}TP**; (5): **dA^{PSH}TP**, **dC^{Im}TP**, **dU^{COOH}TP**; (6): **dA^{PSH}TP**, **dC^{Im}TP**, **dU^{COOH}TP**, **dG^{OH}TP**

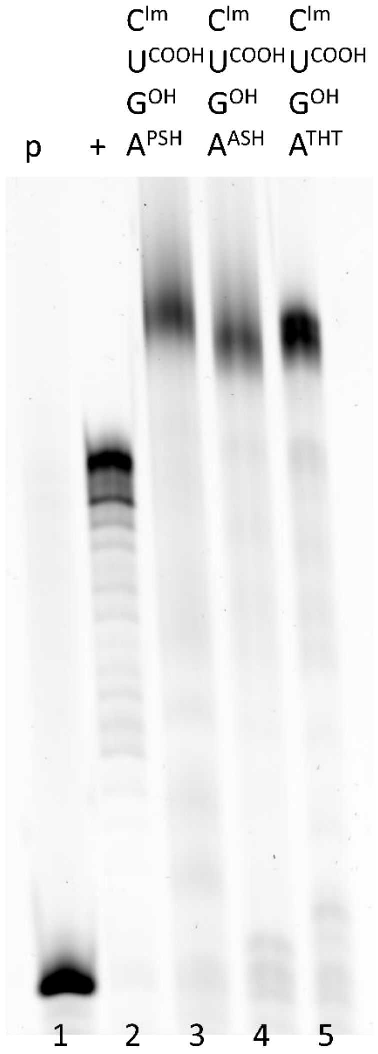

Figure S9. Denaturing PAGE of hypermodified PEX products using different sulphur modified **dN^{R}TPs**; lanes (1) primer; (2) positive control (dCTP, dTTP, dGTP and dATP); (3): **dA^{PSH}TP, dC^{Im}TP, dU^{COOH}TP, dG^{OH}TP**; (4): **dA^{ASH}TP, dC^{Im}TP, dU^{COOH}TP, dG^{OH}TP**; (5): **dA^{THT}TP, dC^{Im}TP, dU^{COOH}TP, dG^{OH}TP**

2.1.6. Multiple incorporation (four modified dN^{R}TPs) – different template lengths – analytical scale

The reaction mixture (20 µL) contained primer 5'-(6-FAM)-labelled primer Prim248short, for 31-, 43-, 61-mer template or 5'-(6-FAM)-labelled primer LT25TH (3 µM, 1 µL) for 98-, 120-mer template (see **Table S5**) (3 µM, 1.5 µL), Pwo DNA polymerase (1 U/µL), set of four modified **dN^{R}TPs** (**dA^{PSH}TP**, **dC^{Im}TP**, **dU^{COOH}TP**, **dG^{OH}TP**), (2 mM, 2 µL) and enzyme reaction buffer (10X, 2 µL) as supplied by the manufacturer. The reaction mixture was incubated for 30 min at 60 °C in a thermomixer, stopped by addition of PAGE stop solution (20 µL) and denatured for 5 min

at 95 °C. Samples were separated with a 12.5% denaturing PAGE and visualized using fluorescence imaging (**Figure S10**).

Table S5. Reaction conditions specified for multiple nucleotide incorporation - four **dN^{R}TPs**, different template lengths.

template	Pwo (1U/µL) [µL]
Prb4basII (31-mer)	0.525
MO43 (43-mer)	0.525
MO61 (61-mer)	1
FVL-A (98-mer)	1
MO120 (120-mer)	1

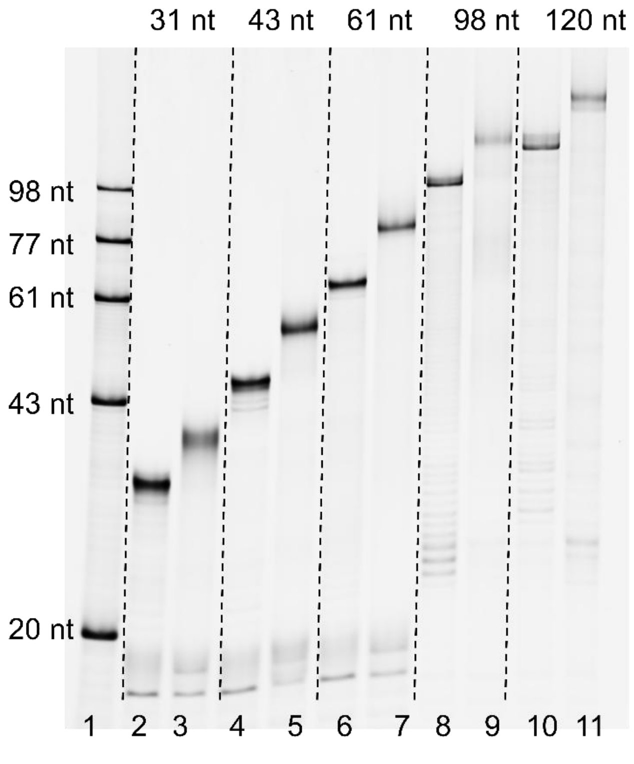

Figure S10. Denaturing PAGE analysis of PEX reactions using four different **dN^{R}TPs** (**dA^{PSH}TP**, **dC^{Im}TP**, **dU^{COOH}TP**, **dG^{OH}TP**) with different template length, lane (1) single-stranded ladder; lanes (2), (4), (6), (8) and (10) positive controls (dCTP, dTTP, dGTP and dATP); lanes (3), (5), (7), (9) and (11) hypermodified DNA.

2.1.7. Multiple incorporation (four modified dN^{R}TPs) – mixed PEX using protein-like and lipophilic dN^{R}TPs - 31-mer template - analytical scale

The reaction mixture (20 µL) contained 5'-(6-FAM)-labelled primer Prim248short (3 µM, 1 µL), template Prb4basII (3 µM, 1.5 µL), KOD XL DNA polymerase (0.5 U), set of four modified **dN^{R}TPs**, (**dC^{Im}TP**, **dU^{EPh}TP**, **dG^{AiPr}TP, dA^{PSH}TP**) (4 mM, 1 µL) and enzyme reaction buffer (10X, 2 µL) as supplied by the manufacturer. Negative controls were performed in absence of one modified or natural triphosphate of study. The reaction mixture was incubated for 30 min at 60 °C in a thermomixer, stopped by addition of PAGE stop solution (20 µL) and denatured for 5 min at 95 °C. Samples were separated with a 12.5% denaturing PAGE and visualized using fluorescence imaging (**Figure S11**).

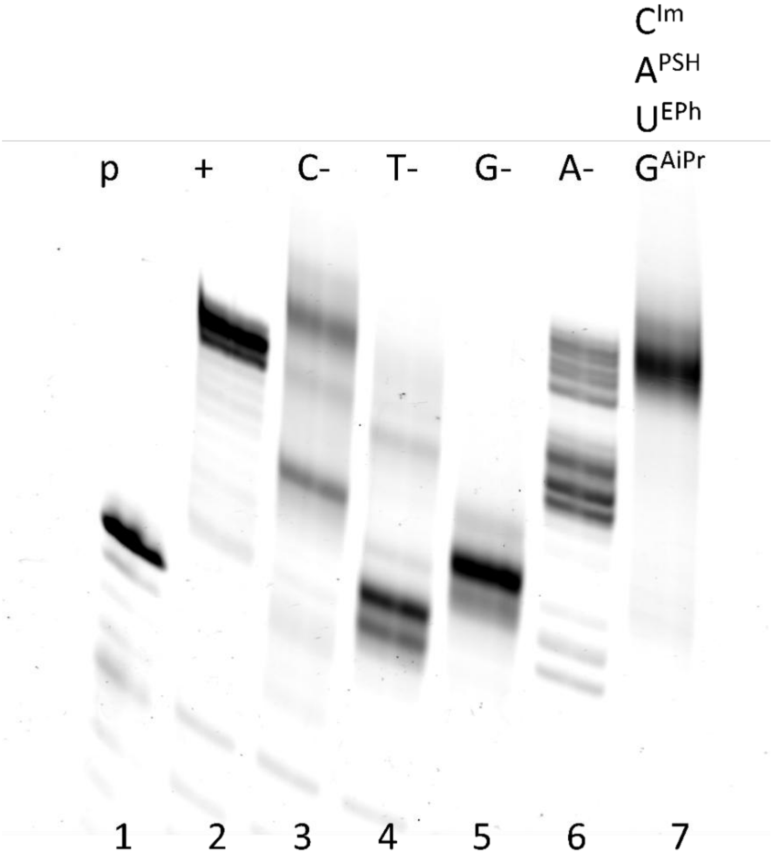

Figure S11. Denaturing PAGE analysis of PEX reaction with two protein-like (**dC^{Im}TP**, **dA^{PSH}TP**) and two lipophilic (**dU^{EPh}TP**, **dG^{AiPr}TP**) modified **dN^{R}TPs** using 31-mer template Prb4basII: lanes (1) primer; (2) positive control (dCTP, dTTP, dGTP and dATP); (3)-(6) negative controls in absence of modified or natural triphosphate of study dCTP, dTTP, dGTP or dATP; (7) **dC^{Im}TP**, **dU^{EPh}TP**, **dG^{AiPr}TP, dA^{PSH}TP**.

2.1.8. Multiple incorporation (four modified $dN^{R}TPs$) – mixed PEX using $dC^{Im}TP$, $dA^{PSH}TP$ and other two $dN^{R}TPs$ ($dU^{EPh}TP$, $dG^{AiPr}TP$) (two template lengths – analytical scale)

The reaction mixture (20 µL) contained 5'-(6-FAM)-labelled primer Prim248short (3 µM, 1 µL), template Prb4basII (31-mer) or MO61 (61-mer) (3 µM, 1.5 µL), KOD XL DNA polymerase (0.5 U), set of four modified **$dN^{R}TPs$** (**$dC^{Im}TP$**, **$dU^{EPh}TP$**, **$dG^{AiPr}TP$, $dA^{PSH}TP$**) (4 mM, 1 µL) and enzyme reaction buffer (10X, 2 µL) as supplied by the manufacturer. The reaction mixture was incubated for 30 min at 60 °C in a thermomixer, stopped by addition of PAGE stop solution (20 µL) and denatured for 5 min at 95 °C. Samples were separated with a 12.5% denaturing PAGE and visualized using fluorescence imaging (**Figure S12**).

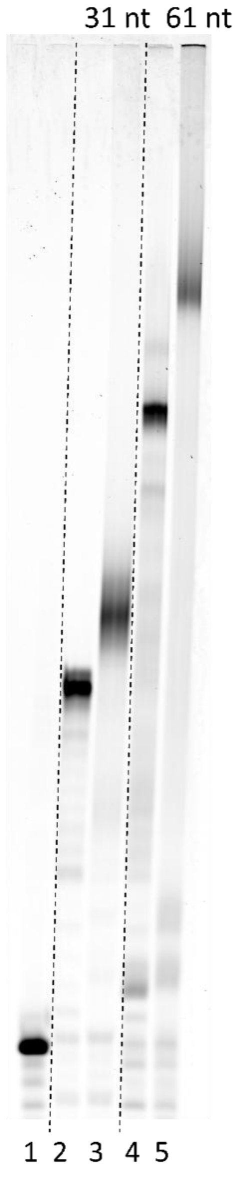

Figure S12. Denaturing PAGE analysis of PEX reactions using four different modifications (**dC^{Im}TP**, **dU^{EPh}TP**, **dG^{AiPr}TP, dA^{PSH}TP**) with different template length, lane (1) primer; lanes (2), (4) positive controls (dCTP, dTTP, dGTP and dATP); lanes (3), (5) hypermodified DNA.

2.2. General procedure for preparation of ONs for MALDI-TOF

In PEX reactions undergoing subsequent magnetoseparation, 5'-biotinylated template and 5'-(6-FAM)-labelled primer Prim248short were used. To obtain sufficient amount of modified single-stranded DNA, PEX reactions (in details see sections **2.1.1 – 2.1.8**) were five times scaled-up.

Method C (ssONs)

50 µL of streptavidin magnetic beads (SMB) were washed three times with 200 µL of Binding buffer TEN 100 (10 mM Tris, 1 mM EDTA, 100 mM NaCl, pH 7.5). The PEX reaction mixture was diluted to 50 µL with water, added to 100 µL of pre-washed SMB and incubated for 30 minutes (15 °C, 1400 rpm). SMB were captured on magnet (DynaMag-2, Invitrogen), washed three times with 200 µL of Washing buffer TEN 500 (10 mM Tris, 1 mM EDTA, 500 mM NaCl, pH 7.5) and three times with 200 µL of MilliQ water. Modified strand was then released by denaturation in 50 µL of hot water (incubation for 2 minutes, 75 °C, 900 rpm), SMB bearing template strand was quickly captured on magnet and water solution containing modified strand was taken out into a clean vial, evaporated, and sent for MALDI-TOF measurement. For results, see **Table S6**.

In PEX generating **19DNA_U^{COOH}**, non-biotinylated template and 5'-(6-FAM)-labelled primer Prim248short were used. The PEX reactions were also five times scaled-up (in details see section) and further purified using QIAquick Nucleotide Removal Kit according to manufacturer´s standard protocol. For results see **Table S6**.

2.3. MALDI-TOF measurments

Table S6. Overview of modified DNAs/ONs and their acquired masses

ON [a] name	Mw calculated [Da]	Mw found [Da]	Δ [Da]	Figure number
**19ON_C^{Im}**	6704.0	6705.3	1.3	**Figure S23**
**19DNA_U^{COOH}**	6613.0	6614.4	1.4	**Figure S24**
**19ON_A^{PSH}**	6661.2	6664.0	2.8	**Figure S25**
**19ON_A^{ASH}**	6613.0	6615.8	2.8	**Figure S26**
**19ON_A^{THT}**	6608.9	6612.9	4.0	**Figure S27**
**19ON_G^{OH}**	6534.0	6537.8	3.8	**Figure S28**
**31ON_C^{Im}**	11023.5	11024.3	0.8	**Figure S29**
**31ON_U^{COOH}**	10594.4	10595.7	1.3	**Figure S30**
**31ON_G^{OH}**	10478.7	10478.0	0.7	**Figure S31**
**31ON_A^{PSH}**	10751.3	10757.8	6.5	**Figure S32**
**31ON_A^{ASH}**	10557.9	10565.3	7.4	**Figure S33**
**31ON_A^{THT}**	10541.8	10545.0	3.2	**Figure S34**
**31ON_C^{Im}U^{COOH}A^{PSH}G^{OH}**	12385.3	12425.0	39.7	**Figure S35**
**31ON_C^{Im}U^{COOH}A^{ASH}G^{OH}**	12191.9	12255.4	63.5	**Figure S36**
**31ON_C^{Im}U^{COOH}A^{THT}G^{OH}**	12175.8	12216.3	40.5	**Figure S37**
**31ON_C^{Im}U^{EPh}A^{PSH}G^{AiPr}**	12239.4	12243.3	3.9	**Figure S38**

ON = single-stranded DNA; DNA = double-stranded; [a] = 5’-(6-FAM)-labeled

2.4. PCR

2.4.1. PCR – Single incorporation (one modified dN^{R}TP)

Reaction mixture (20 µL) contained FVL-A template (0.5 µM, 1 µL), 5’-(Cy5)-labelled L20 and 5’-(6-FAM)-labelled LT25TH primers (10 µM, 2 µL, each), natural dNTPs (4 mM, 1 µL), modified **dN^{R}TP** of study (4 mM, 1 µL), KOD XL DNA polymerase (0.5 U in case of **dC^{Im}TP**, **dU^{COOH}TP**, **dG^{OH}TP**, 1.5 U in case of **dA^{PSH}TP, dA^{THT}TP, dA^{ASH}TP**) and reaction buffer (10X, 2 µL) as supplied by the manufacturer. Positive control contained 0.5 U of KOD XL DNA polymerase and natural dNTPs (4 mM, 1 µL). Negative controls were performed in absence of the modified or

natural triphosphate of study. All reaction mixtures were under cycling protocol: 95 °C for 3 min, followed by 30 cycles at 95 °C for 1 min, 55 °C for 1 min, and 72 °C for 1 min, followed by a final elongation step at 75 °C for 5 min. Amplified products were analysed by agarose gel and visualised using fluorescence imaging (**Figure S13**).

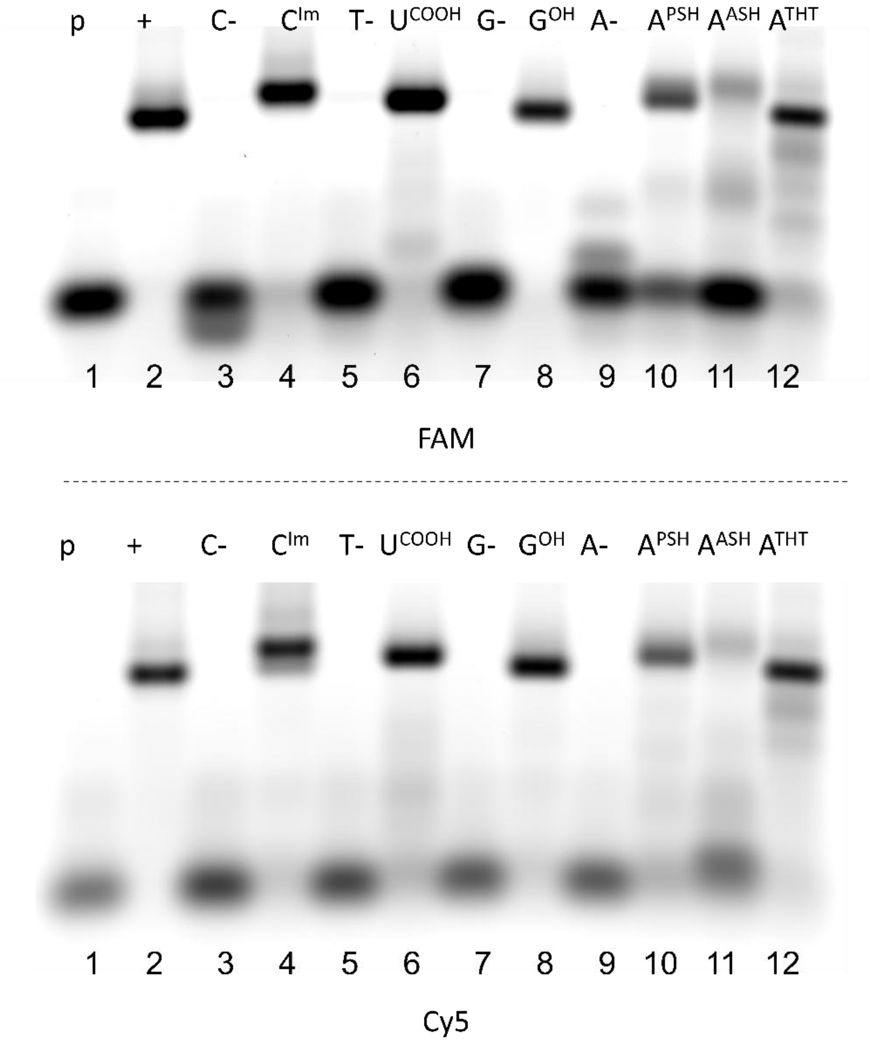

Figure S13. Native agarose gel analysis of PCR experiments with one modified **dN^{R}TP** using 5′-(6-FAM)-labelled LT25TH primer, 5′-Cy5-labelled L20 primer, 98-mer template FVL-A, and KOD XL DNA polymerase; lanes (1) primer; (2) positive control (dCTP, dTTP, dGTP and dATP); (4), (6), (8), (10)-(12) reactions using **dC^{Im}TP**, **dU^{COOH}TP**, **dG^{OH}TP**, **dA^{PSH}TP**, **dA^{ASH}TP** or **dA^{THT}TP** in combination with the other three natural dNTPs; (3), (5), (7), (9) negative controls in absence of modified or natural triphosphate of study dCTP, dTTP, dGTP or dATP

For testing the inhibitory effect of the thiol-modified **dN^{R}TPs** on the DNA polymerase in PCR reaction (due to possible cross-linking reaction with cystein residues), the reaction mixture (20 μL) contained FVL-A template (0.5 μM, 1 μL), 5'-(Cy5)-labelled L20 and 5'-(6-FAM)-labelled LT25TH primers (10 μM, 2 μL, each), set of four natural dNTPs (4 mM, 1 μL), thiol-modified **dN^{R}TP** (**dA^{PSH}TP** or **dA^{ASH}TP**) of study (4 mM, 1 μL), KOD XL DNA polymerase (1.5 U) and

reaction buffer (10X, 2 μL) as supplied by the manufacturer. Positive control contained 0.5 U of KOD XL DNA polymerase and natural dNTPs (4 mM, 1 μL). All reaction mixtures were under cycling protocol: 95 °C for 3 min, followed by 30 cycles at 95 °C for 1 min, 55 °C for 1 min, and 72 °C for 1 min, followed by a final elongation step at 75 °C for 5 min. Amplified products were analysed by native agarose gel and visualised using fluorescence imaging (**Figure S14**).

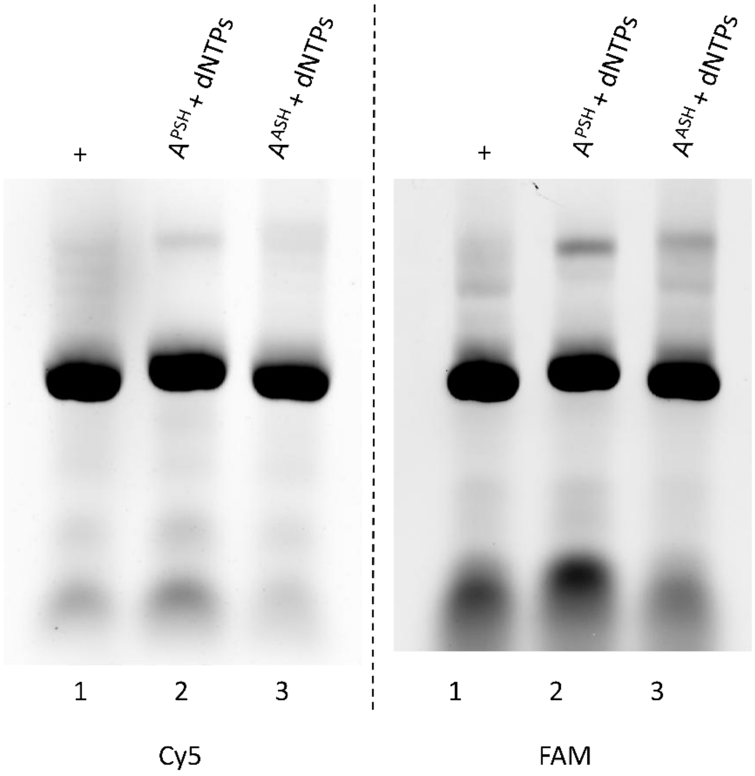

Figure S14. Native agarose gel analysis of PCR experiments with thiol-modified **dN^{R}TP** (**dA^{PSH}TP** or **dA^{ASH}TP**) and a set of four natural dNTPs using 5′-(6-FAM)-labelled LT25TH primer, 5′-Cy5-labelled L20 primer, 98-mer template FVL-A, and KOD XL DNA polymerase; lane (1) positive control (dCTP, dTTP, dGTP and dATP); (2) **dA^{PSH}TP**, dCTP, dTTP, dGTP and dATP; (3) **dA^{ASH}TP**, dCTP, dTTP, dGTP and dATP.

2.4.2. PCR – Multiple incorporation (two modified dN^{R}TP)

Reaction mixture (20 μL) contained FVL-A template (0.5 μM, 1 μL), 5’-(Cy5)-labelled L20 and 5’-(6-FAM)-labelled LT25TH primers (10 μM, 2 μL, each), natural dNTPs (4 mM, 1 μL), combination of two **dN^{R}TPs** (4 mM, 1 μL, each), KOD XL DNA polymerase (0.5 U for combinations without **dA^{PSH}TP (Figure S15A)** or 2.5 U for combinations with **dA^{PSH}TP** (**Figure S15B**)) and reaction buffer (10X, 2 μL) as supplied by the manufacturer. Positive control contained 0.5 U of KOD XL DNA polymerase and natural dNTPs (4 mM, 1 μL). Negative controls were performed in absence of one modified or natural triphosphate of study. All reaction mixtures were under cycling protocol: 95 °C for 3 min, followed by 30 cycles at 95 °C for 1 min, 55 °C for 1

min, and 72 °C for 1 min, followed by a final elongation step at 75 °C for 5 min. Amplified products were analysed by native agarose gel using fluorescence imaging (**Figure S13**).

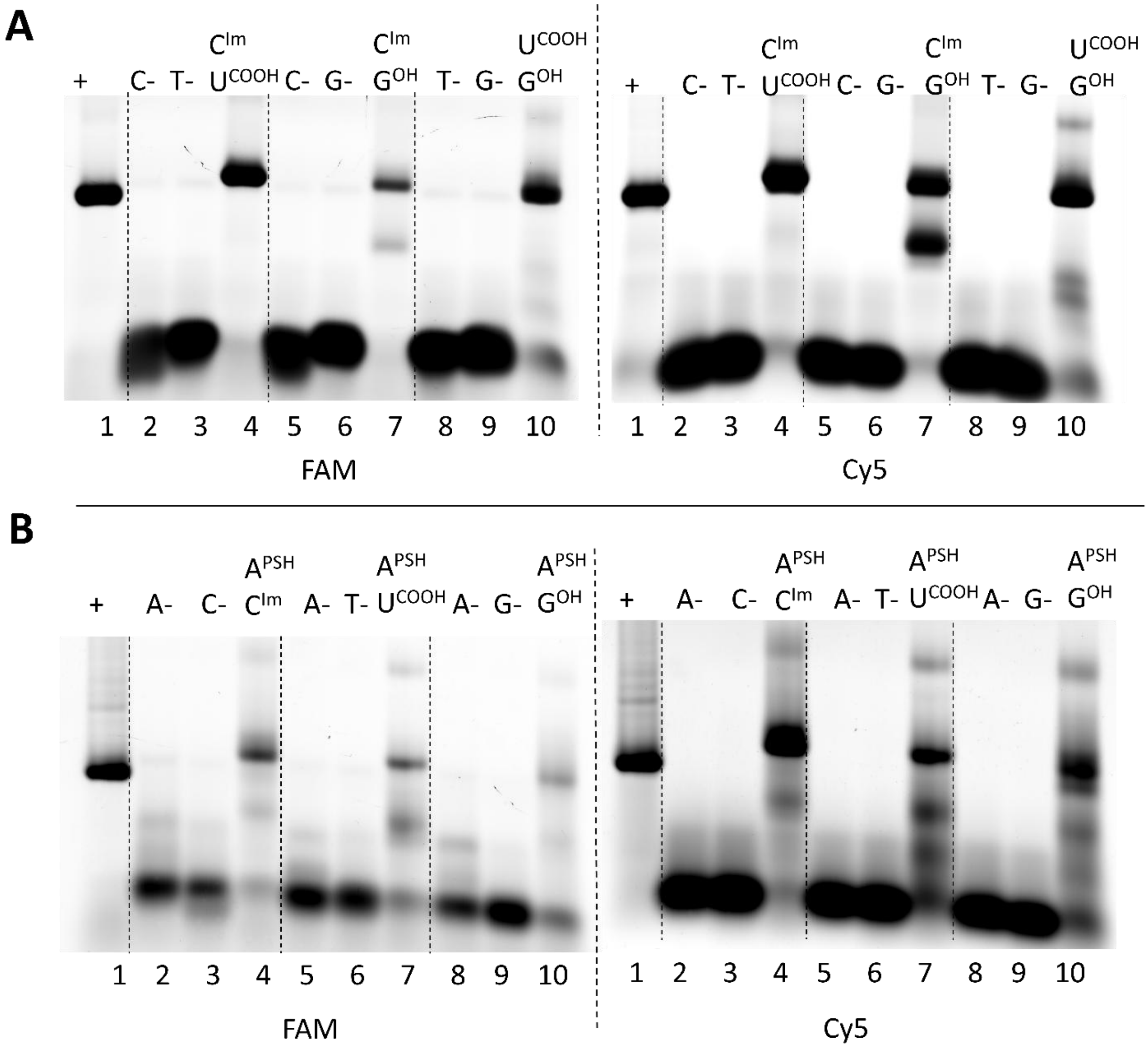

Figure S15. Native agarose gel analysis of PCR experiments of combinations of one two **dN^{R}TPs** using 5′-(6-FAM)-labelled LT25TH primer, 5′-Cy5-labelled L20 primer, 98-mer template FVL-A and KOD XL DNA polymerase; (**A**): lanes (1) positive control (dCTP, dTTP, dGTP and dATP); (2) **dU^{COOH}TP**, dATP, dGTP; (3) **dC^{Im}TP**, dGTP, dATP; (4) **dC^{Im}TP**, **dU^{COOH}TP**, dATP, dGTP; (5) **dG^{OH}TP**, dATP, dTTP; (6) **dC^{Im}TP**, dATP, dTTP; (7) **dC^{Im}TP**, **dG^{OH}TP**, dATP, dTTP; (8) **dG^{OH}TP**, dATP, dCTP; (9) **dU^{COOH}TP**, dATP, dCTP; (10) **dU^{COOH}TP**, **dG^{OH}TP**, dATP, dCTP. (**B**): lanes (1) positive control (dCTP, dTTP, dGTP and dATP); (2) **dC^{Im}TP**, dTTP, dGTP; (3) **dA^{PSH}TP**, dTTP, dGTP; (4) **dA^{PSH}TP, dC^{Im}TP**, dTTP, dGTP; (5) **dU^{COOH}TP**, dGTP, dCTP; (6) **dA^{PSH}TP**, dGTP, dCTP; (7) **dA^{PSH}TP, dU^{COOH}TP**, dGTP, dCTP; (8) **dG^{OH}TP**, dCTP, dTTP; (9) **dA^{PSH}TP**, dCTP, dTTP; (10) **dA^{PSH}TP**, **dG^{OH}TP**, dCTP, dTTP.

2.4.3. PCR – Multiple incorporation (three modified dN^{R}TPs)

Reaction mixture (20 µL) contained FVL-A template (0.5 µM, 1 µL), 5'-(Cy5)-labelled L20 and 5'-(6-FAM)-labelled LT25TH primers (10 µM, 2 µL, each), natural dNTPs (4 mM, 1 µL), combination of three **dN^{R}TPs** (**Figure S16A**, for combinations including **dA^{PSH}TP**, see **Figure S16B**) (4 mM, 1 µL), KOD XL DNA polymerase (2.5 U) and reaction buffer (10X, 2 µL) as supplied by the manufacturer. Positive control contained 0.5 U of KOD XL DNA polymerase and natural dNTPs (4 mM, 1 µL). Negative controls were performed in absence of one modified or natural triphosphate of study. All reaction mixtures were under cycling protocol: 95 °C for 3 min, followed by 30 cycles at 95 °C for 1 min, 55 °C for 1 min, and 72 °C for 1 min, followed by a final elongation step at 75 °C for 5 min. Amplified products were analysed by native agarose gel using fluorescence imaging.

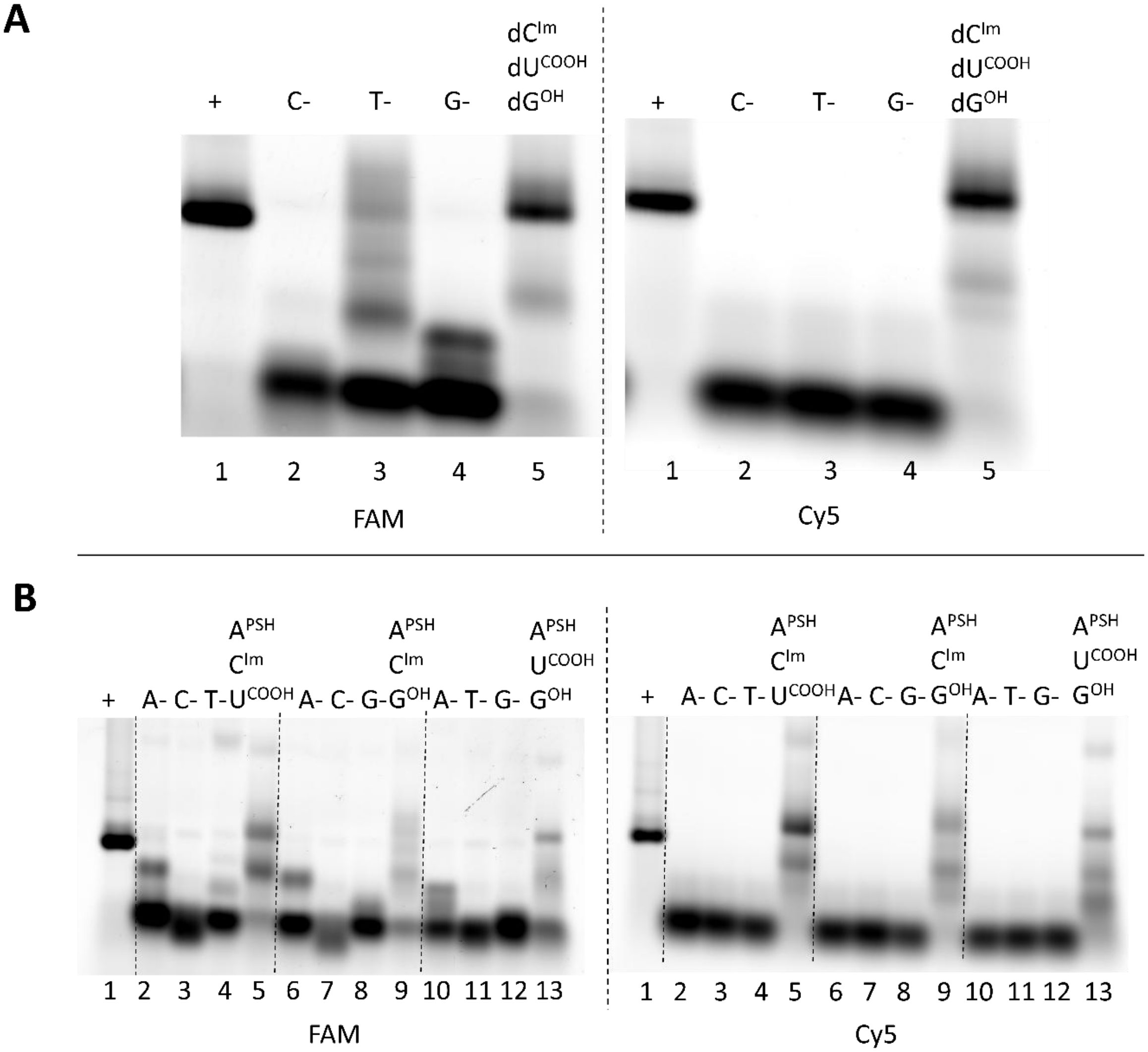

Figure S16. Native agarose gel analysis of PCR experiments of combinations of three **dN^{R}TPs** using 5′-(6-FAM)-labelled LT25TH primer, 5′-Cy5-labelled L20 primer, 98-mer template FVL-A, and KOD XL DNA polymerase. **A:** lane (1) positive control (dCTP, dTTP, dGTP and dATP); (2) dATP, **dU^{COOH}TP**, **dG^{OH}TP**; (3) dATP, **dG^{OH}TP**, **dC^{Im}TP** (4), dATP, **dU^{COOH}TP**, **dC^{Im}TP;** (5) **dC^{Im}TP**, **dU^{COOH}TP**, **dG^{OH}TP**, dATP. **B:** lanes (1) positive control (dCTP, dTTP, dGTP and dATP); (2) **dU^{COOH}TP**, **dC^{Im}TP**, dGTP; (3) **dA^{PSH}TP**, **dU^{COOH}TP**, dGTP; (4) **dA^{PSH}TP**, **dC^{Im}TP**, dGTP; (5) **dA^{PSH}TP**, **dC^{Im}TP**, **dU^{COOH}TP**, dGTP; (6) **dC^{Im}TP**, **dG^{OH}TP**, dTTP; (7) **dA^{PSH}TP**, **dG^{OH}TP**, dTTP; (8) **dC^{Im}TP**, **dA^{PSH}TP**, dTTP; (9) **dA^{PSH}TP**, **dC^{Im}TP**, **dG^{OH}TP**, dTTP; (10) **dU^{COOH}TP**, **dG^{OH}TP**, dCTP; (11) **dA^{PSH}TP**, **dG^{OH}TP**, dCTP; (12) **dA^{PSH}TP**, **dU^{COOH}TP**, dCTP; (13) **dA^{PSH}TP**, **dU^{COOH}TP**, **dG^{OH}TP**, dCTP.

2.4.4. PCR – Multiple incorporation (four modified dN^{R}TPs)

Reaction mixture (20 µL) contained FVL-A template (0.5 µM, 1 µL), 5’-(Cy5)-labelled L20 and 5’-(6-FAM)-labelled LT25TH primers (10 µM, 2 µL, each), modified **dN^{R}TP** of study (**dC^{Im}TP**, **dU^{COOH}TP**, **dG^{OH}TP, dA^{PSH}TP**) (4 mM, 1 µL), KOD XL DNA polymerase (from 0.5 U to 5 U) and reaction buffer (10X, 2 µL) as supplied by the manufacturer. Positive control contained 0.5 U of KOD XL DNA polymerase and natural dNTPs (4 mM, 1 µL). All reaction mixtures were under cycling protocol: 95 °C for 2 min, followed by 30 cycles at 95 °C for 1 min, 55 °C for 1 min, and 72 °C for 1 min, followed by a final elongation step at 75 °C for 5 min. The reactions were analysed by native agarose gel and visualised using fluorescence imaging (**Figure S17**).

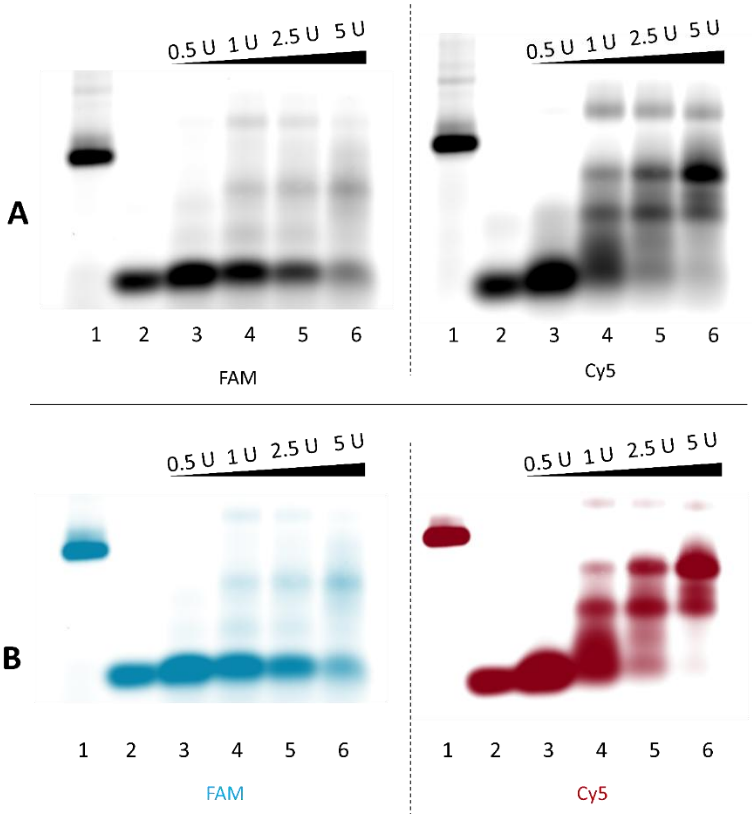

Figure S17. Native agarose gel analysis of PCR experiments with four modified **dN^{R}TPs** (**dC^{Im}TP**, **dU^{COOH}TP**, **dG^{OH}TP, dA^{PSH}TP**) using 5′-(6-FAM)-labelled LT25TH primer, 5′-Cy5-labelled L20 primer, 98-mer template FVL-A and KOD XL DNA polymerase; **A** corresponds to unadjusted agarose gel scan, **B** corresponds to adjusted scan image for each label; (1) positive control – natural dNTPs; (2) primers PCR products using (3) 0.5 U; (4) 1 U; (5) 2.5 U and (6) 5 U of KOD XL DNA polymerase. Visualization: **A** and **B**) FAM scan (left and in blue), Cy5 scan (right and in red).

2.5. aPCR (four modified dN^{R}TPs)

Reaction mixture (20 µL) contained template FVL-A-3Cs (5 µM, 1 µL), 5′-(6-FAM)-labelled Flank_LT25TH primer (10 µM, 2 µL,), modified **dN^{R}TP (dC^{Im}TP**, **dU^{COOH}TP**, **dG^{OH}TP, dA^{PSH}TP)** (4 mM, 1 µL, each), KOD XL DNA polymerase (2.5 or 5 U) and reaction buffer (10X, 2 µL) as supplied by the manufacturer. All reaction mixtures were under cycling protocol: 95 °C for 2 min, followed by either 30 or 50 cycles at 95 °C for 1 min, 55 °C for 1 min, and 72 °C for 1 min, followed by a final elongation step at 75 °C for 5 min. Samples were analysed by PAGE and visualised using fluorescence imaging (**Figure S18**). The product **118ON_C^{Im}U^{COOH}A^{PSH}G^{OH}** was purified using AMPure XP magnetic particles according to the protocol (**Figure S19**).

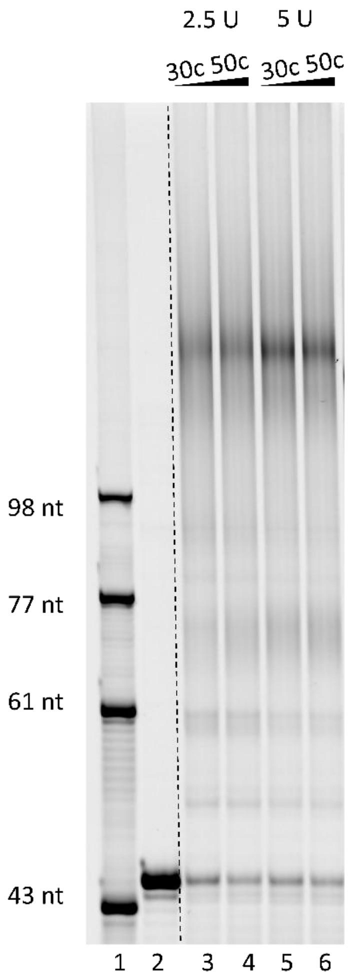

Figure S18. Denaturing PAGE of aPCR with four **dN^{R}TPs** (**dC^{Im}TP**, **dU^{COOH}TP**, **dG^{OH}TP, dA^{PSH}TP**), lane (1): single-stranded ladder; (2) primer; (3) 2.5 U of KOD XL, 30 cycles; (4) 2.5 U of KOD XL, 50 cycles (5): 5 U of KOD XL, 30 cycles; (6): 5 U of KOD XL, 50 cycles.

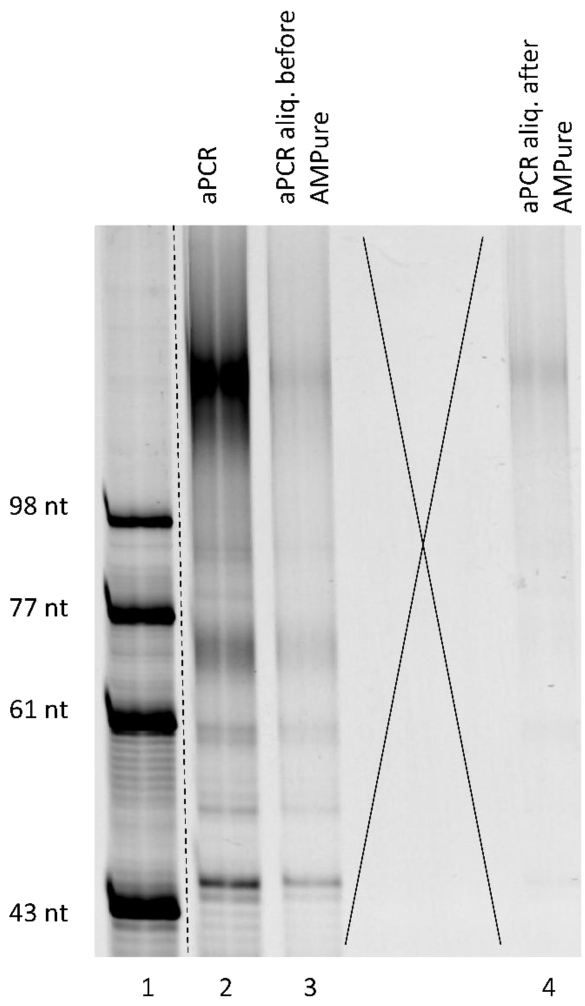

Figure S19. Denaturing PAGE of aPCR with four **dN^{R}TPs** (**dC^{Im}TP**, **dU^{COOH}TP**, **dG^{OH}TP, dA^{PSH}TP**) before and after purification using AMPure XP magnetic particles, lane (1): single-stranded ladder; (2) aPCR reaction; (3) aPCR reaction aliquot before purification; (4) same aPCR reaction aliquot after purification

2.6. rePCR

Reaction mixture (20 µL) contained 5′-(6-FAM)-labelled **118ON_C^{Im}U^{COOH}A^{PSH}G^{OH}** (fully modified ON product of aPCR) as template (0.4 µM, 4 µL), 5′-(6-FAM)-labelled L20 and 5′-(Cy5)-labelled Flank primer (10 µM, 2 µL, each), natural dNTPs (4 mM, 1 µL), KOD XL DNA polymerase (2.5 U) and reaction buffer (10X, 2 µL) as supplied by the manufacturer. All reaction mixtures were under cycling protocol: 95 °C for 2 min, followed by 30 cycles at 95 °C for 1 min, 55 °C for 1 min, and 72 °C for 1 min, followed by a final elongation step at 75 °C for 5 min. Samples were analyzed by PAGE and visualised using fluorescence imaging and purified from shorter side products by Agencourt AMPure XP magnetic particles according to the manufacturer's protocol (**Figure S20**).

For preparation of dsDNA for sequencing, **118rePCR** was prepared according to the same protocol as above but with non-labelled primers.

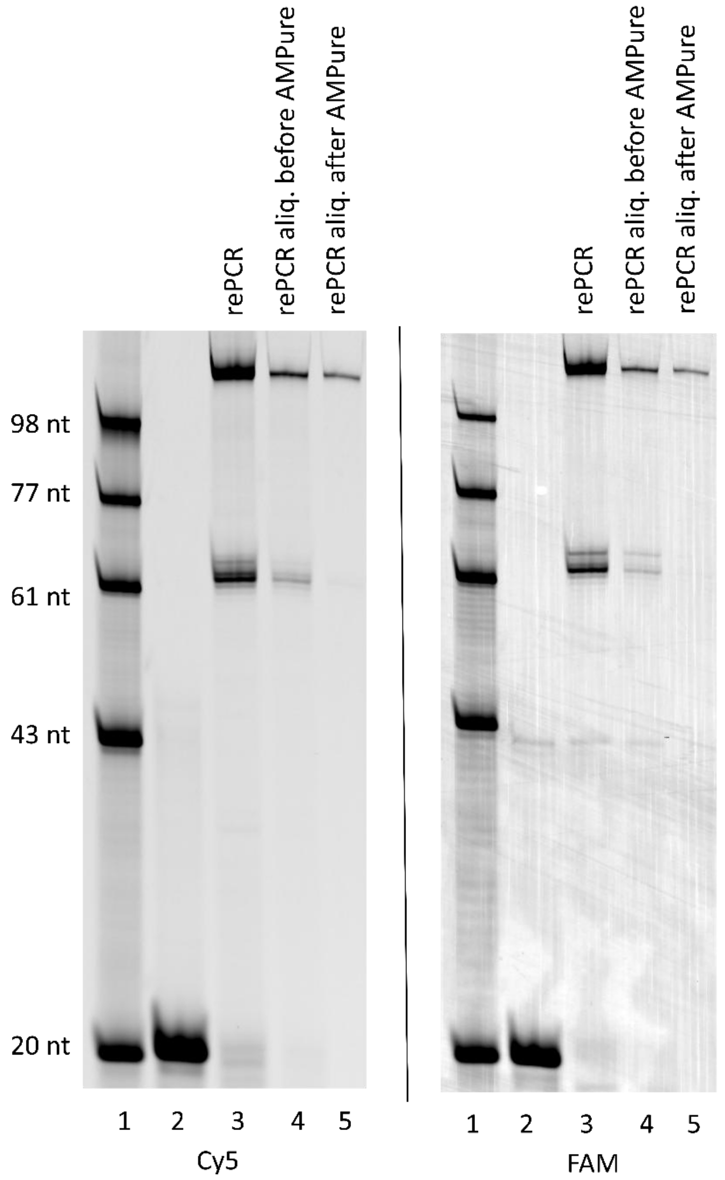

Figure S20. Denaturing PAGE of rePCR with natural dNTPs, lane (1): single-stranded ladder; (2): primer; (3): rePCR reaction; (4) rePCR reaction aliquot before purification; (5) same rePCR reaction aliquot after purification. Visualisation: Cy5 scan (left), FAM scan (right).

2.7. Sanger sequencing

In order to prepare dsDNA for Sanger sequencing, **118rePCR** was obtained using **118ON_C$^{\mathbf{Im}}$U$^{\mathbf{COOH}}$A$^{\mathbf{PSH}}$G$^{\mathbf{OH}}$** as template by the procedure described in section **2.6** (rePCR were

performed with non-labelled primers). Samples were purified by Agencourt AMPure XP magnetic particles according to the manufacturer’s protocol. Resulting natural **118rePCR** DNA sample (40 ng) was sent for Sanger sequencing using corresponding primers (5 µL, 5 µM). Primers L20Seq+ and Flank Seq+ (see **Table S1**) were used in order to improve sequencing results (**Figure S21**).

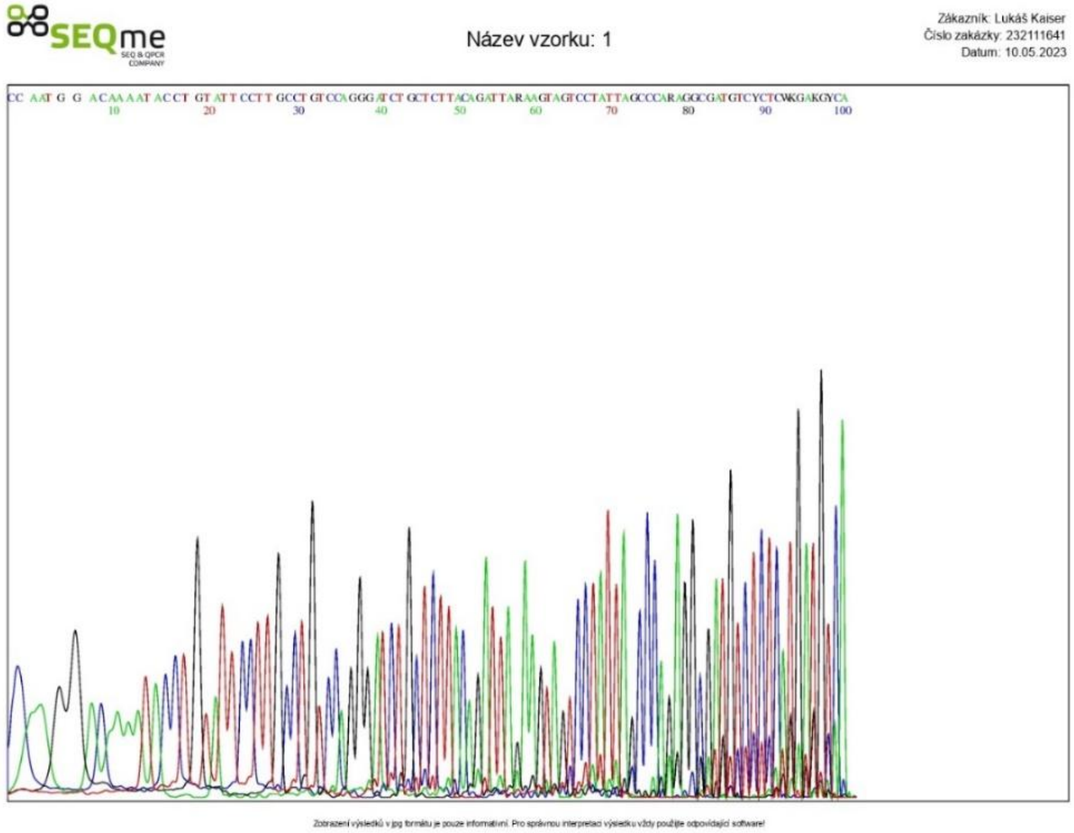

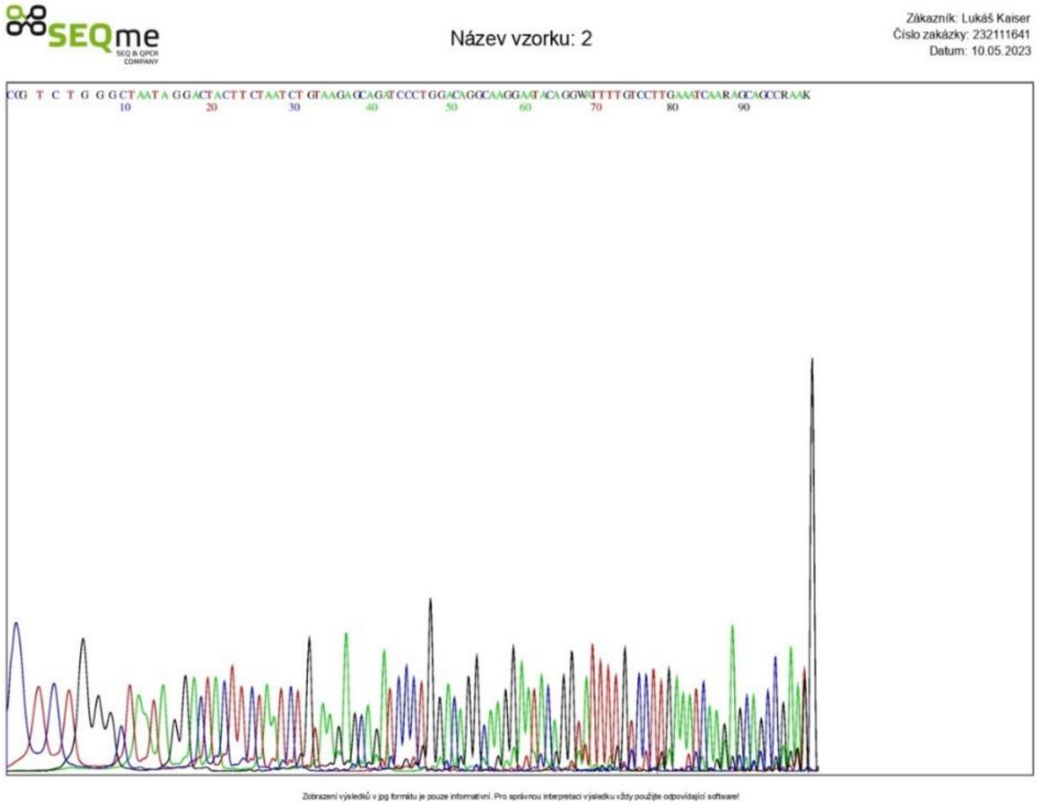

Figure S21. Sanger sequencing results. Sequencing chromatogram generated from **118rePCR** using reverse L20 Seq+ primer (upper) and forward Flank Seq+ primer (down).

2.8. Redox study of 98PCR_A^{PSH}

98PCR_A^{PSH} (see part **2.4.1.**) was purified using QIAquick PCR Purification Kit and left at room temperature for 48 hours. Then, **98PCR_A^{PSH}** (1 uM, 2 uL) was mixed either with TCEP·HCl (20 mM, 1 uL) or DTT (20 mM, 1 uL) or mix DTT/NaOH (20 mM, conc NaOH?? 1 uL) with addition of NaOH (10 mM, 2 uL), dissolved in water in total volume of 20 uL and incubated for 2 hours at room temperature. Samples were analyzed by native agarose gel and visualized using fluorescence imaging (**Figure S22**)

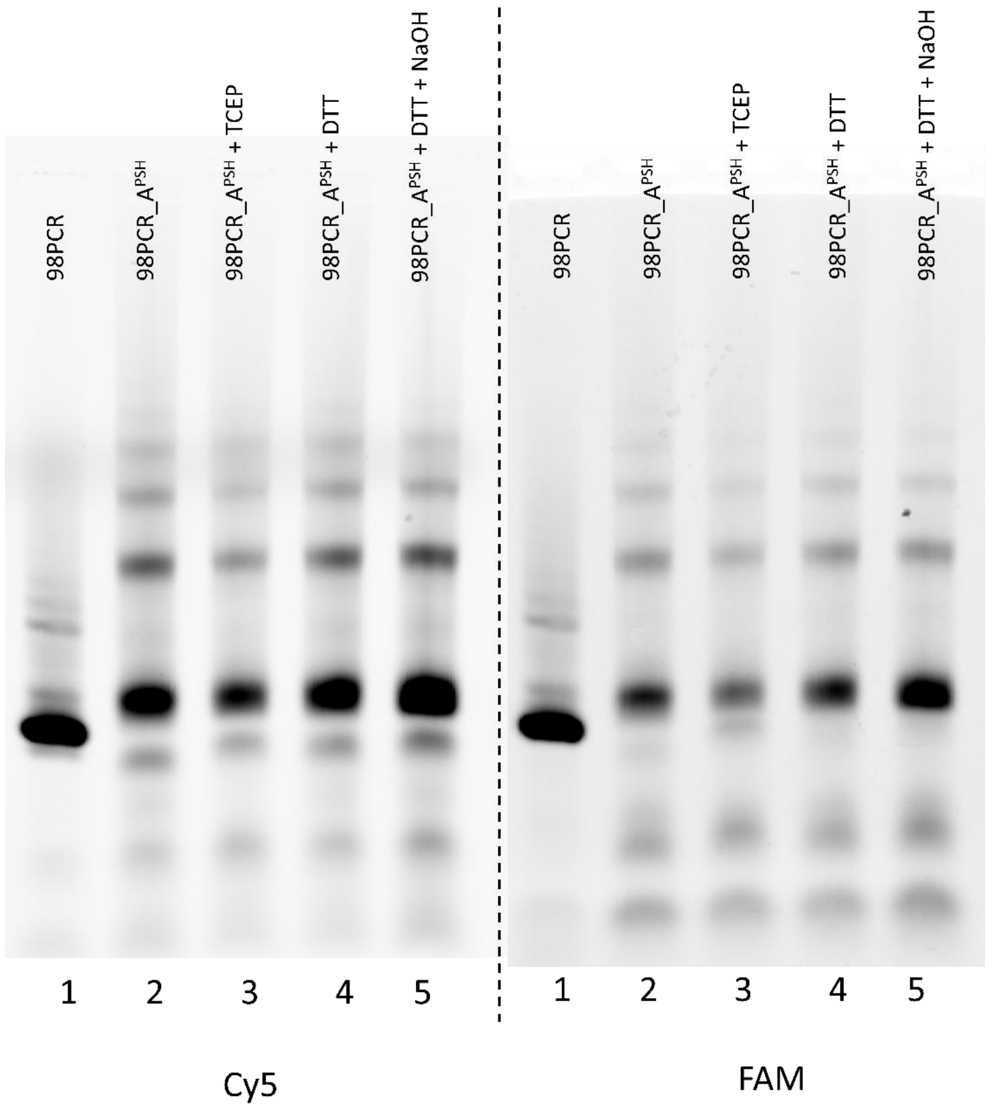

Figure S22. Redox study of **98PCR_A^{PSH}**, lane (1) positive control 98PCR purified by QIAquick PCR Purification Kit**;** (2) **98PCR_A^{PSH}** purified by QIAquick PCR Purification Kit**;** (3) purified **98PCR_A^{PSH}** treated with TCEP·HCl; (4) purified **98PCR_A^{PSH}** treated with DTT; (5) purified **98PCR_A^{PSH}** treated with DTT with addition of NaOH

3. Copies of MALDI-TOF mass spectra

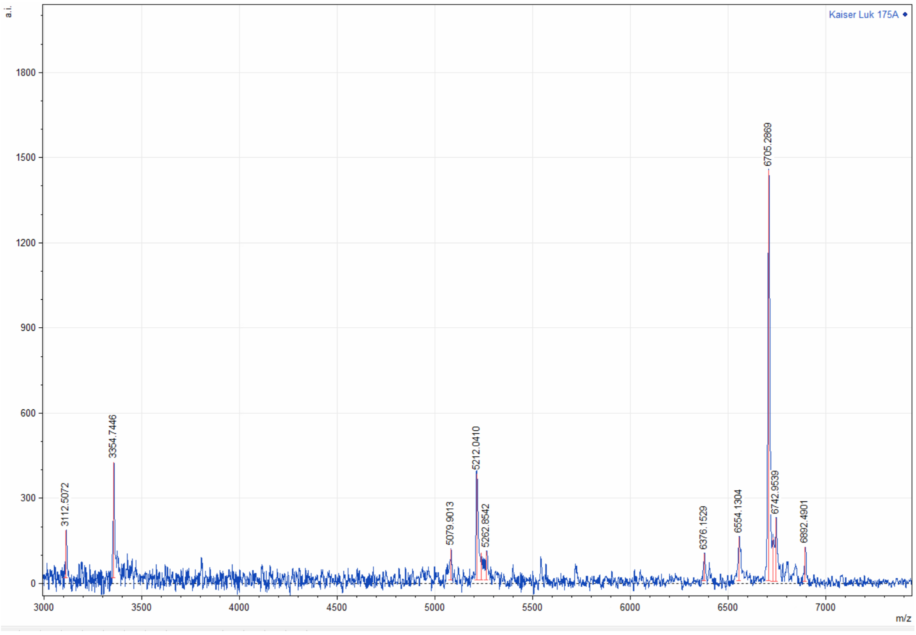

Figure S23. MALDI-TOF spectrum of **19ON_C$^{\mathbf{Im}}$**: calculated 6704.0 Da; found 6705.3 Da; Δ = 1.3 Da

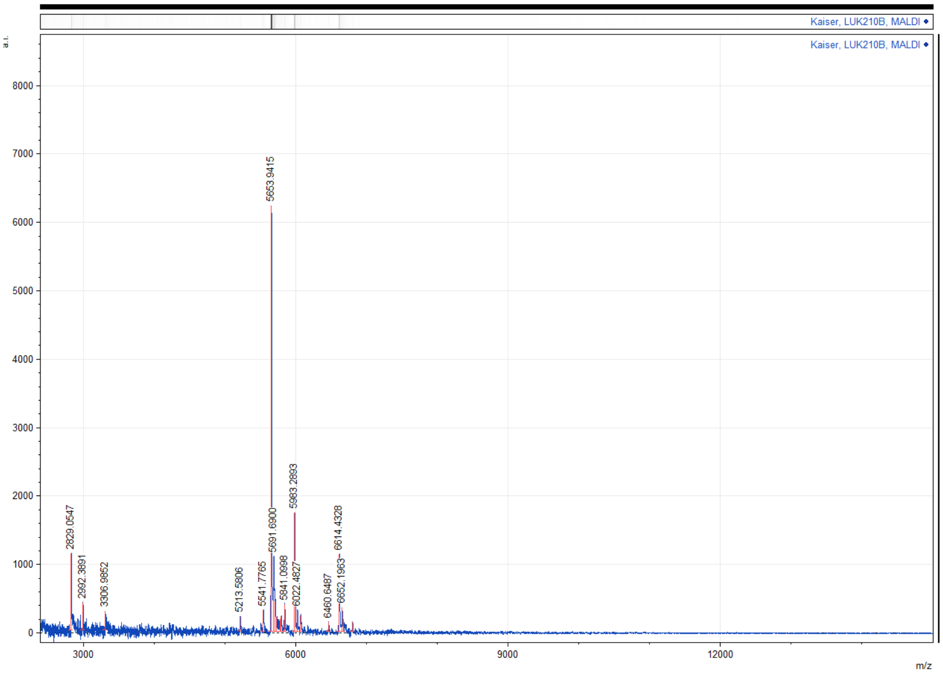

Figure S24. MALDI-TOF spectra of **19DNA_U$^{\mathbf{COOH}}$**: calculated 6613.0 Da; found 6614.4 Da Da; Δ = 1.4 Da

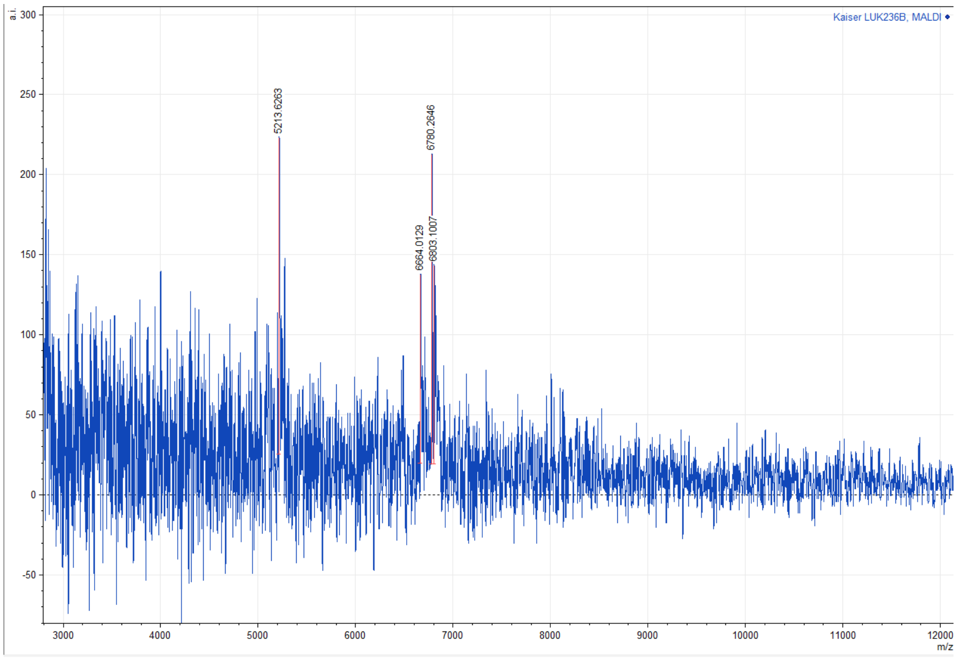

Figure S25. MALDI-TOF spectra of **19ON_A^{PSH}**: calculated 6661.2 Da; found 6664.0 Da; Δ = 2.8 Da

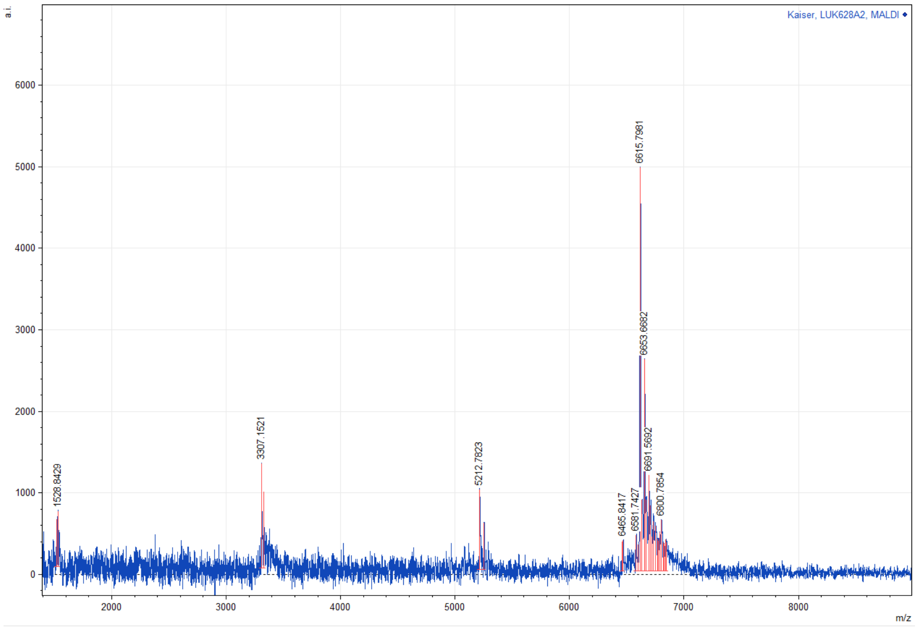

Figure S26. MALDI-TOF spectra of **19ON_A^{ASH}**: calculated 6613.0 Da; found 6615.8 Da; Δ = 2.8 Da

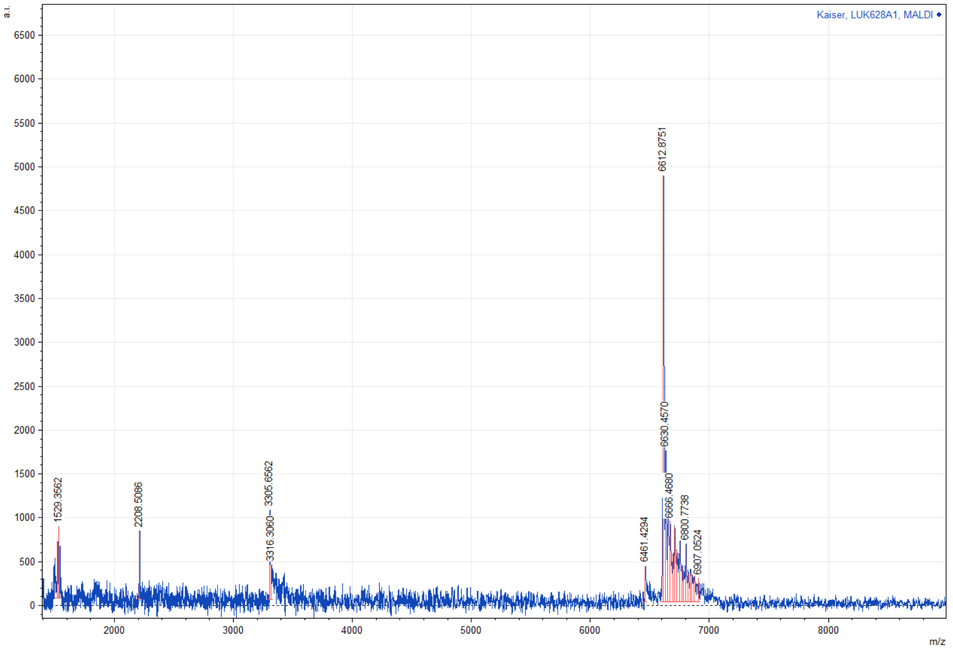

Figure S27. MALDI-TOF spectra of **19ON_A$^{\mathbf{THT}}$**: calculated 6608.9 Da; found 6612.9 Da; Δ = 4.0 Da

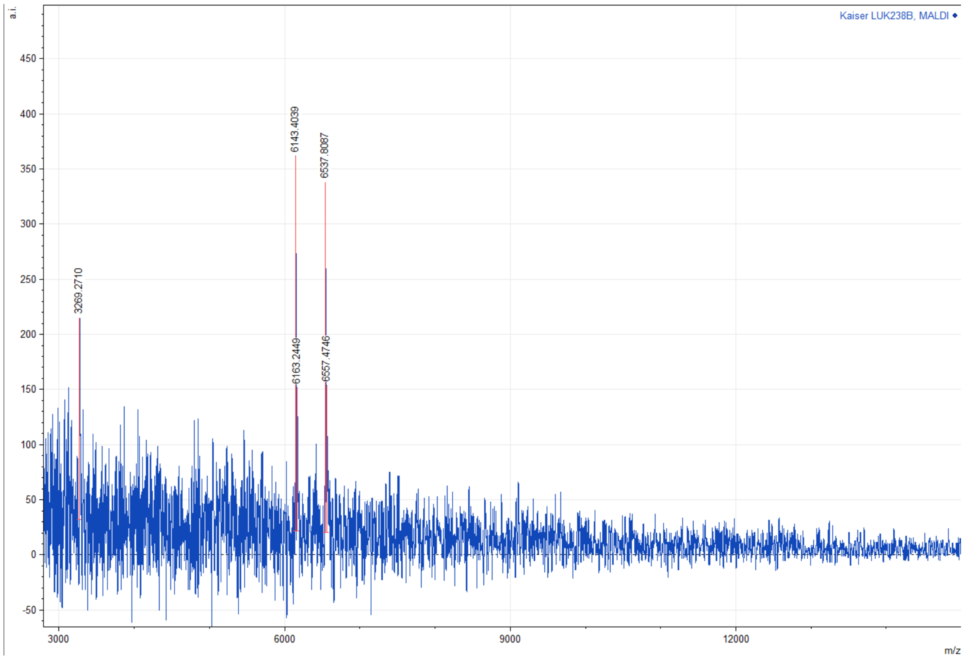

Figure S28. MALDI-TOF spectra of **19ON_G$^{\mathbf{OH}}$**: calculated 6534.0 Da; found 6537.8 Da; Δ = 3.8 Da

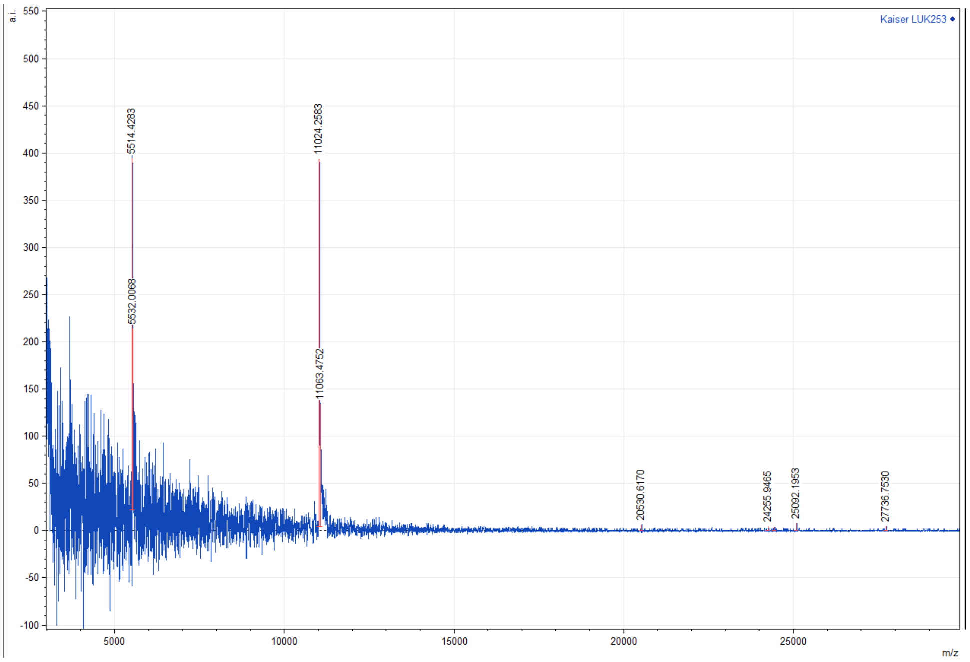

Figure S29. MALDI-TOF spectra of **31ON_C^{Im}**: calculated 11023.5 Da; found 11024.3 Da; Δ = 0.8 Da

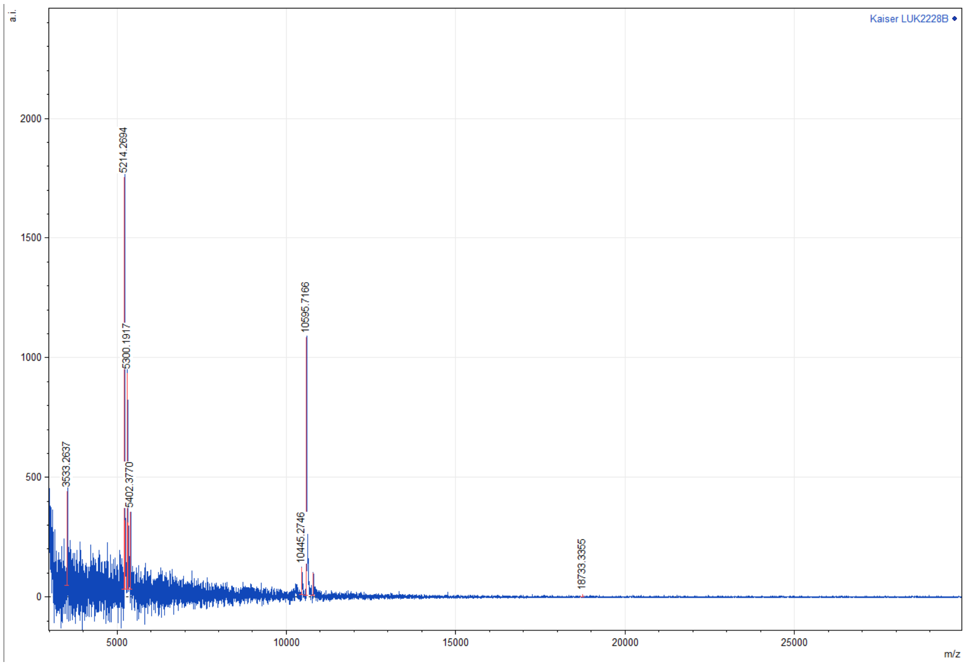

Figure S30. MALDI-TOF spectra of **31ON_U^{COOH}**: calculated 10594.4 Da; found 10595.7 Da; Δ = 1.3 Da

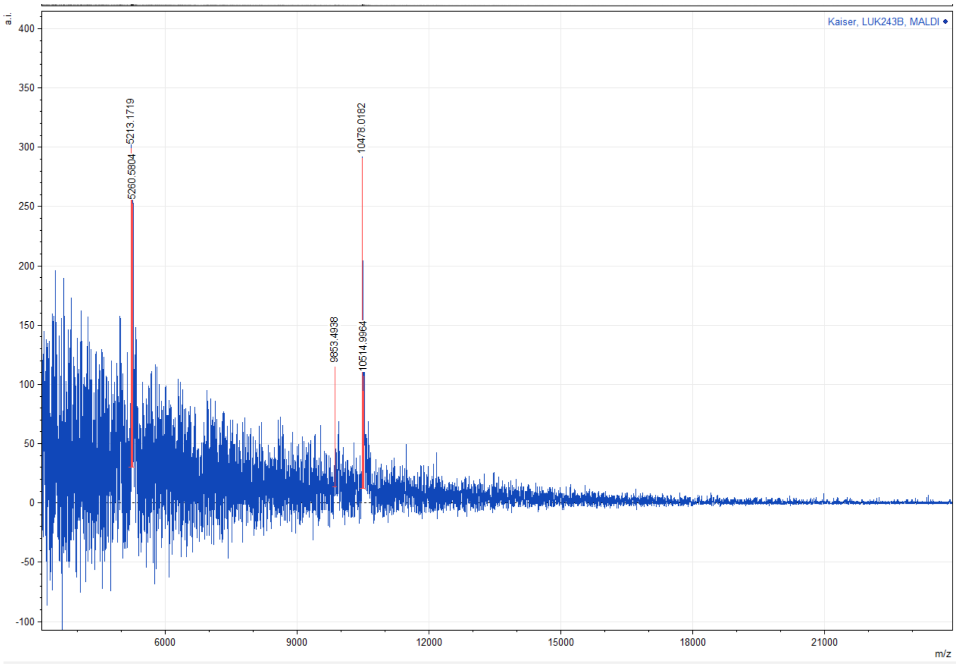

Figure S31. MALDI-TOF spectra of **31ON_G^{OH}**: calculated 10478.7 Da; found 10478.0 Da; Δ = 0.7 Da

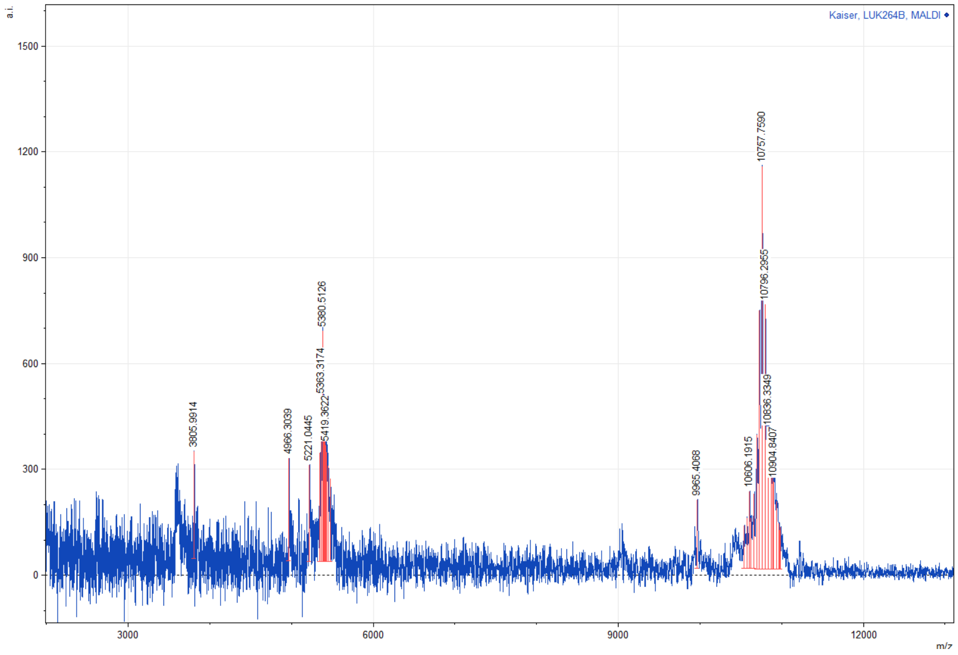

Figure S32. MALDI-TOF spectra of **31ON_A^{PSH}**: calculated 10751.3 Da; found 10757.8 Da; Δ = 6.5 Da

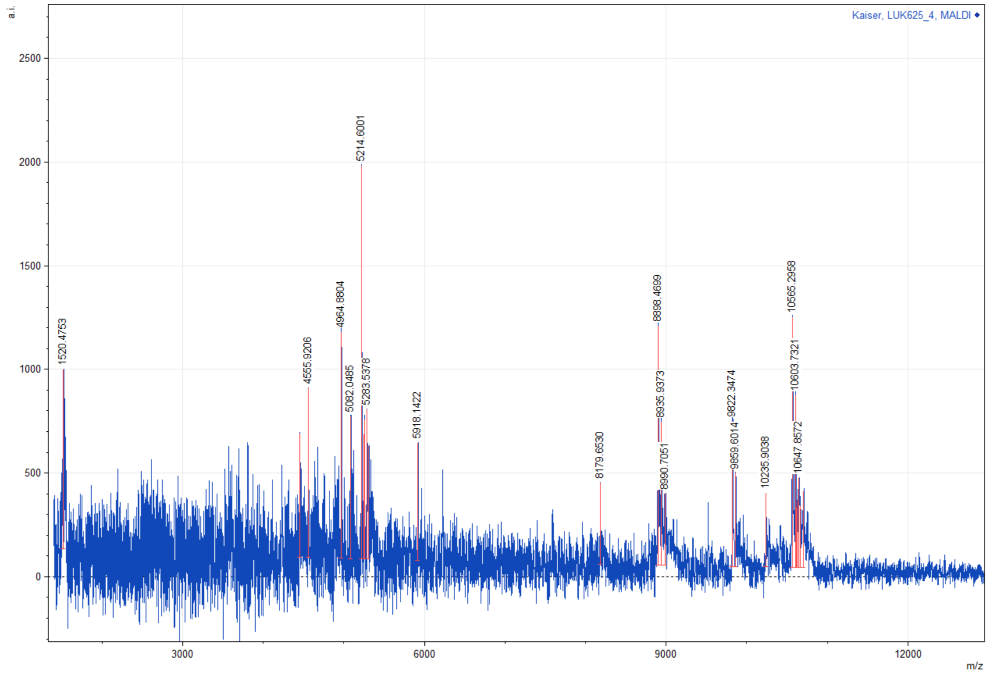

Figure S33. MALDI-TOF spectra of **31ON_A$^{\mathbf{ASH}}$**: calculated 10.557.9; found 10565.3 Da; Δ = 7.4 Da

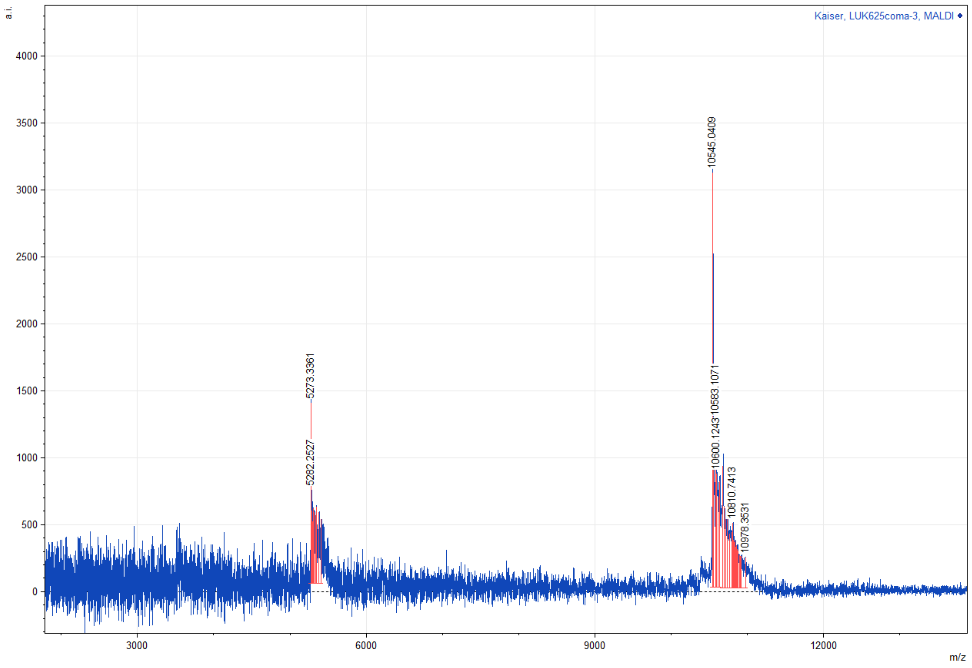

Figure S34. MALDI-TOF spectra of **31ON_A$^{\mathbf{THT}}$**: calculated 10541.8 Da; found 10545.0 Da; Δ = 3.2 Da

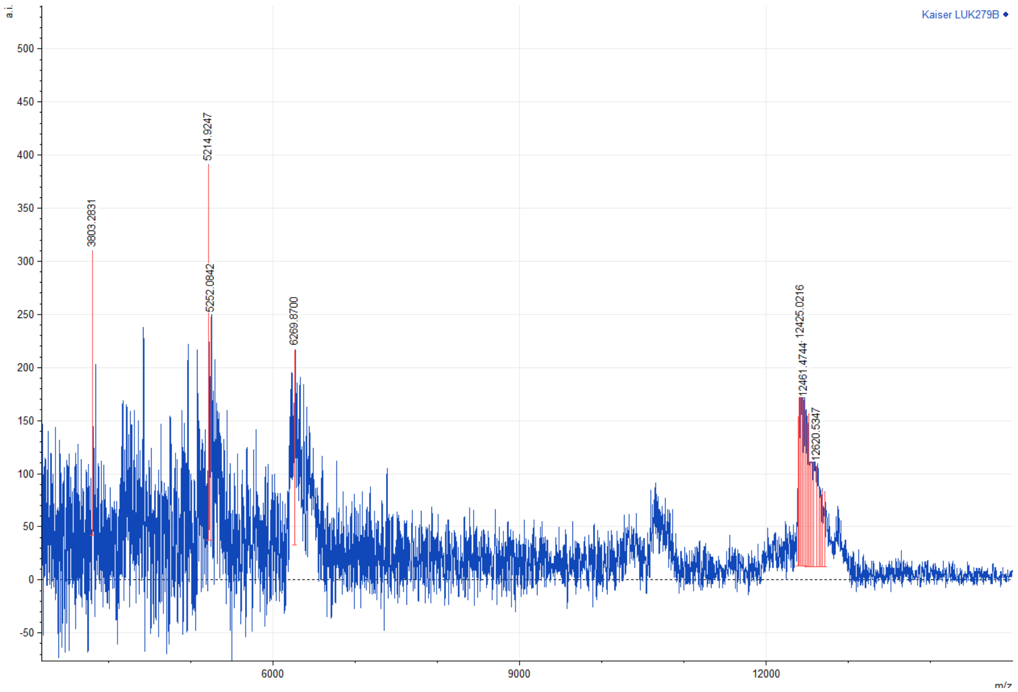

Figure S35. MALDI-TOF spectra of **31ON_C^{Im}U^{COOH}A^{PSH}G^{OH}**: calculated 12385.3 Da; found 12425.0 Da; Δ = 39.7 Da

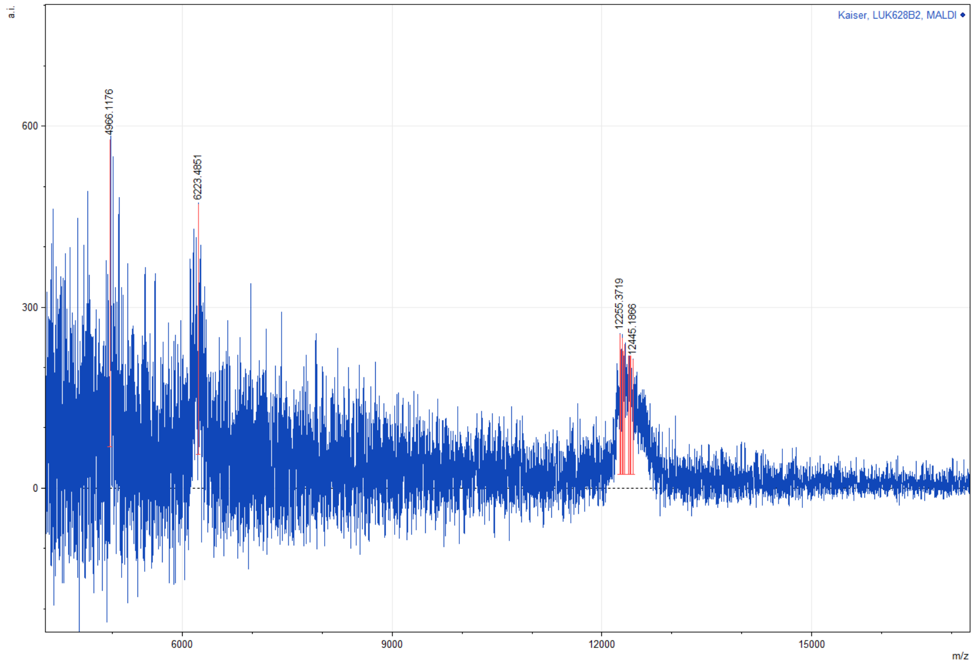

Figure S36. MALDI-TOF spectra of **31ON_C^{Im}U^{COOH}A^{ASH}G^{OH}**: calculated 12191.9 Da; found 12255.4 Da; Δ = 63.5 Da

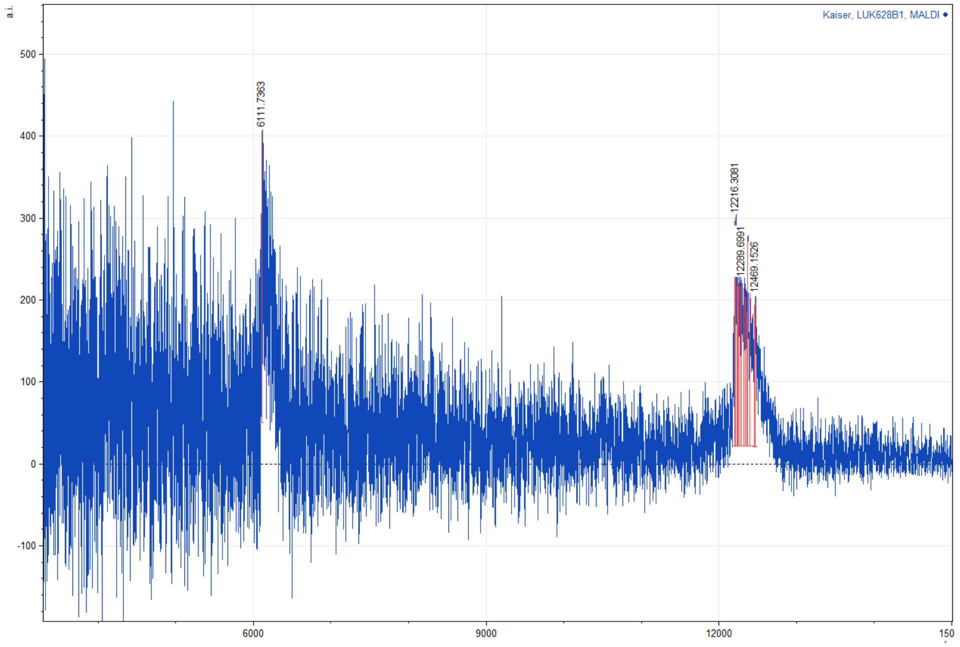

Figure S37. MALDI-TOF spectra of **31ON_C$^{\mathbf{Im}}$U$^{\mathbf{COOH}}$A$^{\mathbf{THT}}$G$^{\mathbf{OH}}$**: calculated 12175.8 Da; found 12216.3 Da; Δ = 40.5 Da

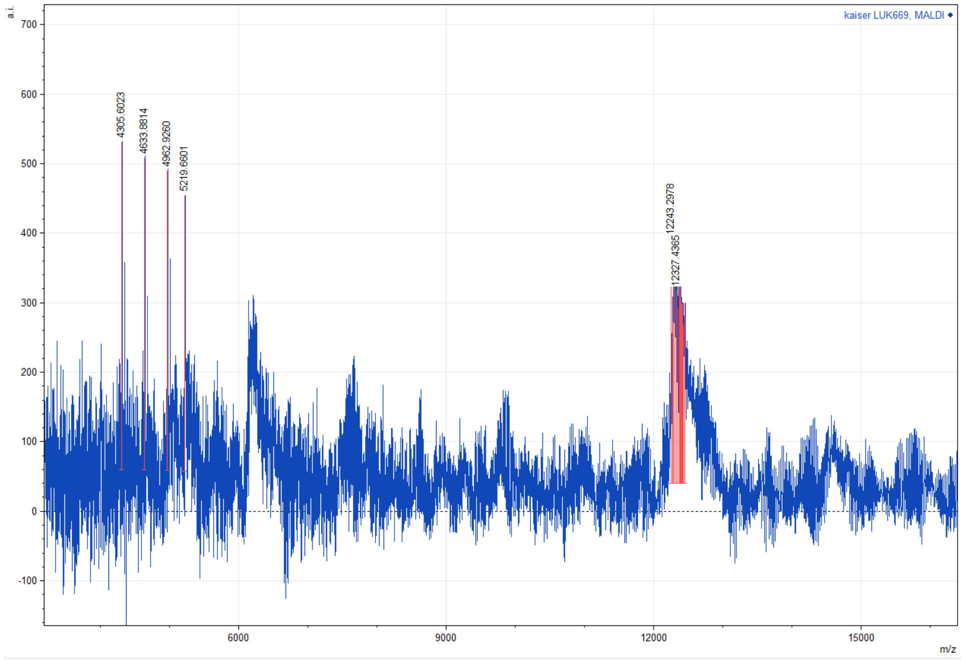

Figure S38. MALDI-TOF spectra of **31ON_C$^{\mathbf{Im}}$U$^{\mathbf{EPh}}$A$^{\mathbf{PSH}}$G$^{\mathbf{AiPr}}$**: calculated 12239.4 Da; found 12243.3 Da; Δ = 3.9 Da

4. Copies of NMR spectra

^{1}H, ^{13}C and $^{31}P\{^{1}H\}$ NMR spectra of dU^{COOH}TP

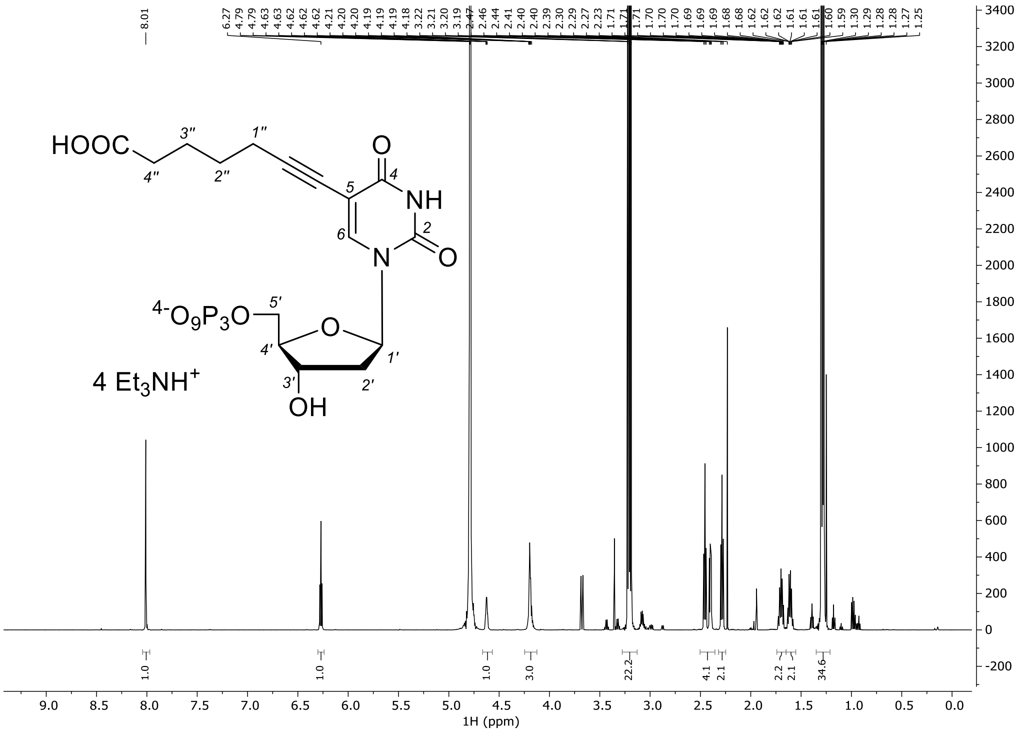

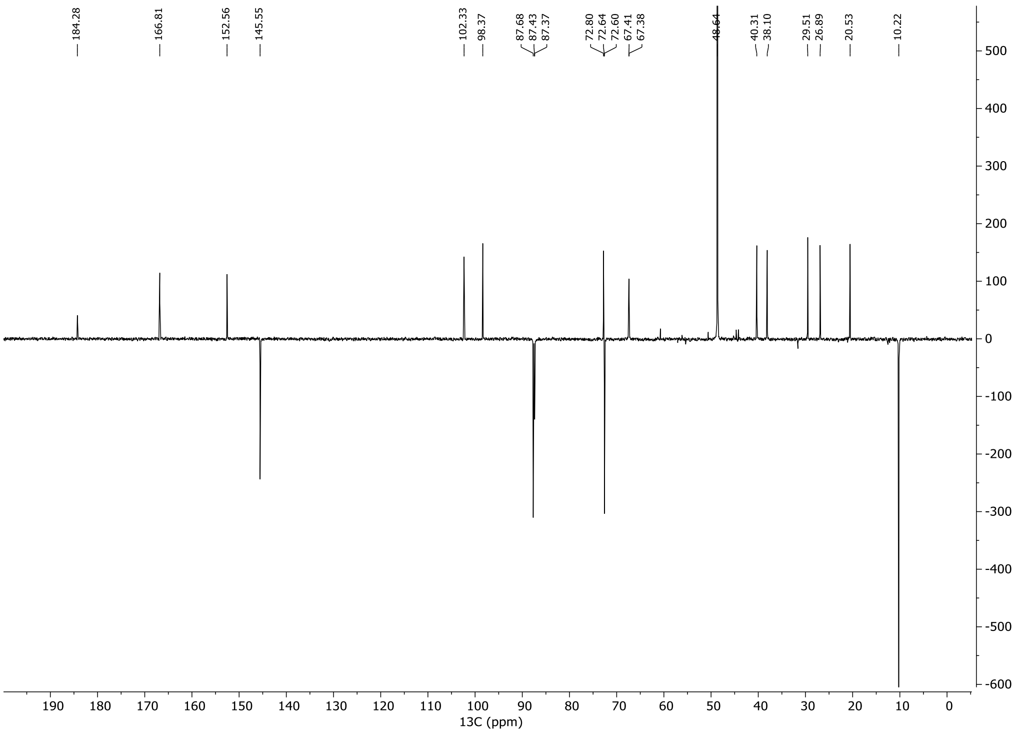

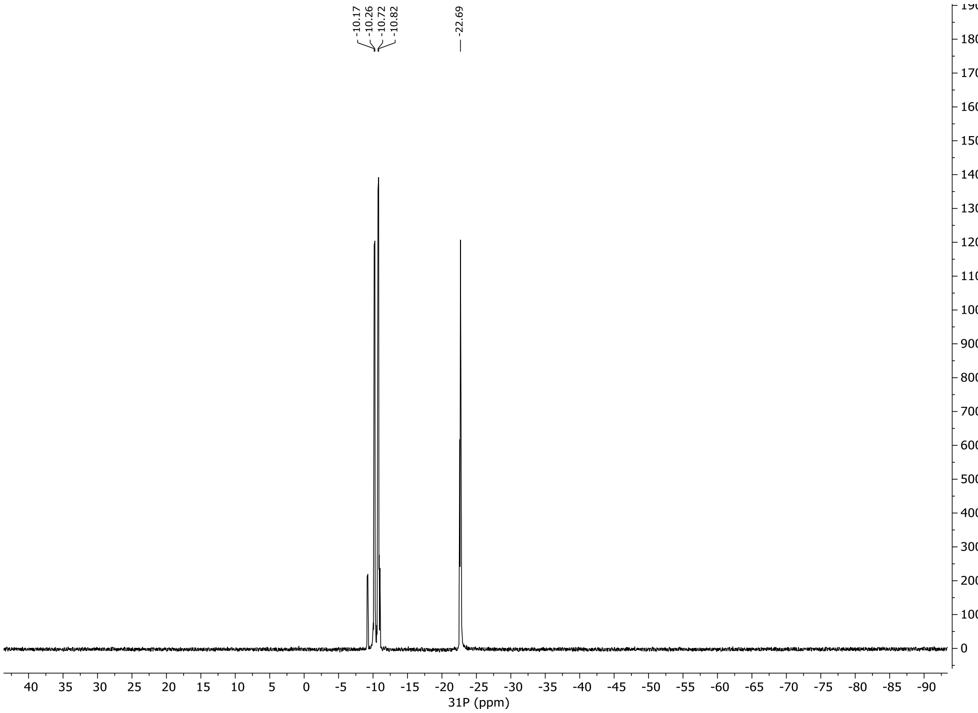

^{1}H, ^{13}C and $^{31}P\{^{1}H\}$ NMR spectra of dG^{OH}TP

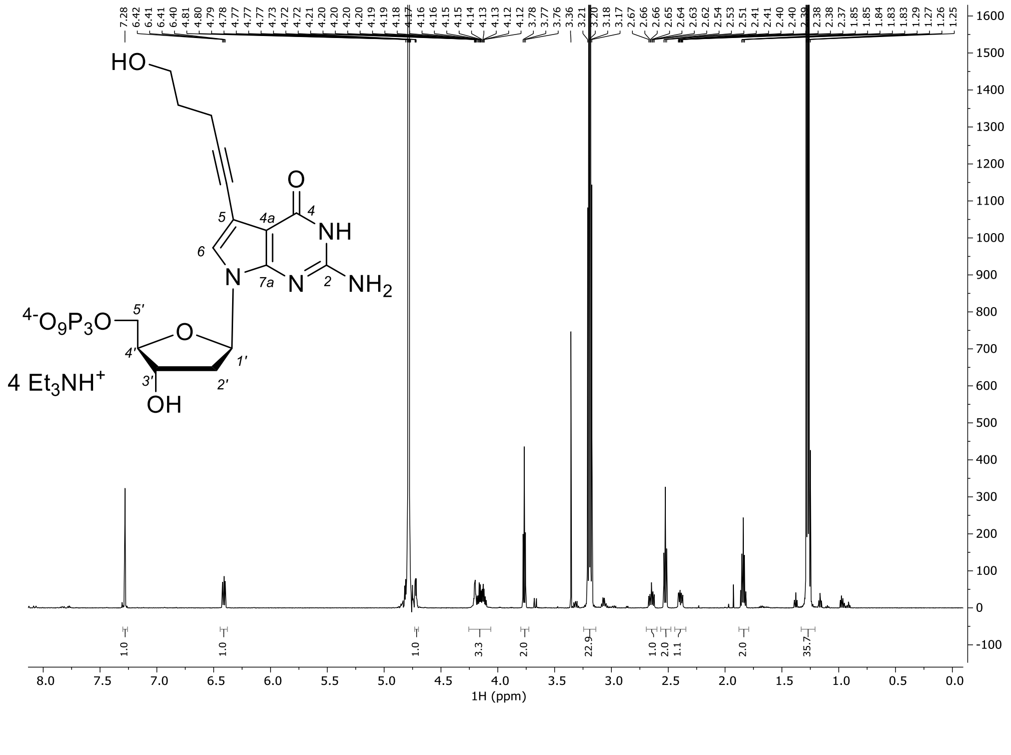

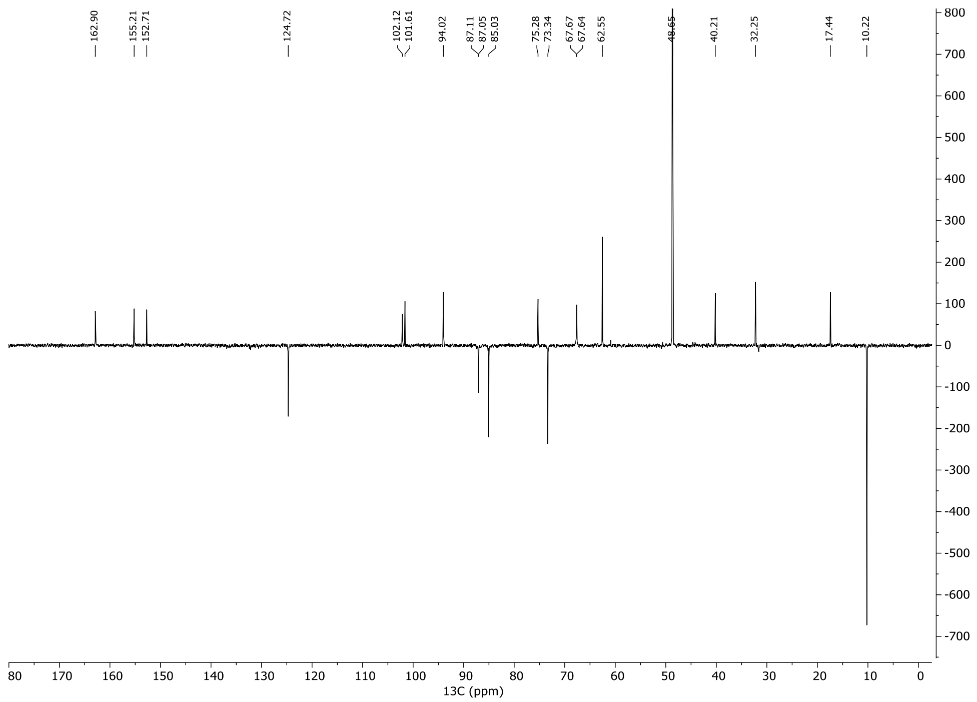
162.90
155.21
152.71
124.72
102.12
101.61
94.02
87.11
87.05
85.03
75.28
73.34
67.67
67.64
62.55
48.65
40.21
32.25
17.44
10.22
13C (ppm)

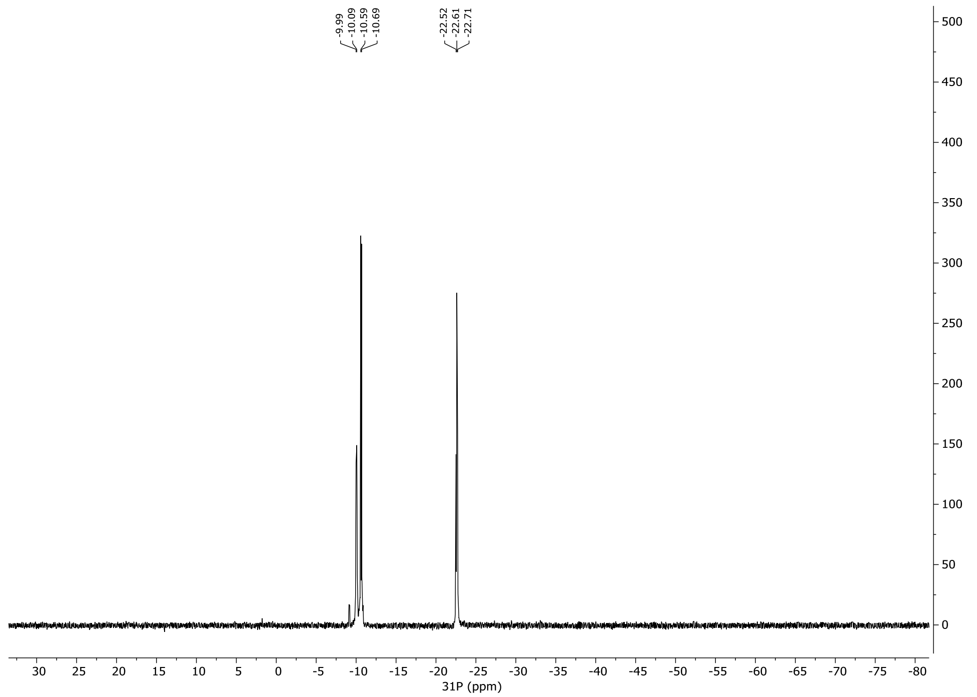
-9.99
-10.09
-10.59
-10.69
-22.52
-22.61
-22.71
31P (ppm)

^{1}H, ^{13}C and $^{31}P\{^{1}H\}$ NMR spectra of dC^{Im}TP

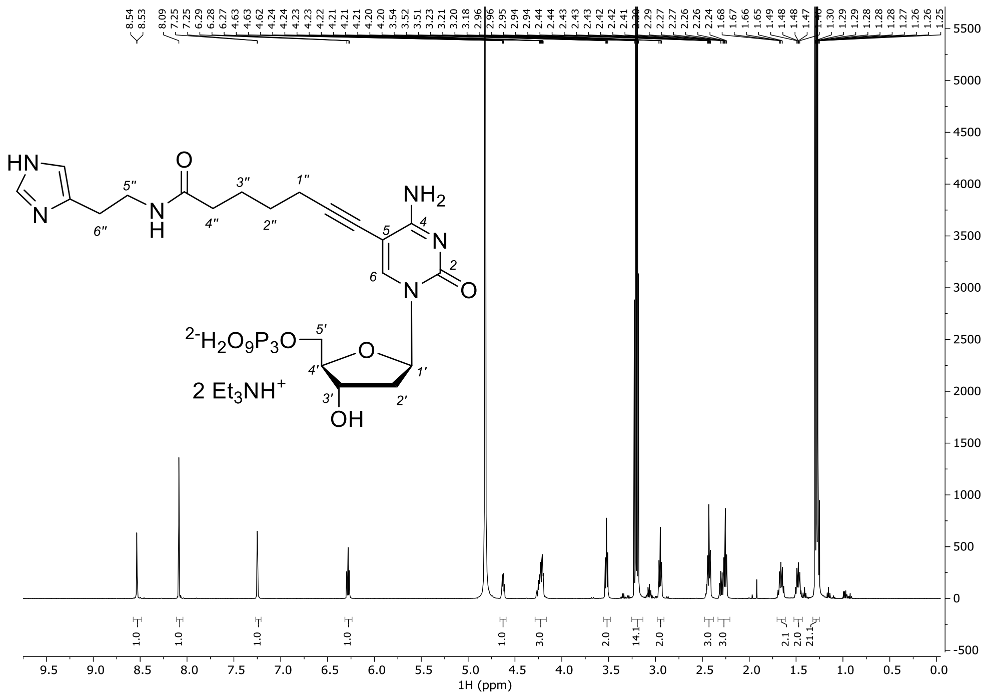

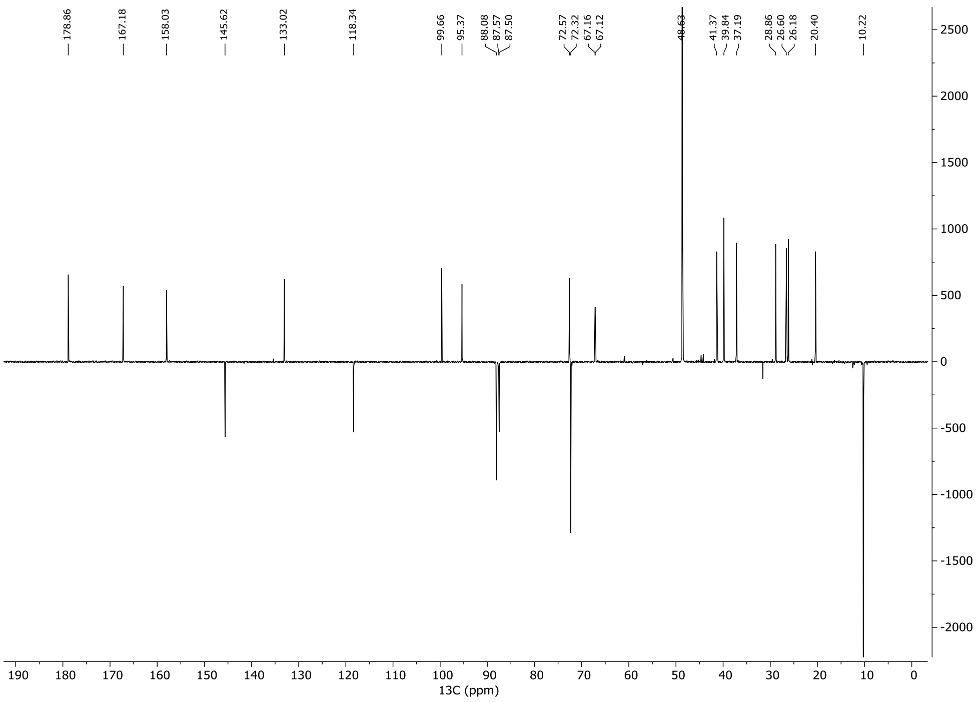

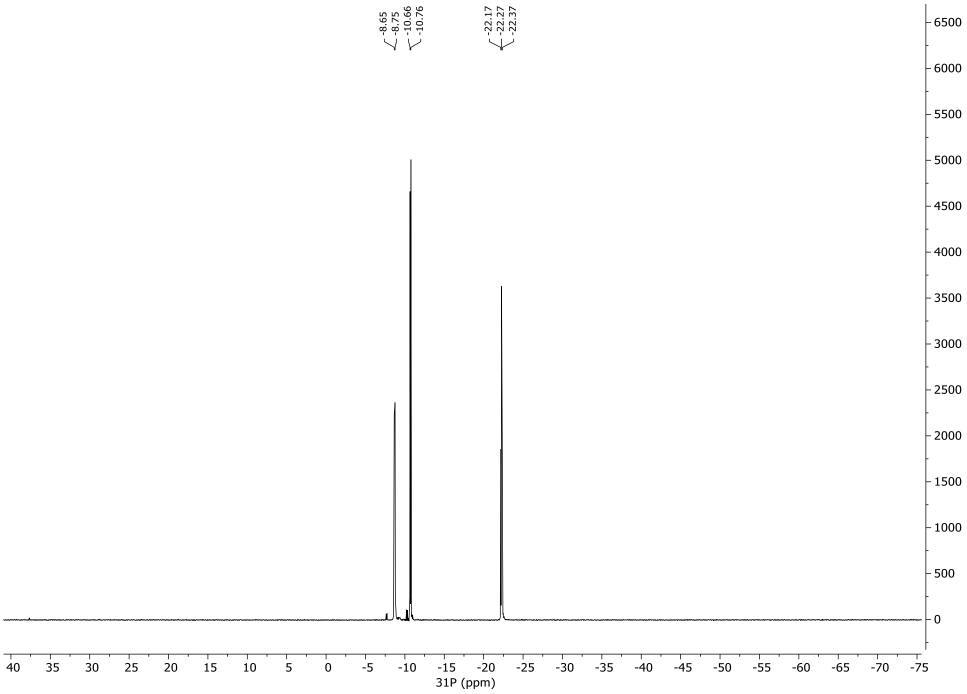

^{1}H and ^{13}C NMR spectra of N-(2-(1H-imidazol-4-yl)ethyl)hept-6-ynamide

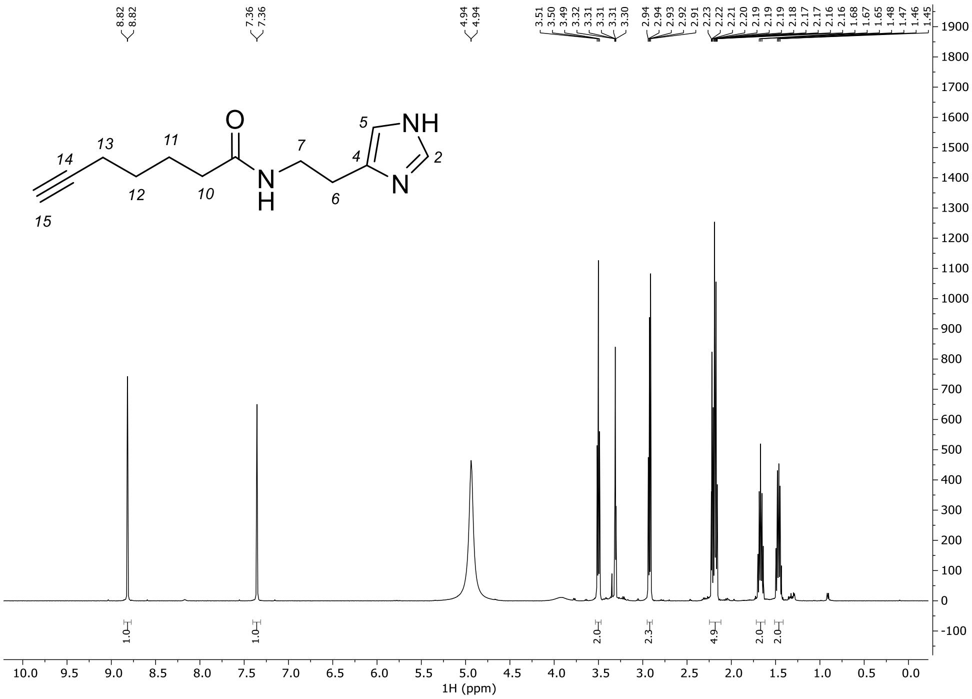

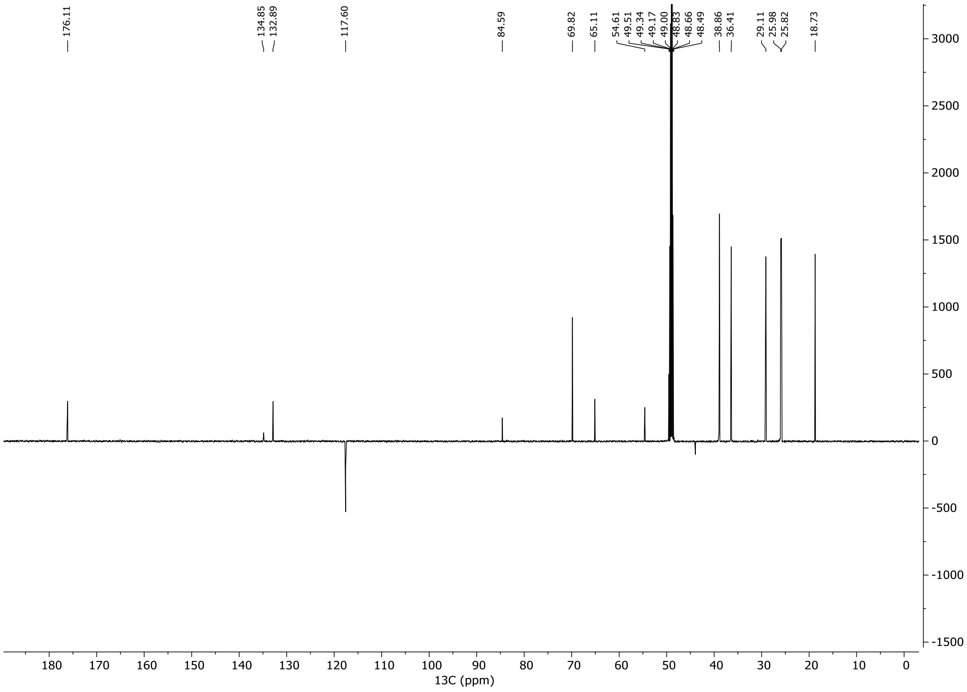

^{1}H and ^{13}C NMR spectra of dA^{ESAc}

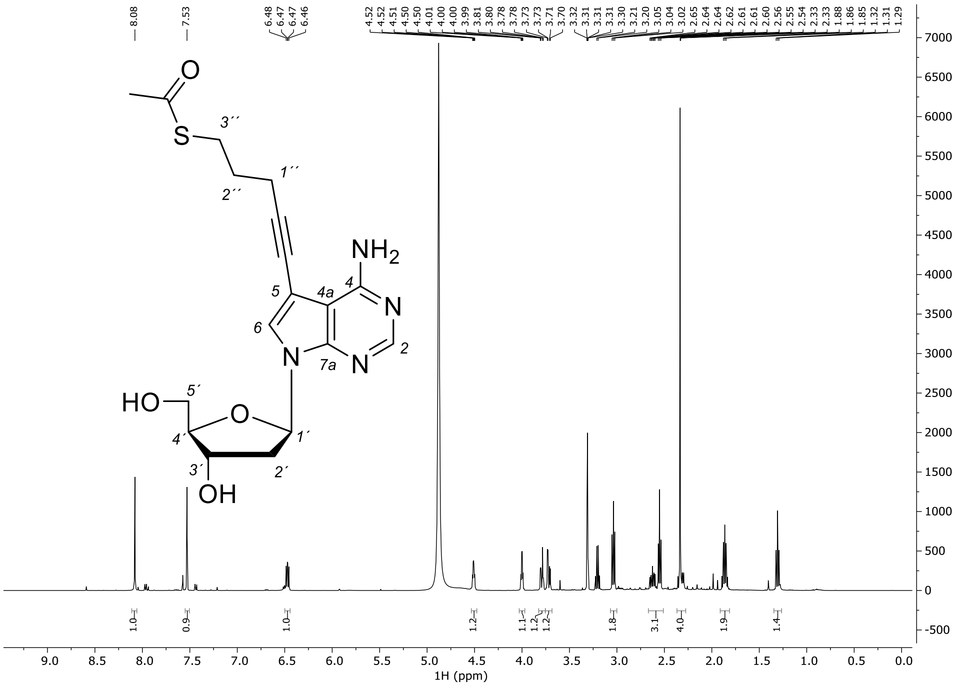

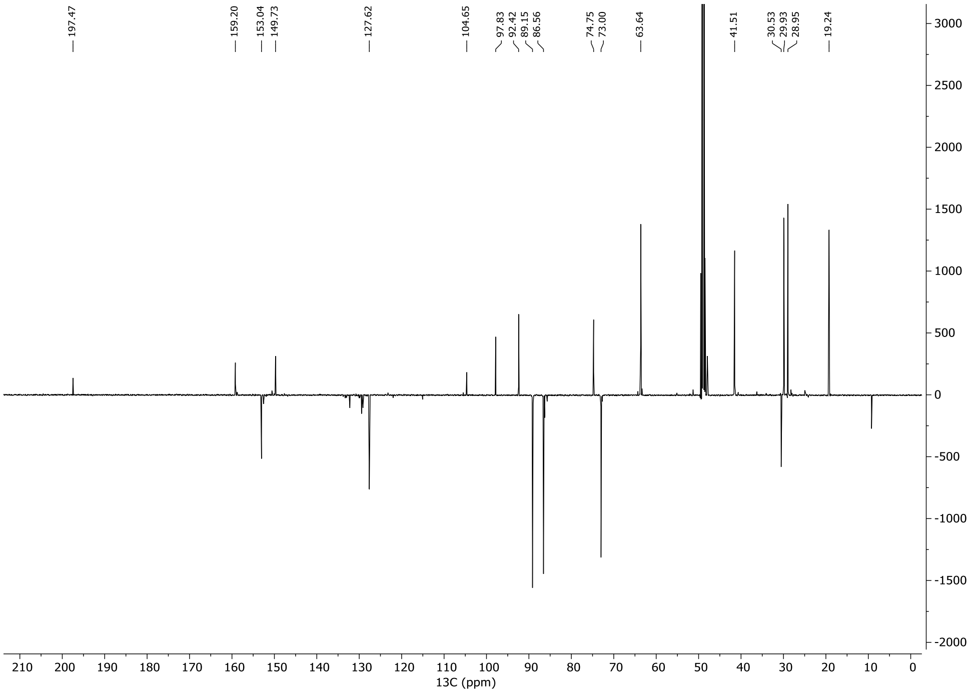

^{1}H, ^{13}C and $^{31}P\{^{1}H\}$ NMR spectra of dA^{ESAc}TP

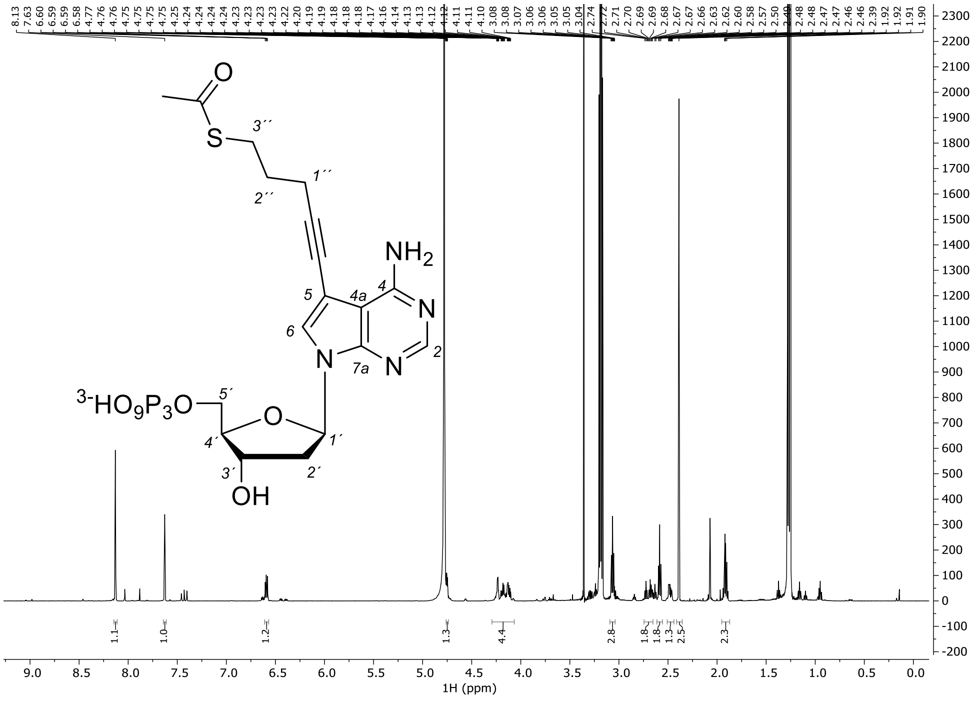

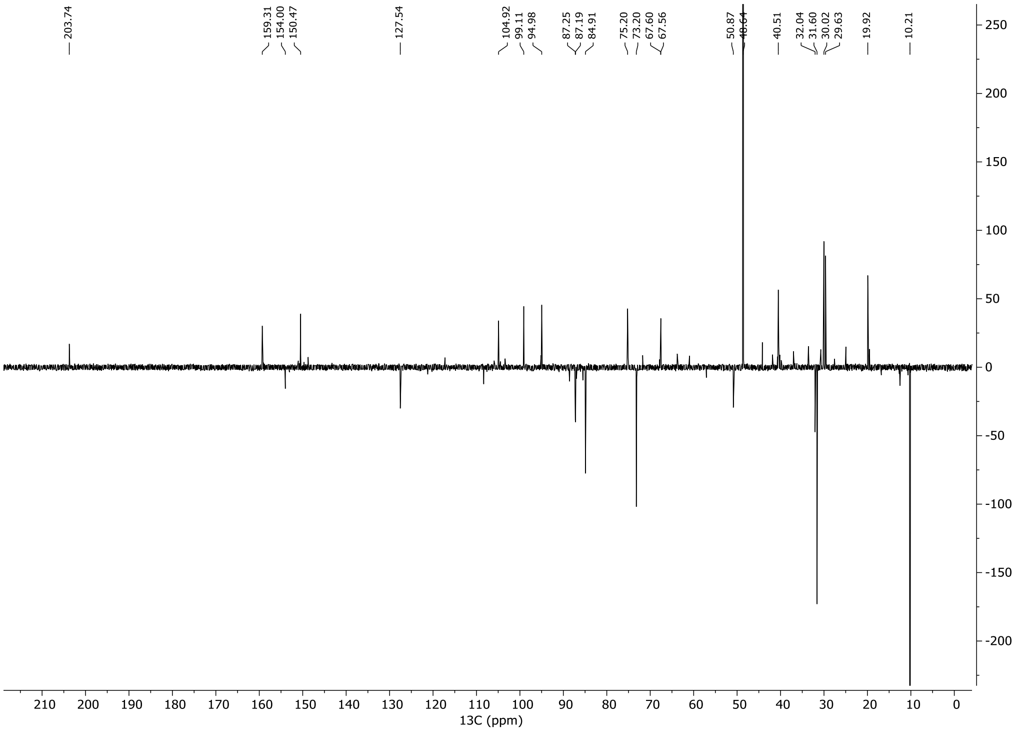
203.74
159.31
154.00
150.47
127.54
104.92
99.11
94.98
87.25
87.19
84.91
75.20
73.20
67.60
67.56
50.87
48.64
40.51
32.04
31.60
30.02
29.63
19.92
10.21
13C (ppm)

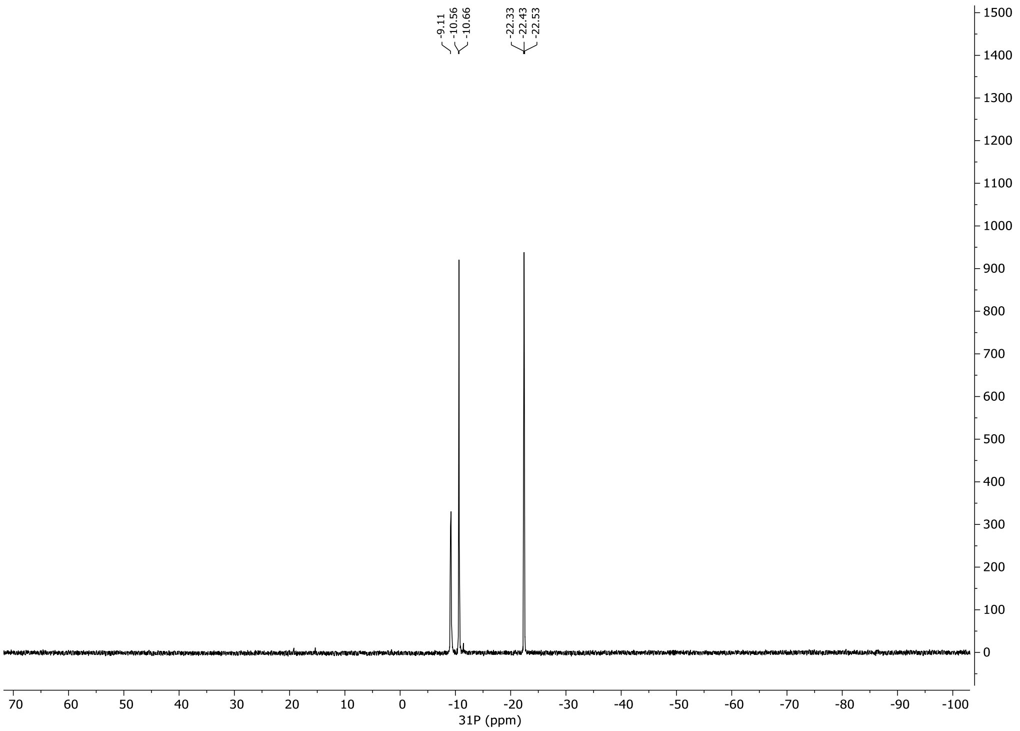
-9.11
-10.56
-10.66
-22.33
-22.43
-22.53
31P (ppm)

^{1}H, ^{13}C and $^{31}P\{^{1}H\}$ NMR spectra of dA^{THT}TP

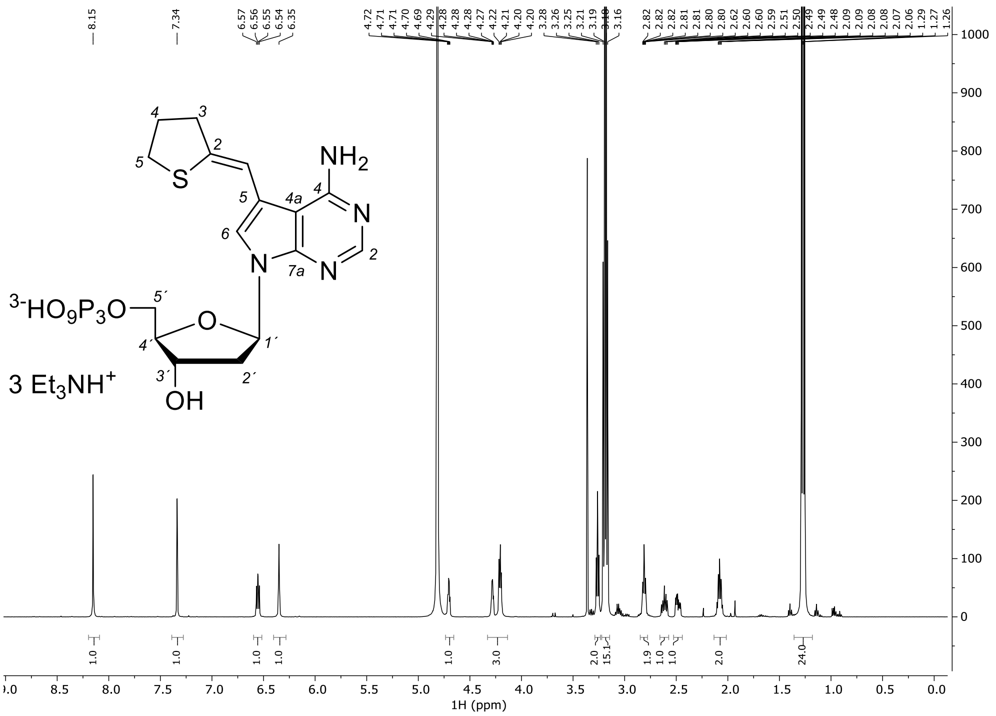

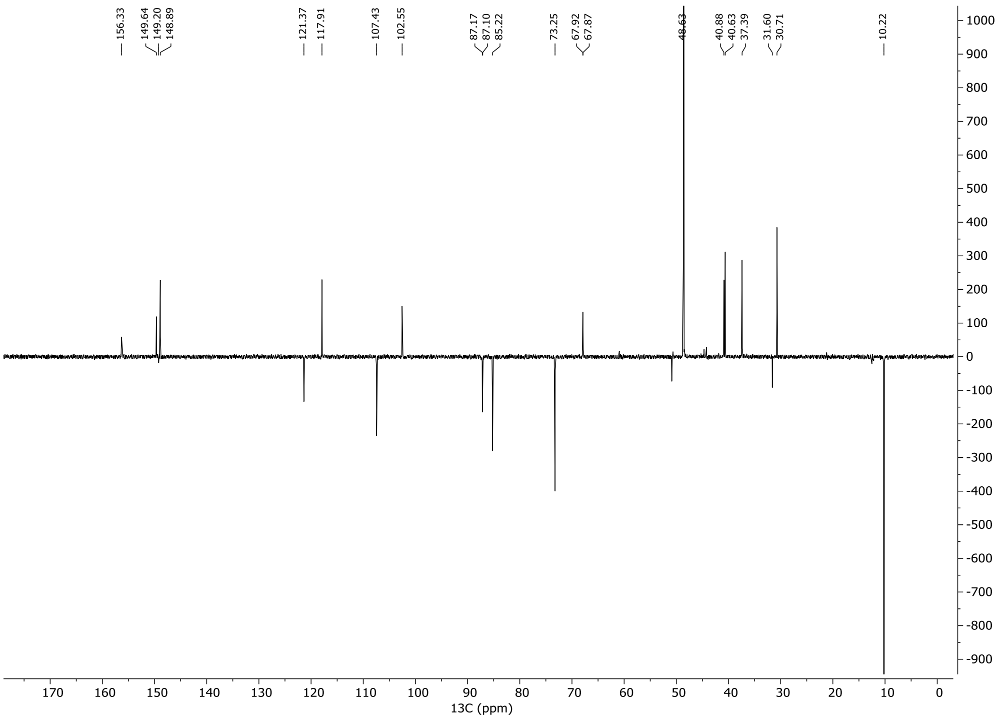

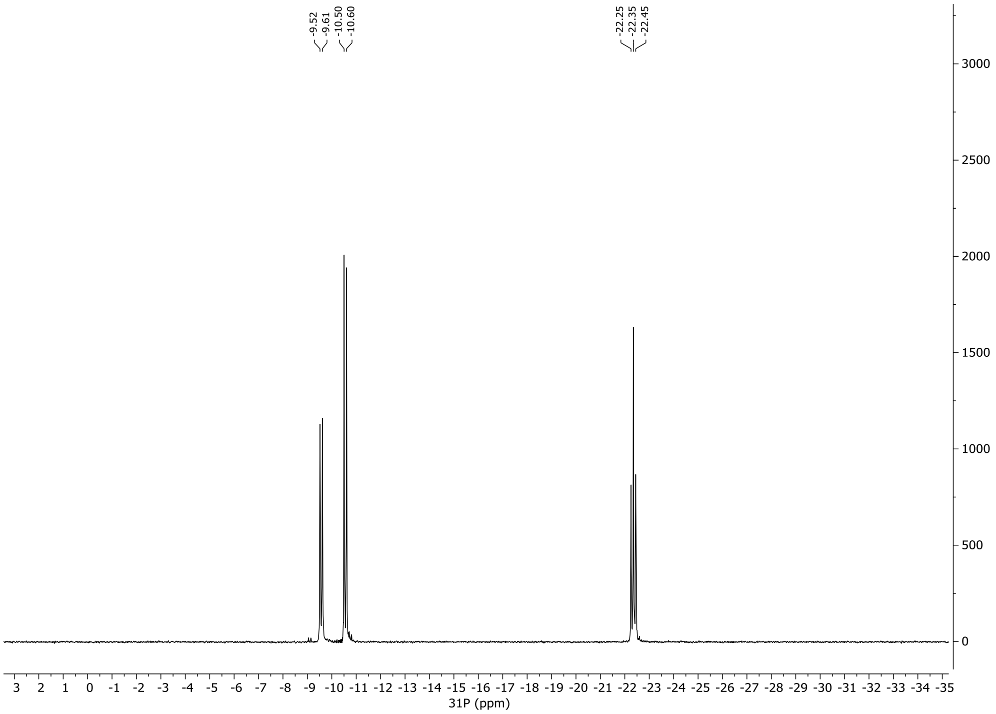

^{1}H, ^{13}C and $^{31}P\{^{1}H\}$ NMR spectra of dA^{ESH}TP

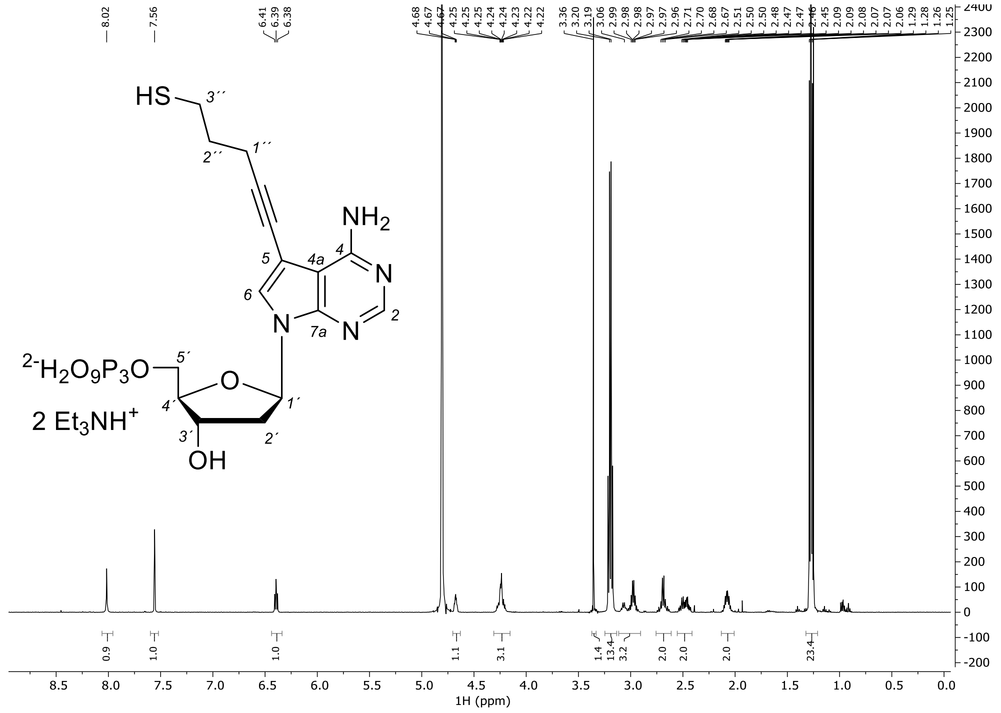

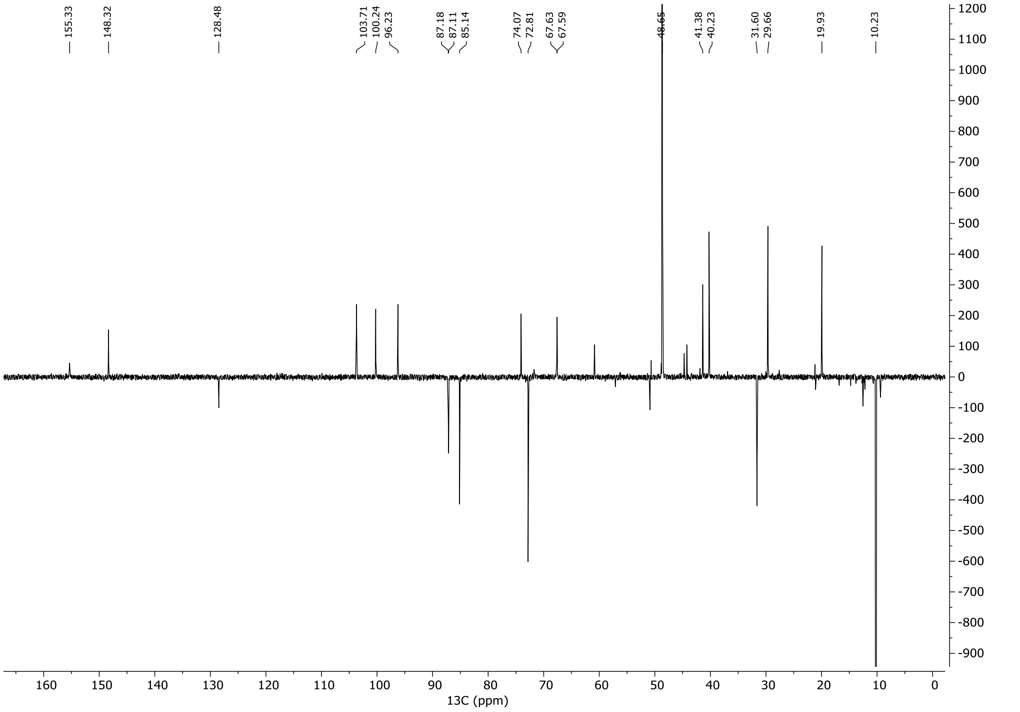
155.33
148.32
128.48
103.71
100.24
96.23
87.18
87.11
85.14
74.07
72.81
67.63
67.59
48.65
41.38
40.23
31.60
29.66
19.93
10.23
13C (ppm)

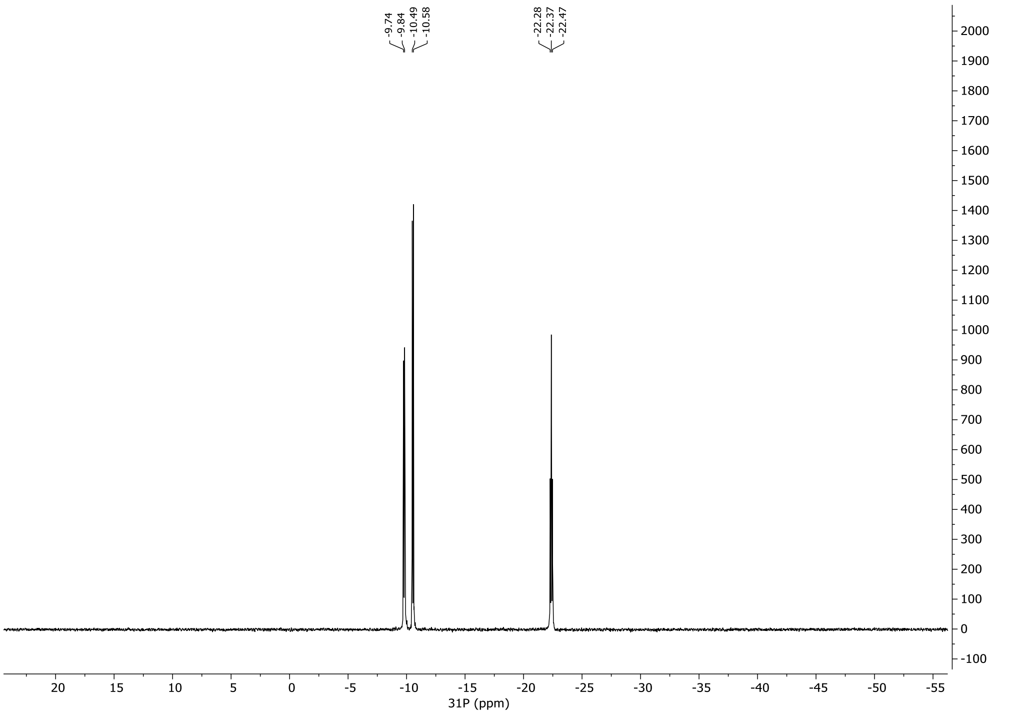
-9.74
-9.84
-10.49
-10.58
-22.28
-22.37
-22.47
31P (ppm)

^{1}H and ^{13}C NMR spectra of dA^{THT}

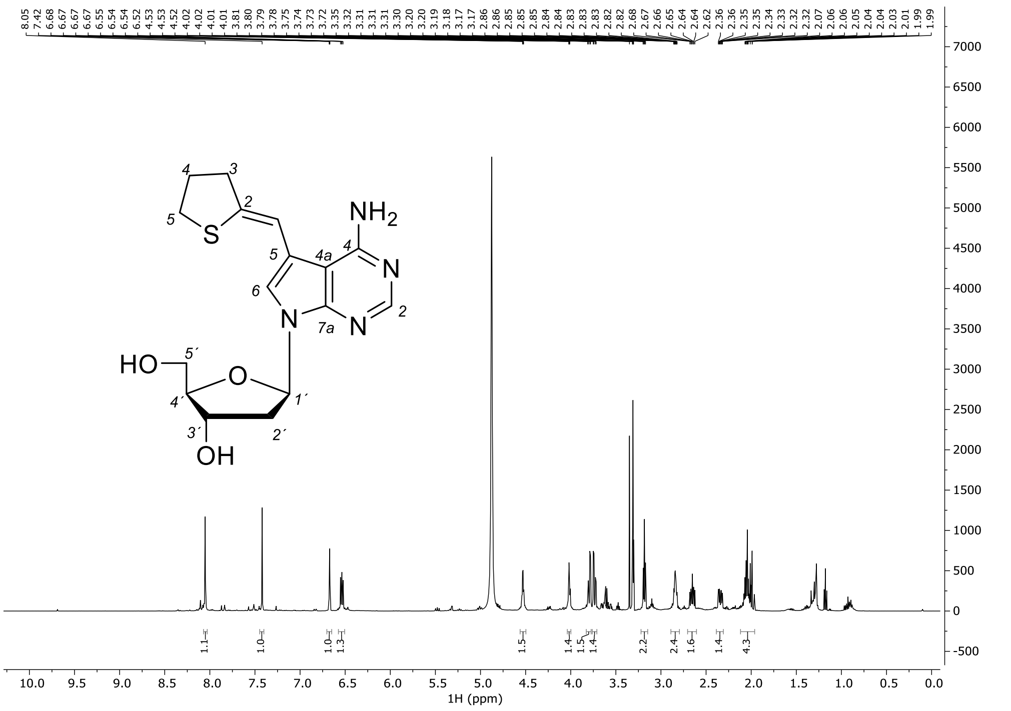

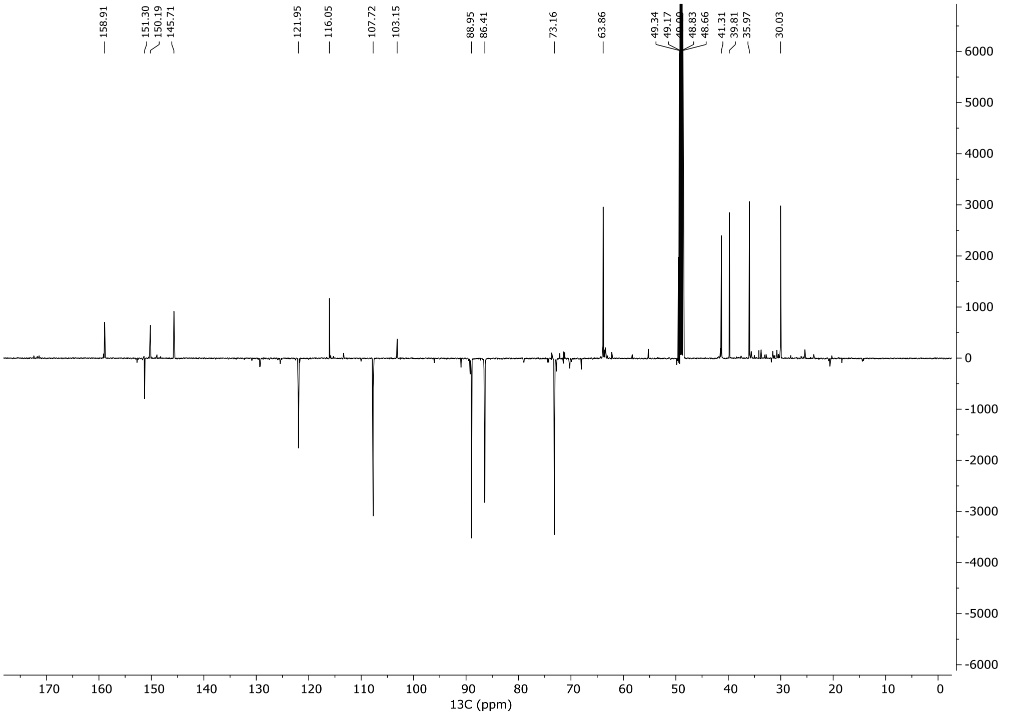

^{1}H, ^{13}C and $^{31P}\{^{1}H\}$ NMR spectra of dA^{ASH}TP

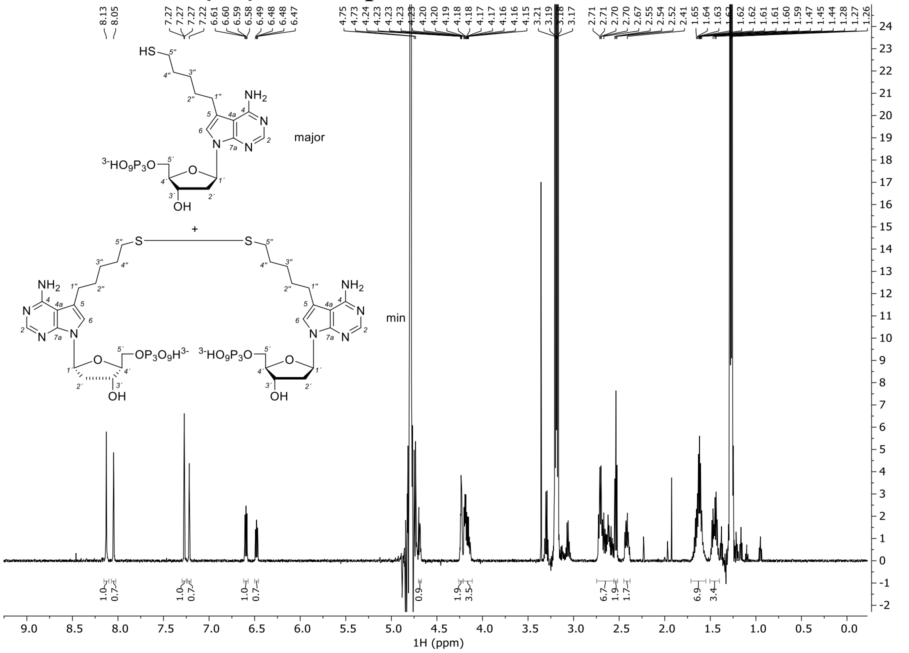

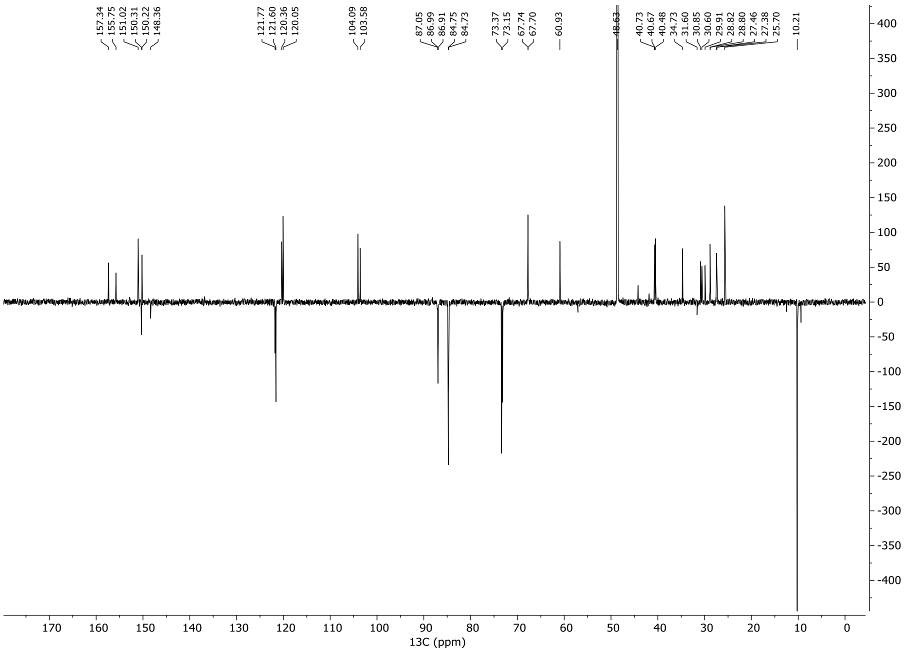

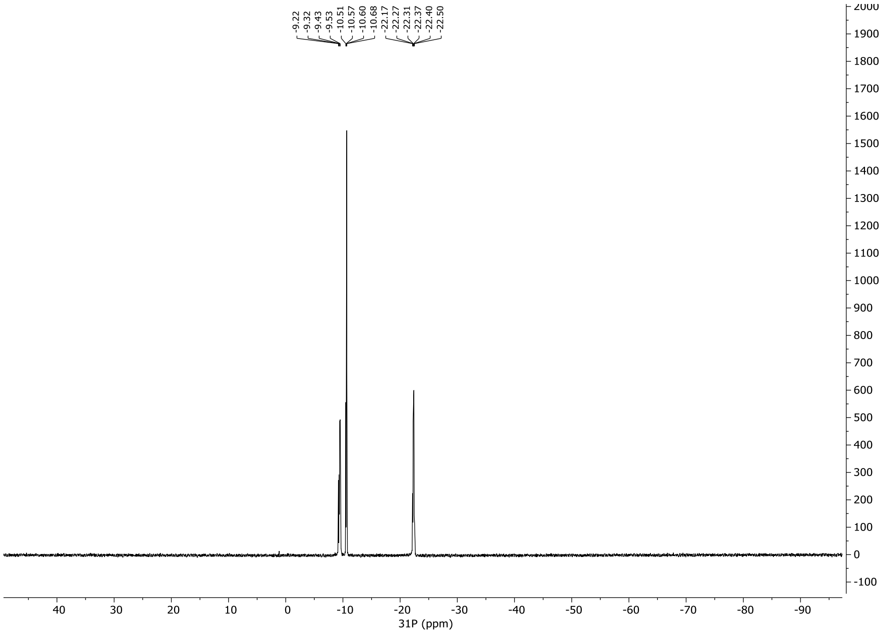

^{1}H and ^{13}C NMR spectra of dA^{ASSA}dA

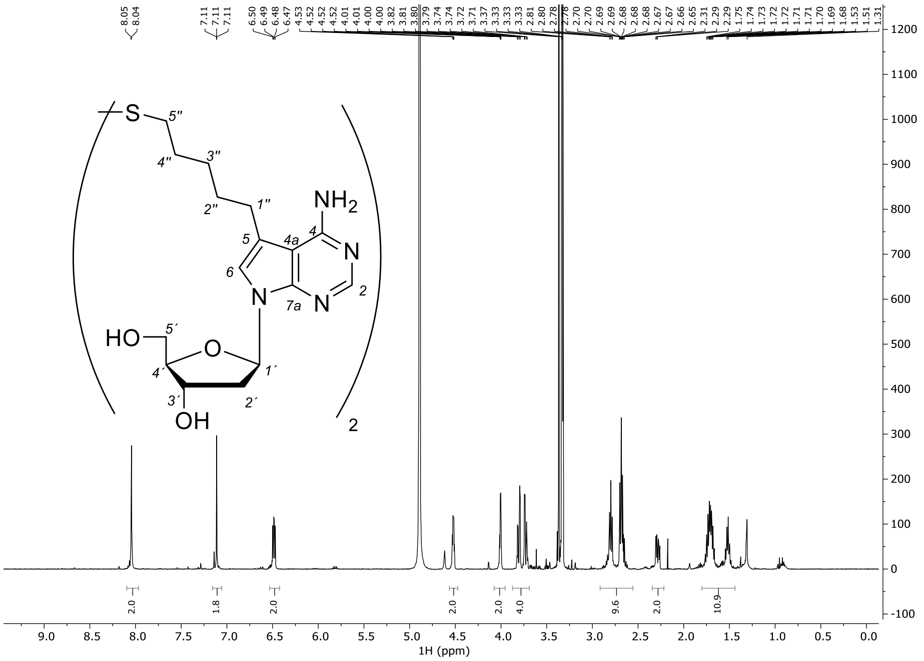

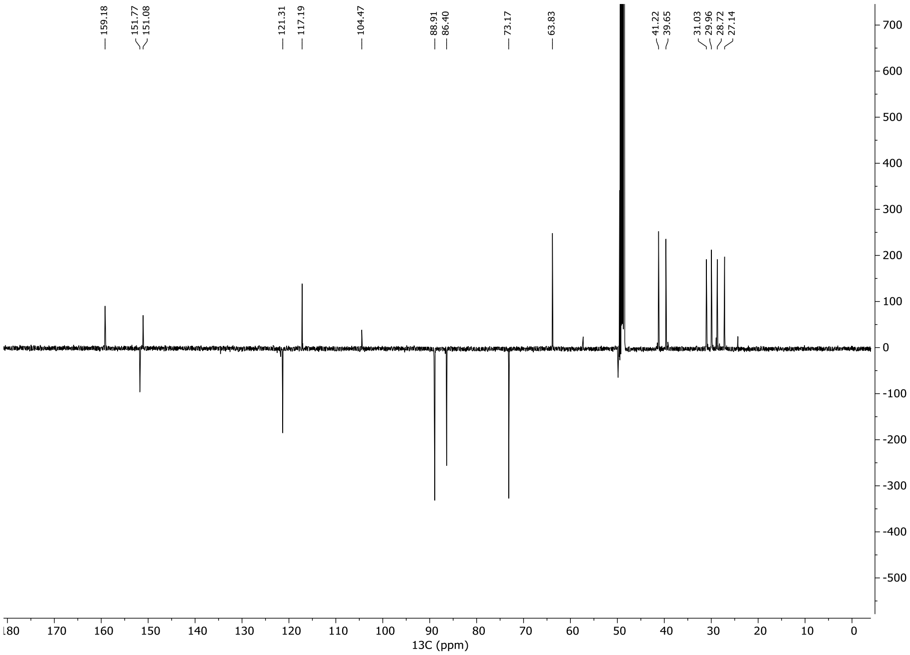

^{1}H and ^{13}C NMR spectra of dA^{ASH}

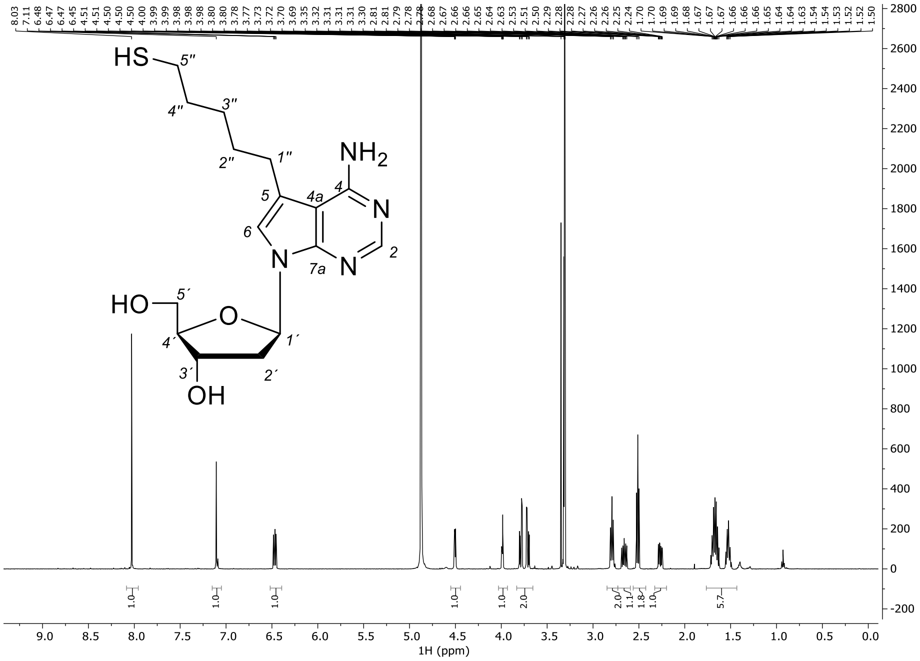

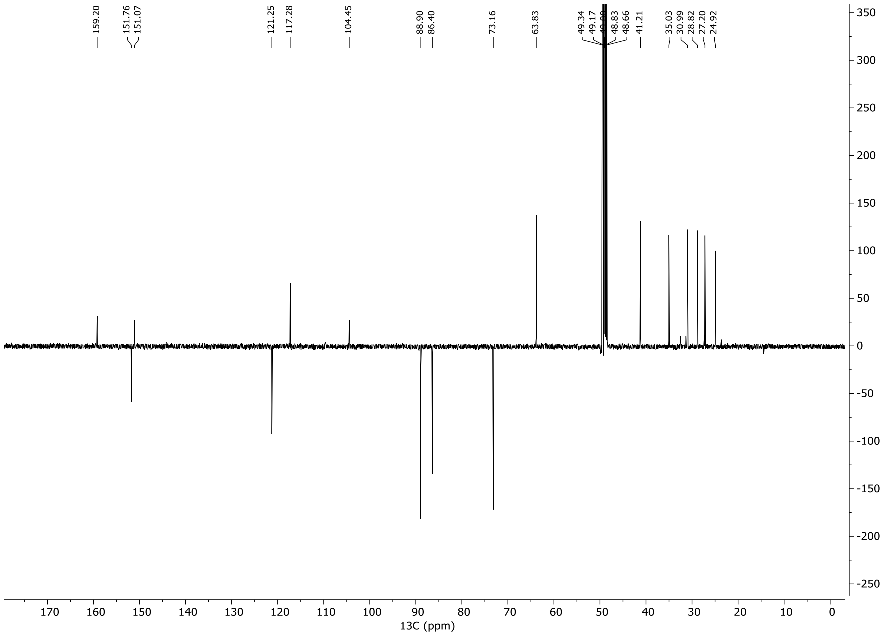

^{1}H and ^{13}C NMR spectra of dA^{PSAc}

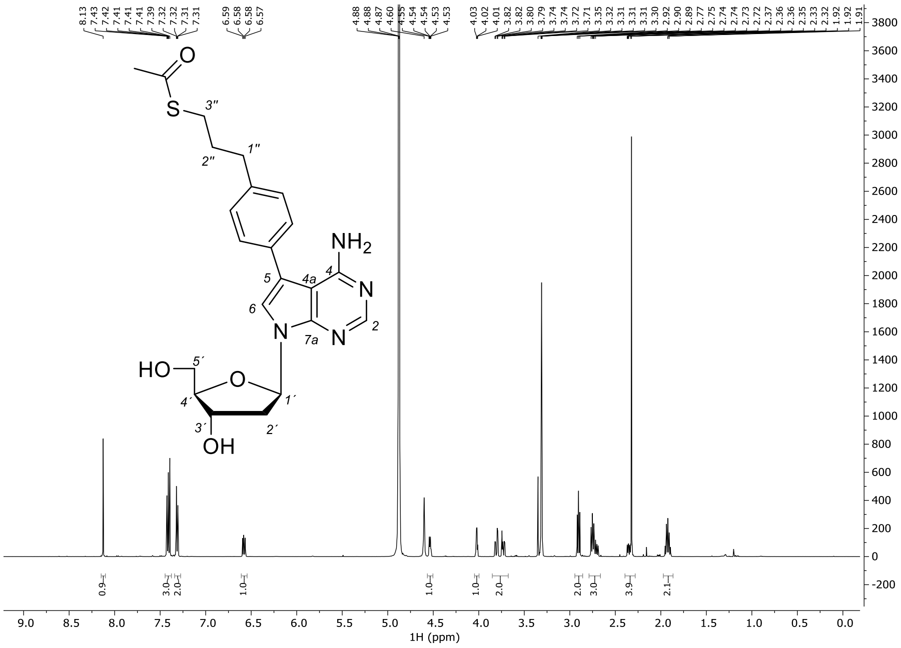

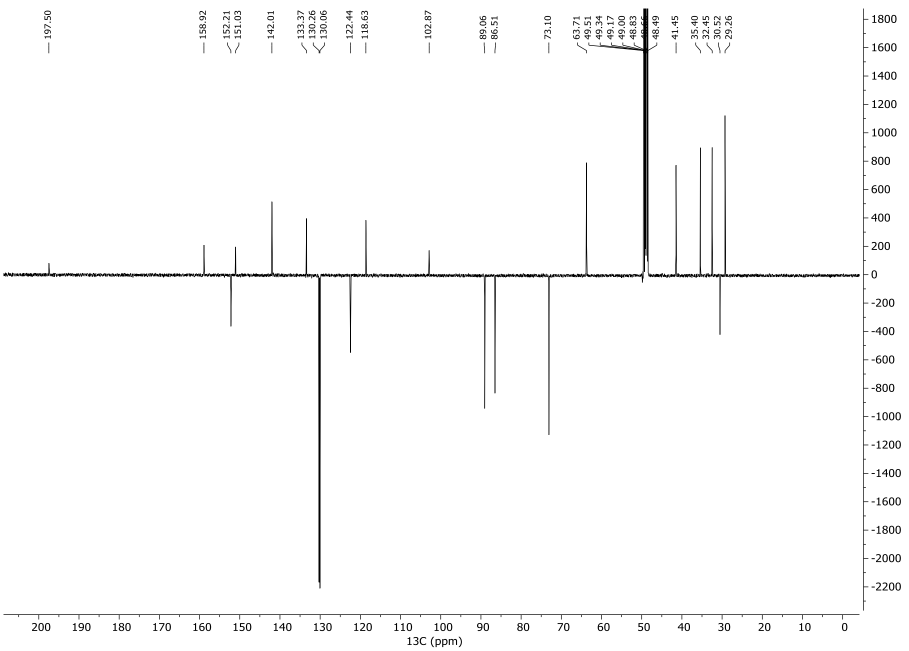

^{1}H, ^{13}C and $^{31}P\{^{1}H\}$ NMR spectra of $dA^{PSAc}TP$

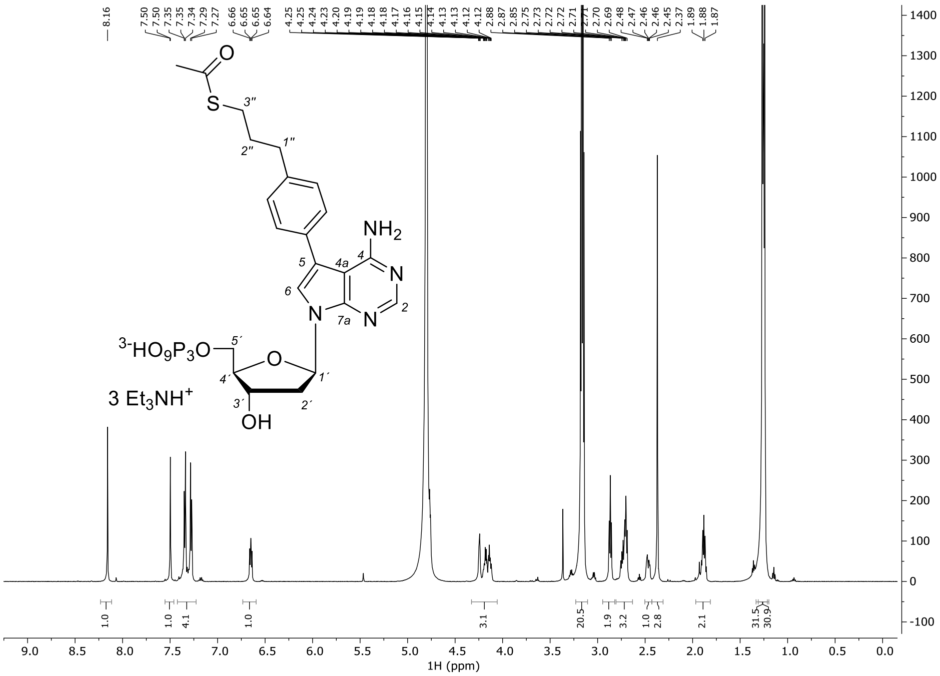

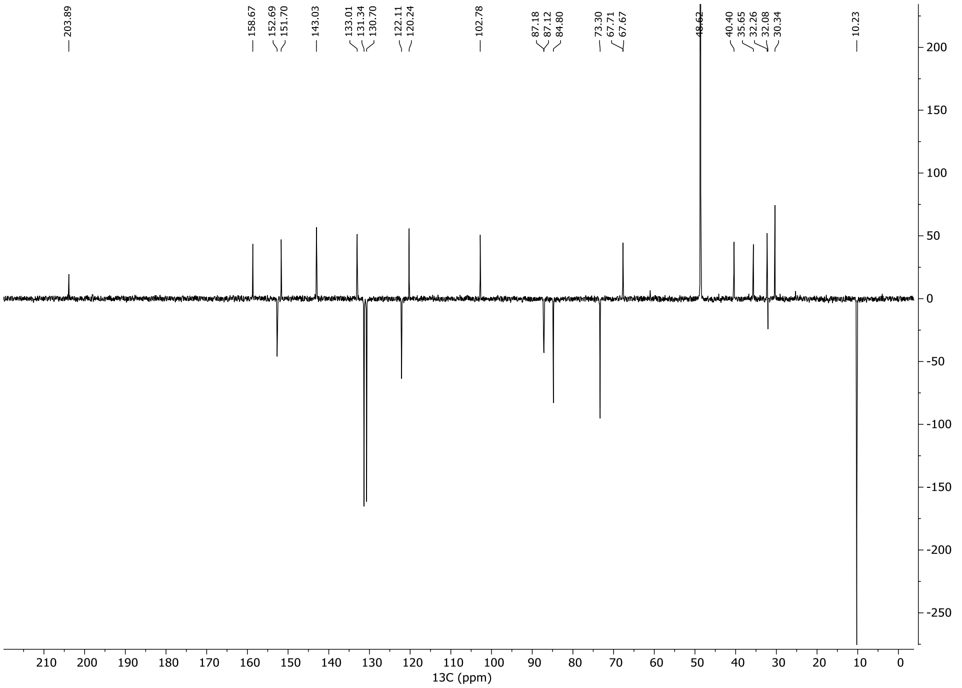
203.89
158.67
152.69
151.70
143.03
133.01
131.34
130.70
122.11
120.24
102.78
87.18
87.12
84.80
73.30
67.71
67.67
48.62
40.40
35.65
32.26
32.08
30.34
10.23
13C (ppm)

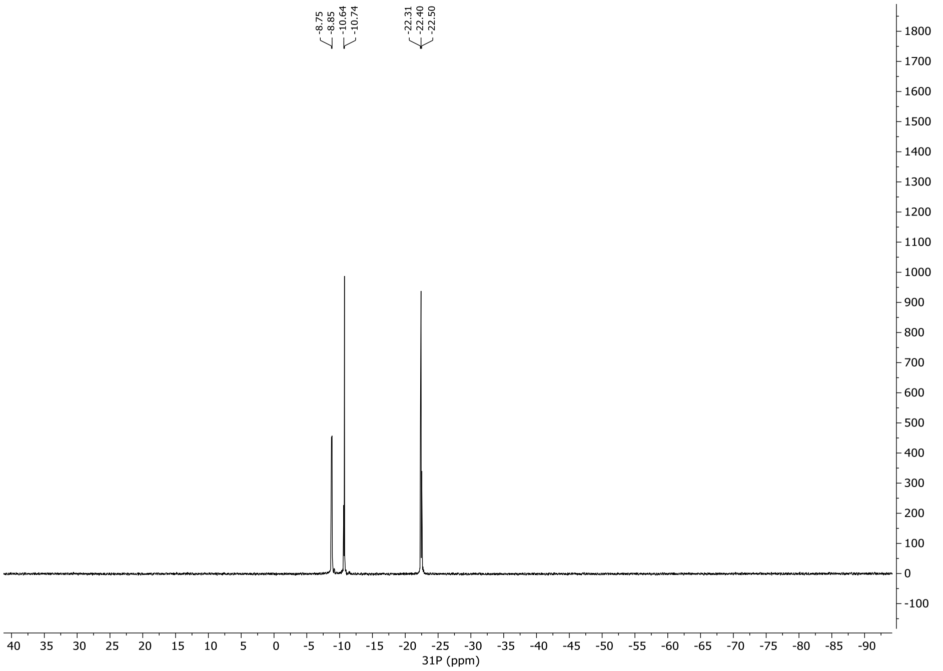
-8.75
-8.85
-10.64
-10.74
-22.31
-22.40
-22.50
31P (ppm)

^{1}H, ^{13}C and ^{31}P{^{1}H} NMR spectra of dA^{PSH}TP

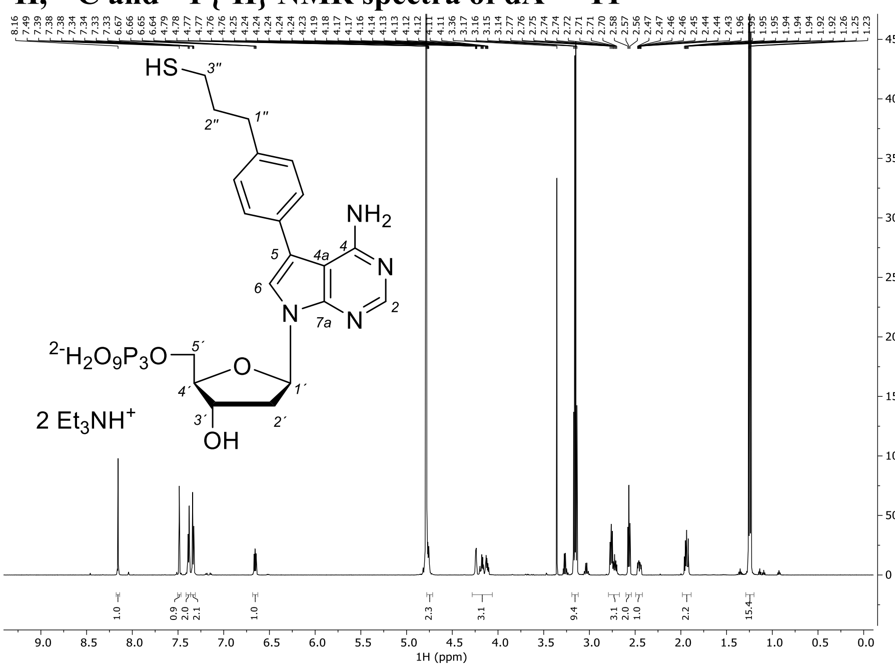

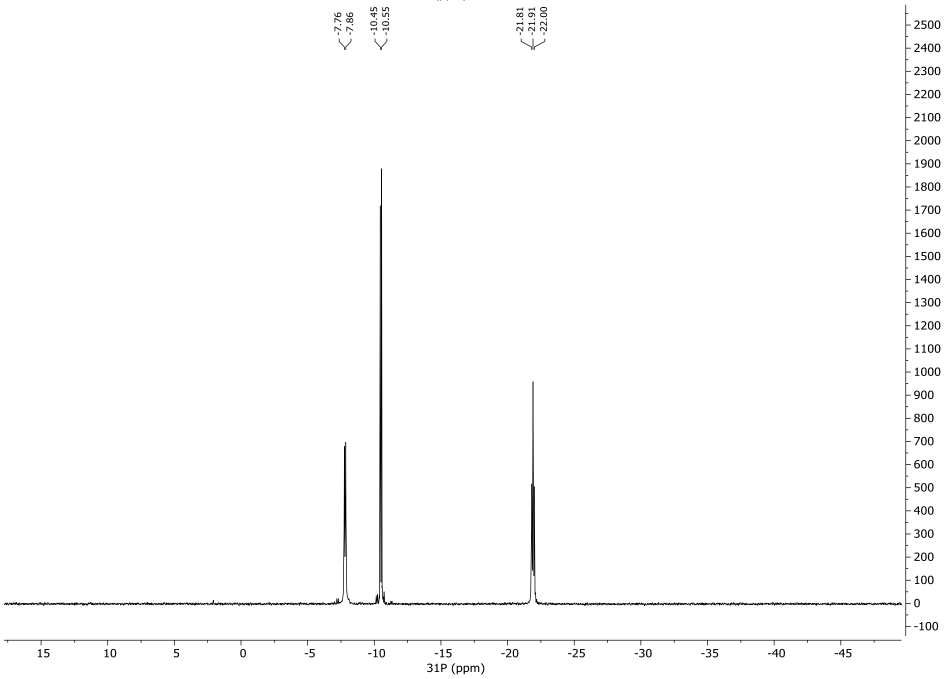

^{1}H and ^{13}C NMR spectra of dA^{PSH}

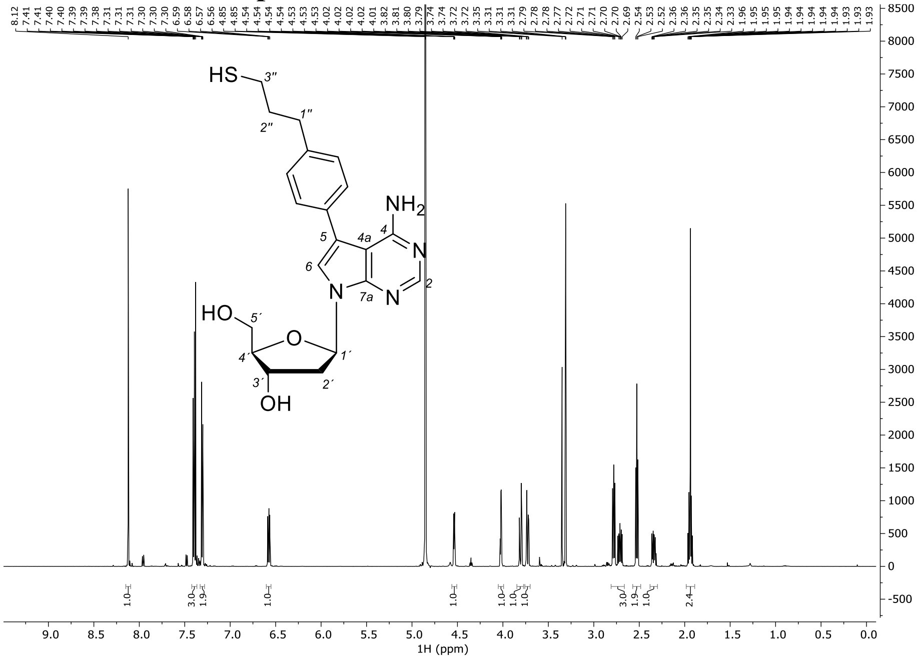

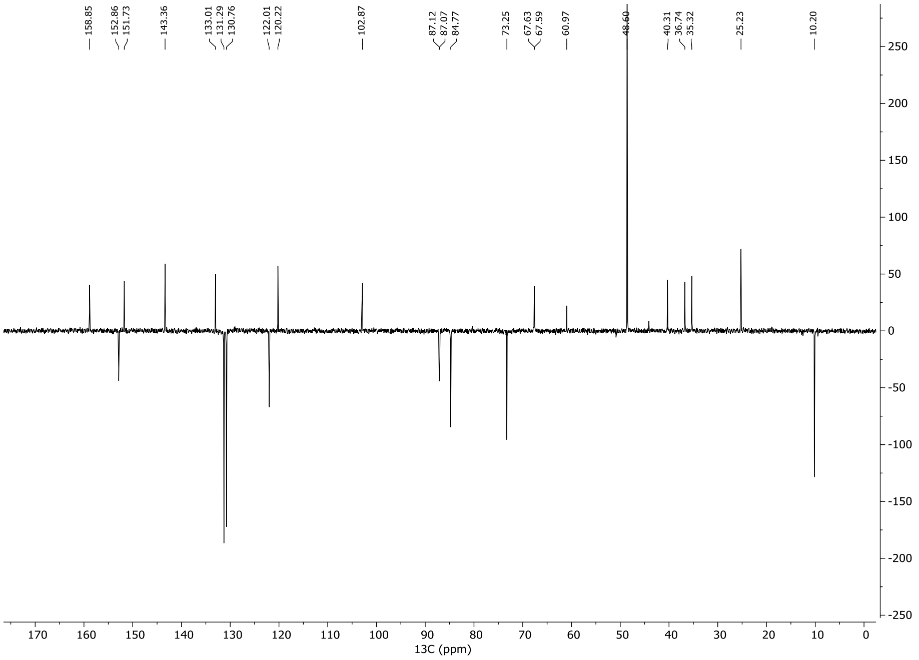

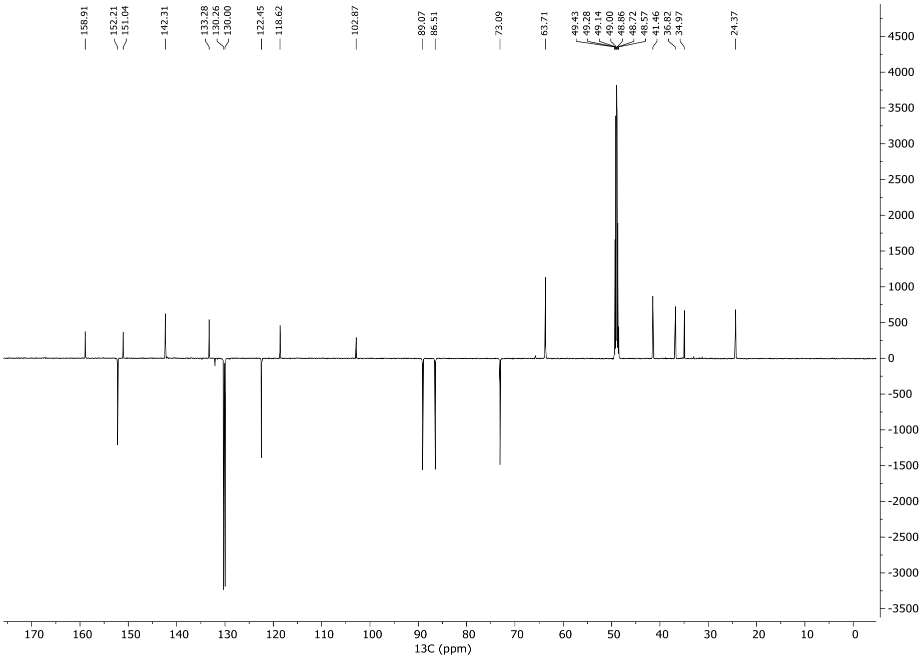

158.91
152.21
151.04
142.31
133.28
130.26
130.00
122.45
118.62
102.87
89.07
86.51
73.09
63.71
49.43
49.28
49.14
49.00
48.86
48.72
48.57
41.46
36.82
34.97
24.37
13C (ppm)

5. References

1. Seela, F., Zulauf, M. *Synthesis*, **1996**, 726 —730
2. Le, B.H., Koo, J.Ch., Joo, H.N., Seo, Y.J. *Bioorg. Med. Chem.* **2017**, 25, 591-3596
3. Cahová, H., Havran, L., Brázdilová, P., Pivoňková, H., Pohl, R., Fojta, M., Hocek, M. *Angew. Chem. Int. Ed*. **2008**, 47, 1433-7851.
4. Sýkorová, V., Tichý, M., Hocek, M. *ChemBioChem* **2022**, *23*, e202100608.
5. Benati, C. L., Calestani, G., Leardini, R., Minozzi, M., Nanni, D., Spagnolo, P. and Strazzari, S. *Org. Lett.* **2003**, 5, 1313-1316.
6. Betz, K. N., Chiappini, N. D., Du Bois, J. *Org. Lett.* **2020**, *22*, 1687-1691.
